# Seeding the aggregation of TDP-43 requires post-fibrillization proteolytic cleavage

**DOI:** 10.1038/s41593-023-01341-4

**Published:** 2023-05-29

**Authors:** Senthil T. Kumar, Sergey Nazarov, Sílvia Porta, Niran Maharjan, Urszula Cendrowska, Malek Kabani, Francesco Finamore, Yan Xu, Virginia M.-Y. Lee, Hilal A. Lashuel

**Affiliations:** 1 Laboratory of Molecular and Chemical Biology of Neurodegeneration, Brain Mind Institute, EPFL, Lausanne, Switzerland; 2 Center for Neurodegenerative Disease Research (CNDR), Department of Pathology and Laboratory Medicine, University of Pennsylvania, Perelman School of Medicine, Philadelphia, PA USA

**Keywords:** Amyotrophic lateral sclerosis, Molecular neuroscience, Structural biology

## Abstract

Despite the strong evidence linking the transactive response DNA-binding protein 43 (TDP-43) aggregation to the pathogenesis of frontotemporal lobar degeneration with TDP-43, amyotrophic lateral sclerosis and several neurodegenerative diseases, our knowledge of the sequence and structural determinants of its aggregation and neurotoxicity remains incomplete. Herein, we present a new method for producing recombinant full-length TDP-43 filaments that exhibit sequence and morphological features similar to those of brain-derived TDP-43 filaments. We show that TDP-43 filaments contain a β-sheet-rich helical amyloid core that is fully buried by the flanking structured domains of the protein. We demonstrate that the proteolytic cleavage of TDP-43 filaments and exposure of this amyloid core are necessary for propagating TDP-43 pathology and enhancing the seeding of brain-derived TDP-43 aggregates. Only TDP-43 filaments with exposed amyloid core efficiently seeded the aggregation of endogenous TDP-43 in cells. These findings suggest that inhibiting the enzymes mediating cleavage of TDP-43 aggregates represents a viable disease-modifying strategy to slow the progression of amyotrophic lateral sclerosis and other TDP-43 proteinopathies.

## Abstract (web summary)

Reconstitution of TDP-43 filaments that exhibit sequence and morphological features similar to those found in the brain helps to uncover a new mechanism for the formation and propagation of pathology in amyotrophic lateral sclerosis and other neurodegenerative diseases.

## Main

Amyotrophic lateral sclerosis (ALS) and frontotemporal lobar degeneration (FTLD) are two of the most common debilitating neurodegenerative diseases (NDs) in people aged between 45 and 65 years^1^. The pathological hallmark of these diseases is the presence of proteinaceous cytoplasmic inclusions in degenerating neurons. TDP-43 is the primary component of the cytoplasmic inclusions in ~97% of ALS and ~45% of FTLD (that is, FTLD-TDP) cases^2–4^.

Increasing evidence suggests that cellular changes leading to TDP-43 mislocalization and aggregation in the cytoplasm resulting in the gain of toxic functions that drive neurodegeneration in ALS and FTLD-TDP^5,6^. Furthermore, TDP-43 aggregates of distinct properties have been isolated from ALS and FTLD-TDP brains and are increasingly commonly found in the brains of patients suffering from other NDs^7,8^. Therefore, understanding the molecular, structural and cellular determinants of TDP-43 aggregation is essential to unravel its role in NDs and developing novel strategies to diagnose, prevent, treat or slow the progression of these devastating disorders.

Full-length TDP-43 (FL TDP-43) consists of 414 amino acids with two RNA recognition motifs (RRM1 (105–169) and RRM2 (193–253)) flanked by an N-terminal domain (NTD (1–80)) and C-terminal domain (CTD (277–414)). The folded domains of RRMs bind specifically to the UG-repeats of RNA and the TG-repeats of DNA and play an important role in TDP-43 function in transcription regulation^9^. The NTD of TDP-43 acquires a novel ubiquitin-like fold when produced as an isolated domain^10^. This NTD forms functional but non-pathological homo-oligomers under physiological conditions and is resistant to cellular stress^11^. The extent to which the structure of the NTD and the mechanism of its assembly in isolation affects the misfolding and aggregation of the FL TDP-43 remains unknown.

The CTD is also known as the low-complexity prion-like domain of TDP-43 and has been implicated in several TDP-43 functions, including regulating its interactions with other proteins^12,13^. It is intrinsically disordered, contains a long stretch of sequences enriched with glutamine/asparagine repeats and glycine residues, which are predicted to be highly amyloidogenic^14,15^, and harbors several mutations associated with familial and sporadic ALS/FTLD-TDP^16,17^. Misfolded and aggregated forms of FL TDP-43 or its C-terminal fragments (CTFs) are commonly found in the brains of ALS/FTLD patients (Supplementary Table 1).

Previous studies showed that not all TDP-43 inclusions in ALS/FTLD-TDP cases possess the classical characteristics of amyloid fibrils^18–22^ (Supplementary Table 1). However, Arseni et al. recently reported the protease-resistant core domain of TDP-43 filaments from patients with ALS-FTLD^23^ exhibited amyloid-like structural features. This work represents a major advance and provides new insight into the structural properties of TDP-43 filaments. However, it does not provide new insight into the positioning of the RNA domains in relation to the core filaments in relation to the organization of the RNA-binding domains and other flanking sequences in the FL TDP-43, or the mechanism of fibril formation by the full-length protein. Therefore, there is still a need for an in vitro system that allows for the generation of full-length TDP-43 fibrils with an amyloid core similar to that observed in the brain-derived TDP-43 fibrils. This is essential to elucidate the molecular mechanisms of TDP-43 aggregation and toxicity and how disease-relevant mutations and posttranslational modifications (PTMs), outside the amyloid core, influence these mechanisms in the context of the FL TDP-43 protein.

In our work, we describe a new method for generating highly ordered filaments derived from native monomeric FL TDP-43 in vitro and devoid of oligomers, amorphous aggregates or monomers. We demonstrate that an amyloid fibril core is buried within these TDP-43 filaments and masked by the flanking ordered domains of TDP-43. Using a limited proteolysis protocol, we were able to unmask the amyloid core, and show that it corresponds to a sequence that is virtually identical to that of the amyloid core of human brain-derived pathological TDP-43 filaments (differs by only three amino acid residues)^23^. Altogether, our in vitro and in-cell-seeding studies suggest a new working model for TDP-43 pathology formation, where post-filament formation PTMs, especially N-terminal cleavages, could play critical roles in regulating TDP-43 fibril uptake, seeding activity and pathology spreading.

## Generation of FL TDP-43 filaments

Most previous studies relied on the use of recombinant TDP-43 purified after refolding from inclusion bodies or TDP-43 fused to non-native solubilizing sequences, which lead to large variations in TDP-43 preparations (Supplementary Table 2). Therefore, we developed expression and purification systems that allowed the production of highly pure FL TDP-43 under native conditions (Supplementary Figs. 1 and 2). This was achieved by expressing FL TDP-43 fused to a removable His_6_-SUMO protein tag (Supplementary Fig. 1a–c), which we and others have shown enhances the solubility of aggregation-prone proteins^24,25^. His_6_-SUMO tag removal by the addition of ubiquitin-like-specific protease 1 (Ulp-1; Supplementary Fig. 1d,e) followed purification by reverse-affinity chromatography resulted in the generation of native FL TDP-43 with high purity (>98%; Supplementary Figs. 1f and 2c–f). The fractions corresponding to predominantly monomeric TDP-43 exhibited high propensity to form oligomers and filaments (Supplementary Figs. 3 and 4).

## FL TDP-43 filaments resemble ALS/FTLD brain filaments

**Fig. 1:**
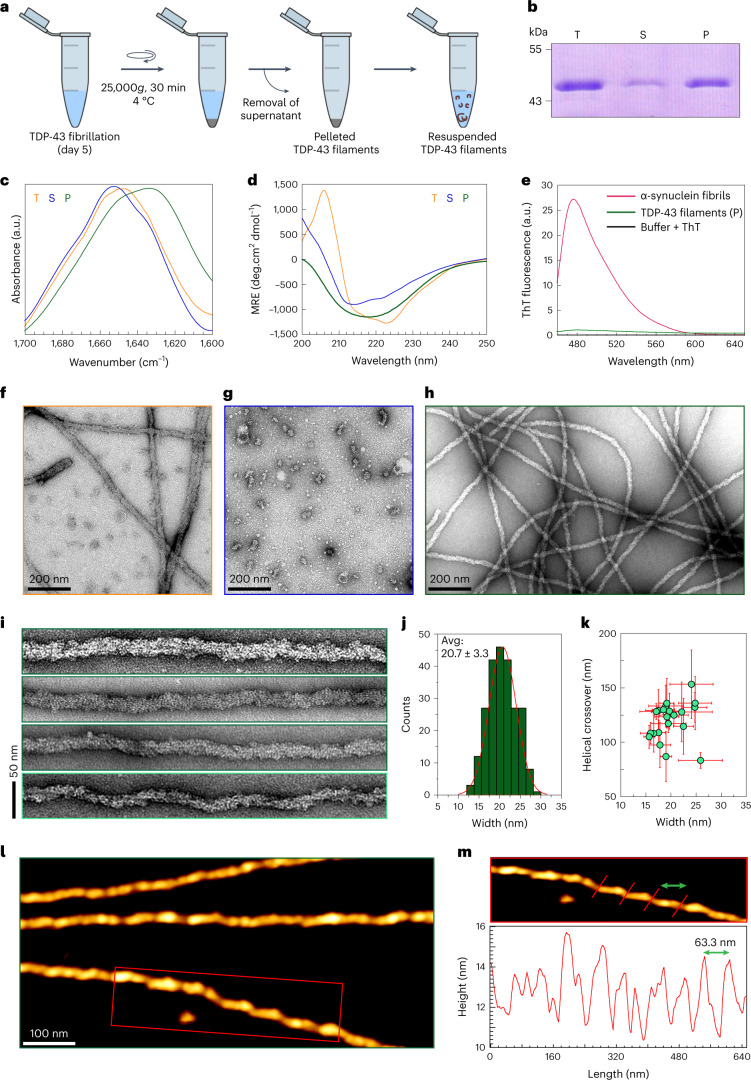
Preparation of FL TDP-43 filaments and structural characterization. **a**, Schematic of the protocol used to isolate TDP-43 filaments from 200-TDP-43. **b**, Coomassie staining of total (T), supernatant (S) and pellet (P) fractions after 5 d of TDP-43 fibrillization. **c**,**d**, Amide I spectra and CD spectra of T, S and P fractions. **e**, ThT fluorescence spectra of resuspended TDP-43 filaments (green), αSyn fibrils (red) or ThT alone (black). **f**–**h**, EM images of T (**f**), S (**g**) and resuspended P fraction (**h**). **i**, EM images of single FL TDP-43 filaments at a magnified view. **j**, Distribution of TDP-43 filaments (*n* = 232) width quantified using the EM images. The red line indicates the Gaussian fit from the width distribution. **k**, Helical crossover of FL TDP-43 filaments plotted against their corresponding widths with error bars. A single green circle represents the mean values obtained for the helical crossover distance and width of one filament. Error bars represent the s.d. (*n* = 21, number of filaments analyzed from three independent experiments). **l**, AFM images of individual TDP-43 filaments. **m**, Magnified AFM image (red box in **l**) showing the helical twists of TDP-43 filaments with rendering red lines. The respective periodicity profile along the fibril axis is shown below with the distance between two peaks marked by a green double-headed arrow. a.u., arbitrary units. Source data

**Extended Data Fig. 1:**
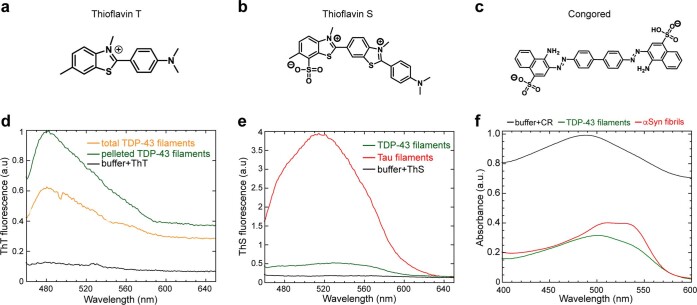
Amyloid dyes bind weakly to FL TDP-43 filaments. **a, b**, and **c**) Chemical structures of ThT (**a**), ThS (**b**), and CR (**c**) molecules. **d**) ThT fluorescence spectra of total TDP-43 filaments after 5 days and resuspended pelleted TDP-43 filaments following isolation protocol. **e**) ThS fluorescence spectra of pelleted TDP-43 filaments and tau fibrils. **f**) CR absorbance spectra of pelleted TDP-43 filaments, αSyn fibrils, and CR in buffer alone. Source data

To determine whether recombinant FL TDP-43 filaments possess amyloid-like properties, we induced FL TDP-43 aggregation (Supplementary Fig. 3) and used centrifugation (Fig. 1a) to isolate filaments free of soluble and oligomeric TDP-43 structures (Fig. 1b–h). Fourier transform infrared spectroscopy and circular dichroism (CD) analysis revealed that resuspended pellets are rich in β-sheet content, which is evident from the amide I maximum at 1,633 cm^−1^ (Fig. 1c), and broader single minima close to 218 nm, respectively (Fig. 1d). FL TDP-43 filaments exhibited surfaces with granular appearance (Fig. 1i) and very weak binding to the classical amyloid binding dyes such as thioflavin-T (ThT), thioflavin-S (ThS) and Congo red (CR; Fig. 1e and Extended Data Fig. 1), in comparison to α-synuclein (αSyn) and tau fibrils, which were used as positive controls and exhibited significant increases in ThT/ThS signal (Fig. 1e and Extended Data Fig. 1d,e)^26,27^ and induced the expected red shift in the CR absorption maxima (from 490 nm to 502 nm; Extended Data Fig. 1f).

**Extended Data Fig. 2:**
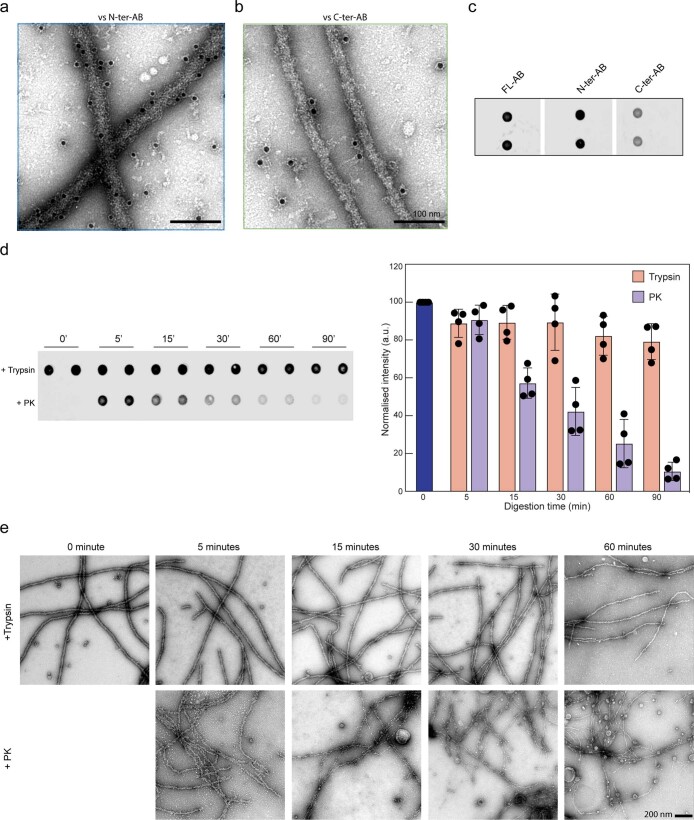
Proteinase K efficiently exposes the C-terminal amyloid core of TDP-43 filaments. **a** and **b**) EM images of TDP-43 filaments immunogold labeled with (**a**) N-terminal (N-ter-AB) and (**b**) C-terminal (C-ter-AB) specific TDP-43 antibodies. **c**) Dot blot of FL TDP-43 filaments stained with full-length (FL-AB), N-ter-AB, and C-ter-AB specific TDP-43 antibodies. **d**) Dot blot analysis of trypsin (T) and proteinase K (PK) treated TDP-43 filaments at different time points stained using N-terminal specific TDP-43 antibody. Bar graph shows the quantified antibodies staining intensities of the respective dot blot images. Error bars represent SD, n = 4 independent experiments. **e**) Representative electron micrographs of trypsin (top row) and PK (bottom row) treated TDP-43 filaments at different time points. Source data

Electron microscopy (EM) studies showed that the FL TDP-43 filaments are unbranched and several µm-long with a mean diameter of 20.7 nm (±3.3 nm) that bear morphological similarity to the TDP-43 filaments isolated from patient sources (Fig. 1i,k and Supplementary Table 1). FL TDP-43 filaments are much wider in width (Fig. 1h–k) than amyloid fibrils formed by other amyloid proteins such as Aβ, αSyn, tau, and exon1 of the huntingtin proteins (Httex1)^28–32^. Closer examination of the same samples using atomic force microscopy (AFM) showed that some fibril populations possess apparent periodicity with varying helical twists appearing to have left-handed helical twists (Fig. 1l,m). The surface of the filaments suggests that they are decorated by the globular domains of TDP-43 monomers (Extended Data Fig. 2a–c).

## TDP-43 filaments possess an amyloid core with a fuzzy coat

**Fig. 2:**
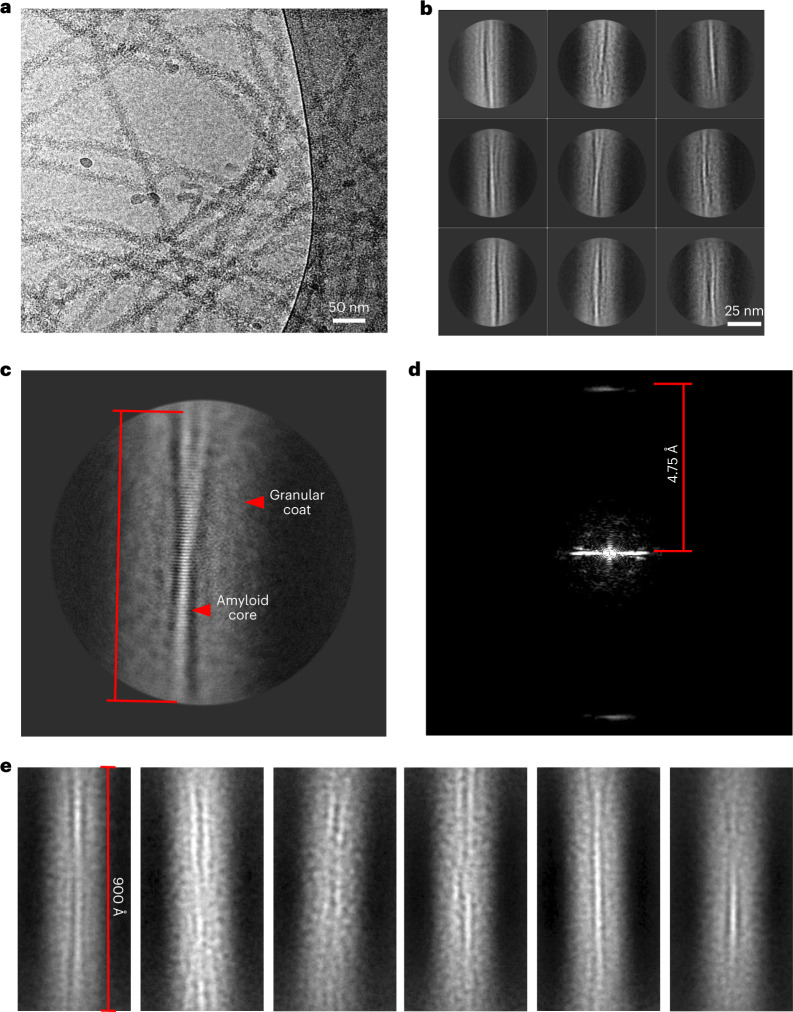
Cryo-electron microscopy of FL TDP-43 filaments unveils an amyloid core flanked by a fuzzy coat. **a**, Cryo-EM micrograph of FL TDP-43 filaments. **b**, Representative 2D class-average images (*n* = 186,752) extracted segments from cryo-EM micrographs of FL TDP-43 filaments with clear separation of β-strands. **c**, Representative 2D class-average images with a visible crossover. **d**, Fourier amplitudes of the 2D class-average image from **c**; measured helical rise was 4.75 Å. **e**, Representative 2D class-average images of segments (~900 Å) extracted with a large 1,000-Å box size and binned four times.

Our observations led us to propose a model whereby TDP-43 aggregation, driven by the CTD, results in the formation of amyloid filaments that are heavily decorated by the structured domain of the protein (NTD and RRMs), which mask the amyloid core of the filaments, thus rendering the amyloid core inaccessible to amyloid-specific dyes such as ThS/ThT and CR. To test our hypothesis, we analyzed these filaments with cryo-EM (Fig. 2a). The cryo-EM analysis revealed long fibrillar structures with a granular appearance on their surfaces. Reference-free two-dimensional (2D) classification of FL TDP-43 helical segments revealed a ~40–70-Å thin amyloid core composed of β-strands stacked at a 4.75-Å distance along the fibril axis (Fig. 2b,d) and surrounded by a 200–250-Å granular coat (Fig. 2c). Reference-free 2D classification of helical segments extracted with large box size (1,000 Å) and binned four times (Fig. 2e) showed that the thin amyloid core is still visible along the 900-Å filament axis but without visible crossover. These observations demonstrate the presence of an amyloid core buried in the FL TDP-43 filaments. This explains the apparent periodicity of the TDP-43 filaments seen by AFM (Fig. 1l,m).

## Proteinase K unveils the amyloid core of TDP-43 filaments

**Fig. 3:**
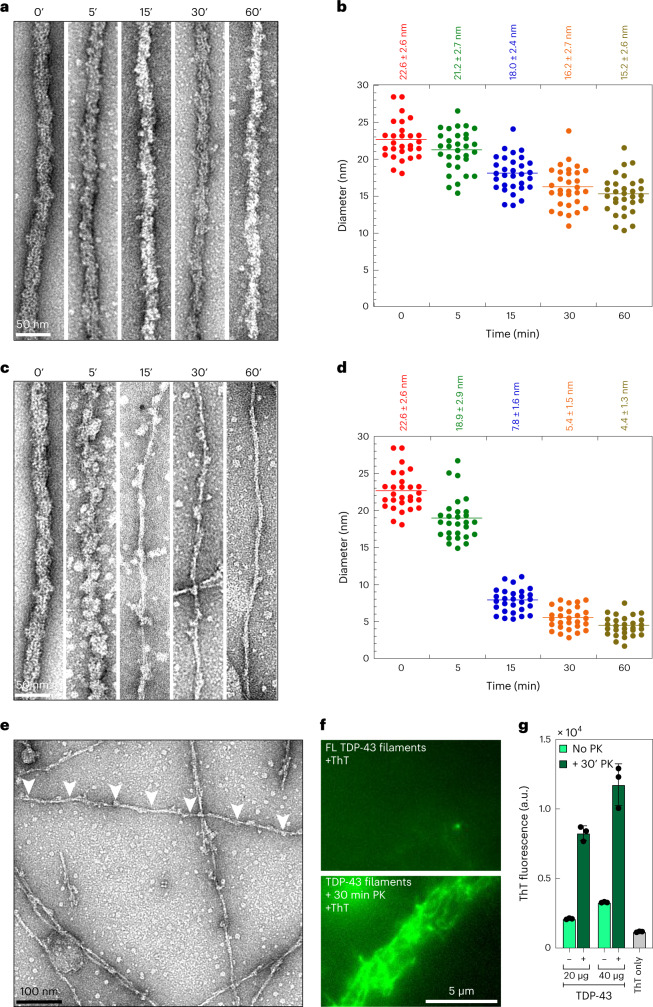
Limited proteolysis of FL TDP-43 filaments exposed the amyloid core through the thinning of fibril diameter. **a**,**b**, Representative EM images of trypsin-treated TDP-43 filaments at different time points (**a**) and dot plots representing their respective diameters (**b**). **c**,**d**, Representative EM images of PK-treated TDP-43 filaments at different time points (**c**) and plots representing their respective diameters (**d**). For **b** and **d**, each dot represents the diameter of a single filament with *n* = 28–31 filaments analyzed from each time point over three independent experiments. **e**, EM image of 30-min PK-treated TDP-43 filaments showing the twists and periodicity from the amyloid core (white arrowheads). **f**, TIRFM images of ThT signal of FL TDP-43 filaments with no PK (top) and after PK treatment for 30 min (bottom). **g**, Quantification of ThT fluorescence before and after treatment of FL TDP-43 filaments at the normalized concentration. Data represent the mean ± s.d., *n* = 3 from a minimum of three independent experiments. Source data

**Extended Data Fig. 3:**
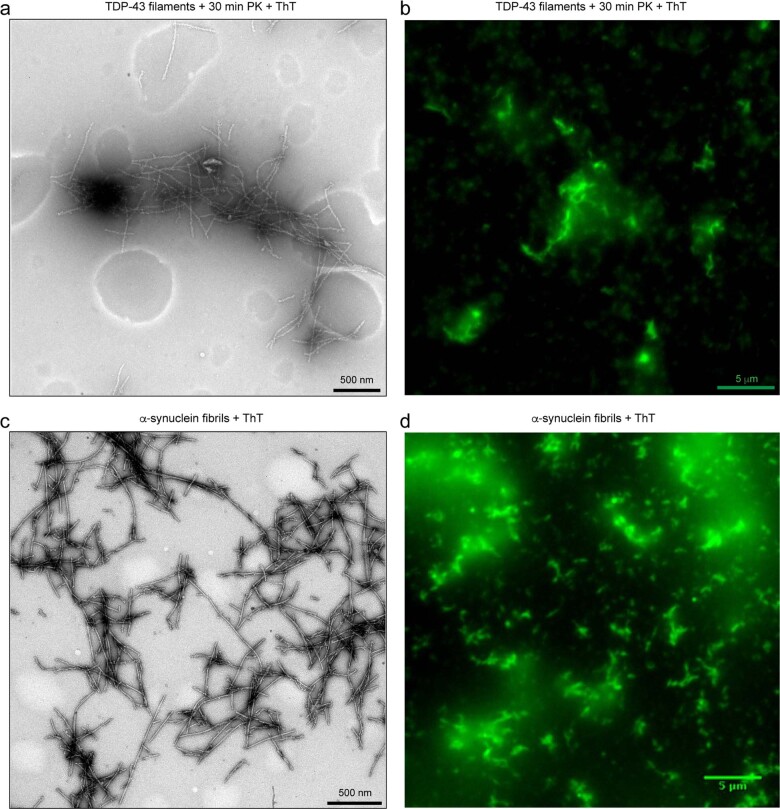
Proteinase K treatment enhances ThT binding to TDP-43 filaments. **a**) EM micrograph of PK-treated TDP-43 filaments. **b**) TIRFM image of PK-treated TDP-43 filaments added with ThT. **c**) EM micrograph of α-synuclein fibrils. **d**) TIRFM image of α-synuclein fibrils added with ThT.

Next, we developed a method to unmask the amyloid core of the FL TDP-43 filaments by removing the globular domains that decorate the TDP-43 filaments by limited proteolysis followed by EM analysis. Treatment with trypsin resulted in a reduction of their diameter from 22 to 15 nm after 60 min (Fig. 3a,b). However, the surfaces of the treated filaments remained covered by a granular surface, reflecting the presence of trypsin-resistant globular domains (Fig. 3a). Next, we treated TDP-43 filaments with proteinase K (PK), which has broader specificity. After 30 min of PK treatment, we observed a major reduction in filament diameter from ~22.6 to ~5 nm (Fig. 3c,d) that exhibited a regular periodicity with twists and turns along the fibrillar axis (Fig. 3e, arrowheads). Unlike the filaments derived from FL TDP-43, the amyloid dye ThT binds to the PK-resistant fibril core and its binding could be directly visualized using total internal reflection fluorescence microscopy (TIRFM; Fig. 3f and Extended Data Fig. 3a–d). Quantitative assessment of ThT binding to an equal amount of PK-treated and untreated filaments showed a fivefold increase in ThT fluorescence for the PK-treated samples (Fig. 3g). Together, these results confirm our hypothesis that the FL TDP-43 filaments possess an amyloid core, which is buried but can become exposed upon cleavage of the flanking structured domains.

## Identification of TDP-43 amyloid core sequence

**Fig. 4:**
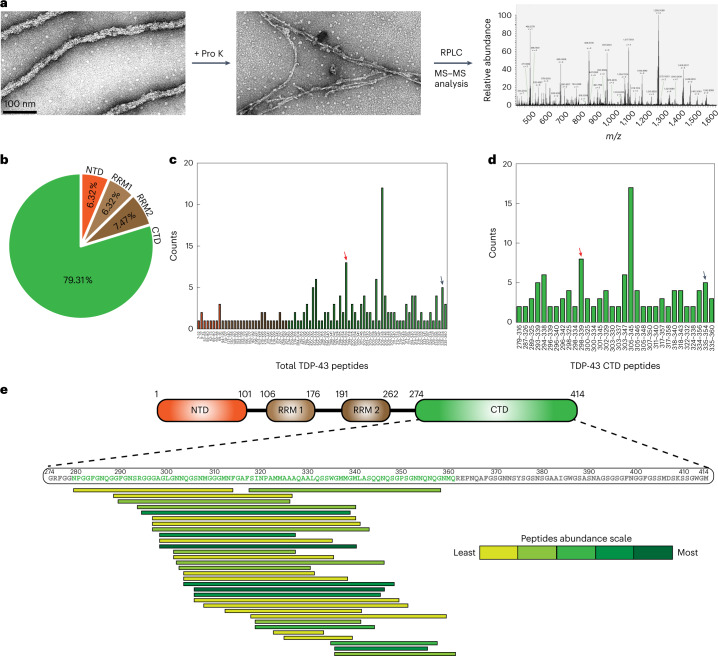
LC–MS/MS of proteinase K-unmasked TDP-43 filaments. **a**, TDP-43 filaments before and after PK treatment identifying the stable fibrillar core and its following LC–MS/MS analysis. **b**, Pie chart showing the percentage of identified peptides covering the whole region of TDP-43. **c**,**d**, Bar diagrams showing the sequence numbering of peptides identified from MS–MS analysis and their repeat counts for total peptides (**c**) and peptides from the CTD of TDP-43 (**d**). The red and black arrows show the highly abundant peptides P1 (298–339) and P2 (335–354), respectively. **e**, Sequence and repeat details of CTD peptides showing the region of amino acids 279–360 (green) as the fibrillar core of TDP-43 filaments. RPLC, reversed-phase liquid chromatography. Source data

To more precisely map out the peptide region forming the amyloid core, we analyzed the digested TDP-43 filaments after PK treatment using liquid chromatography–tandem mass spectrometry (LC–MS/MS; Fig. 4a). This resulted in the identification of several peaks corresponding to TDP-43 peptides with masses between 900 Da and 5,000 Da (Supplementary Fig. 5). We found 174 shorter peptides (ranging from 9 to 45 amino acids) covering the entire TDP-43 sequence (Fig. 4b,c) with sequence coverage of ~75% (Supplementary Fig. 5). Interestingly, close to 80% of the peptides identified were from the CTD of TDP-43 (Fig. 4b,c and Supplementary Fig. 5), in contrast to only 8% in samples of monomeric TDP-43 treated with PK (Supplementary Fig. 6). Particularly, peptides P1 (298–339) and P2 (335–354) were highly enriched (Fig. 4d). Based on the abundance of peptides with respect to the spectral counts containing at least two repeats (Fig. 4d), we identified that the 82 amino acids containing core peptide (CP) regions between 279 and 360 likely formed the amyloid core of TDP-43 (Fig. 4e). Surprisingly, the sequence of the amyloid CP is almost identical to the sequence (281–360) determined from brain-derived pathological TDP-43 protein from individuals with a clinical history of ALS with FTLD^23^. Consistent with these findings, immunogold labeling followed by EM analysis showed that only antibodies that recognize the epitopes present on the NTD but not on the CTD exhibited extensive decoration of gold particles on the surface of TDP-43 filaments (Extended Data Fig. 2a,b). Similar results were obtained via dot blot analysis of the same samples and following the PK or trypsin treatment (Extended Data Fig. 2c–e).

## Cryo-electron microscopy structure of the core peptide (279–360) fibrils

**Fig. 5:**
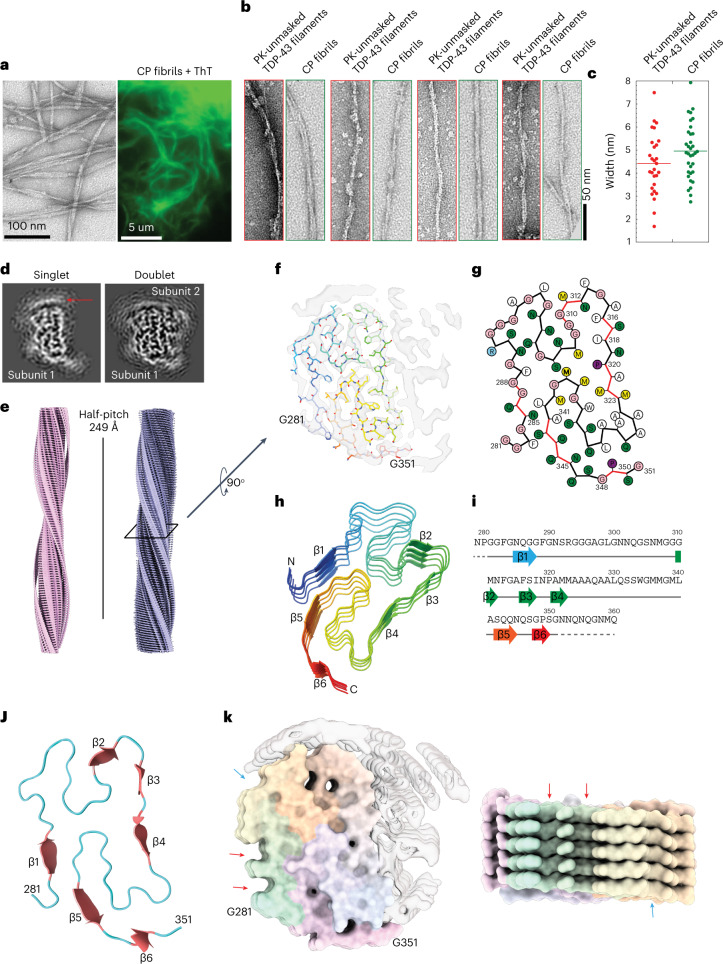
Morphological properties and cryo-electron microscopy structure of the core peptide 279–360 fibrils. **a**, EM image and wide-angle fluorescence microscopic image of ThT-positive CP fibrils. Images represent the data from at least three independent repeats. **b**,**c**, EM images showing morphological similarities between PK-unmasked TDP-43 filaments and CP fibrils (**b**) and width distribution (**c**). **d**, Central slice of 3D reconstructions of two major populations (singlet and doublet) of CP fibrils. Disconnected density from the second protofilament on top of the singlet is highlighted with a red arrow. **e**, Cryo-EM 3D reconstructions of singlet and doublet CP fibrils with a half-pitch of 249 Å. **f**, Central slice of 3D reconstruction (transparent, EMDB-13795) with one layer of an atomic model (G281-G351, PDB 7Q3U). **g**, Scheme of one cross-sectional layer of the CP atomic model. **h**, Tilted view of five neighboring subunits of CP filament. Six β-strands of the top subunit are shown. **i**, Schematic of the sequence and secondary structure of the CP fibrils. Six β-strands are represented by arrows colored from blue to red, loops/turns represented by a gray line, and the unsolved residues as the dashed lines. **j**, One cross-sectional layer of the atomic model revealing the ‘H-fold’ conformation of the CP fibrils. Six β-strands are shown as thicker red arrows with the loops in blue. **k**, Surface representation of CP fibrils with the arrows pointing to the shallow grooves on the sides of the CP fibril structure. Source data

**Extended Data Fig. 4:**
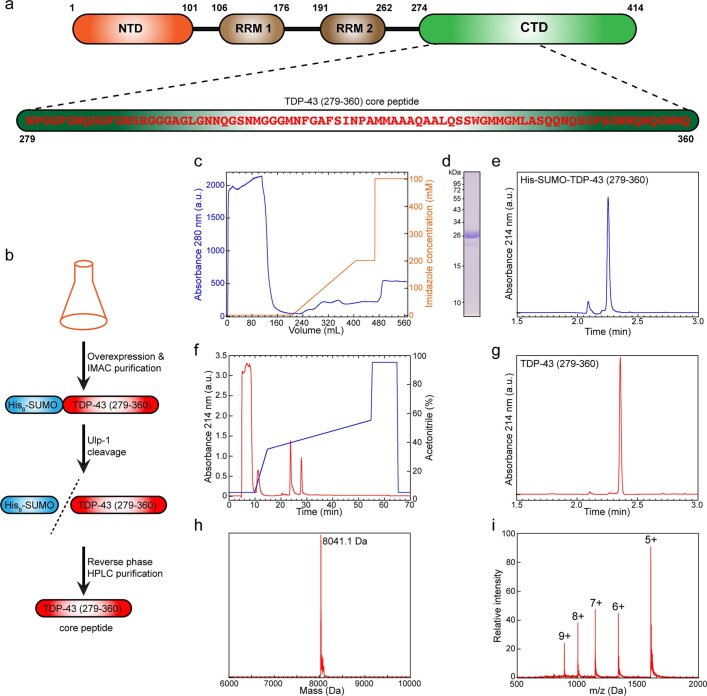
Purification and characterization of the amyloid core peptide 279–360. **a**) Schematic depiction highlighting the different domains of FL TDP-43 and amino acid sequence of TDP-43 (279–360) core peptide (CP) region. **b**) Adaption of the SUMO-based strategy for the recombinant expression and purification of CP. **c**) IMAC chromatogram of His_6_-SUMO-TDP-43 (279–360) CP purification. **d**) SDS-PAGE analysis of His_6_-SUMO-TDP-43 (279–360) CP. **e**) UPLC of His_6_-SUMO-TDP-43 (279–360) CP. **f**) Reverse phase HPLC chromatogram of His_6_-SUMO-TDP-43 (279–360) CP after ULP-1 treatment. **g**) UPLC of TDP-43 (279–360) CP. **h** and **i**) ESI-MS analysis of TDP-43 (279-360) CP. The theoretical molecular weight of TDP-43 (279-36) CP is 8041.7 Da. Source data

Next, we investigated if the CP (279–360) has the propensity to form fibrils on its own. For this, we expressed and purified recombinant CP following the same strategy described for FL TDP-43 protein (Extended Data Fig. 4). Under the same in vitro aggregation conditions used for the FL TDP-43, CP formed ThT-positive β-sheet-rich fibrils of different morphologies (Fig. 5a). An initial comparison of EM structures of fibrils formed by the CP and the amyloid core from the PK-unmasked TDP-43 filaments revealed similar features; (1) the morphology of the CP fibrils (Fig. 5b); and (2) an average diameter of 5 nm, similar to that of the unmasked fibrils, ~4.5 nm (Fig. 5c). These similarities were not observed in fibrils generated with other shorter and abundant peptides identified by the LC–MS/MS (that is, P1 (298–339) and P2 (335–354); Supplementary Fig. 7).

**Extended Data Fig. 5:**
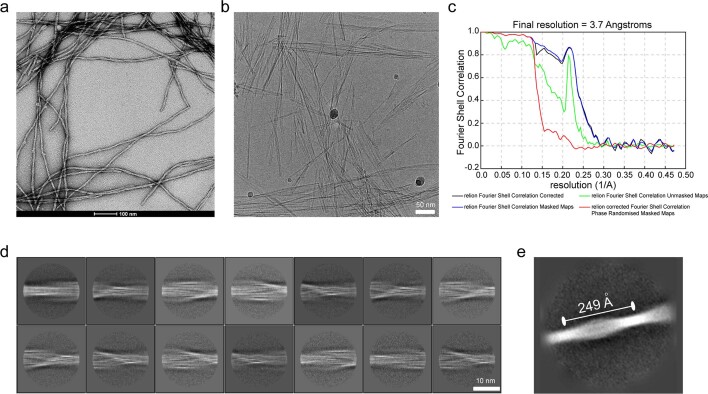
Cryo-EM analysis of CP fibrils. **a**) Representative TEM image (left) and **b**) raw cryo-EM image (right) of fibrils from CP (scale bars: for TEM is 100 nm and for cryo-EM is 50 nm). **c**) Fourier shell correlation curves from RELION between the two independently refined half-maps. **d**-**e**) Representative 2D class average images of CP segments (**d**) and large extracted CP segment (**e**) with visible helical crossover and corresponding half-pitch distance of 249 Å.

Next, we determined the structure of CP fibrils by cryo-EM. Consistent with the negative-stain EM data, the cryo-EM analysis showed polymorphic CP fibrils exhibiting variable length as well as a tendency to undergo lateral associations (Extended Data Fig. 5a,b). Several rounds of reference-free 2D classification of the CP fibril images revealed averages with a thin ~80 Å bright density corresponding to the amyloid core with a clear 4.83-Å separation of β-strands. The distance between two consecutive crossovers was measured from the 2D class-average image of a large helical segment (1,024 pixels), resulting in a 249-Å half-pitch distance and corresponding −3.09° helical twist (Extended Data Fig. 5d,e). A combination of three-dimensional (3D) classification and structural refinement revealed two populations of ~70-Å CP fibrils (Fig. 5d). Both populations were present in similar proportions and shared similar helical parameters. The first CP fibril population was reconstructed to 4.1-Å resolution (Fig. 5d,e) and was composed of a single CP protofilament with stacked subunits along the fibril axis with a helical rise of 4.86 Å and a helical twist of −3.12°. An extra density disconnected from the main chain was detected, most likely representing an ordered part of the second protofilament (Fig. 5d, red arrow). The second CP fibril population was reconstructed to 3.7-Å resolution (Extended Data Fig. 5c) and revealed two protofilaments, which were non-identical with stacked subunits along the fibril axis with a helical rise of 4.83 Å and a helical twist of −3.09° (Fig. 5d,e). The atomic model was built from residues Gly281 to Gly351 into the most resolved density corresponding to the CP fibrils of one protofilament (Fig. 5f). The density of the second protofilament was mostly disordered, with disconnected loops, and was not used for model building. One cross-sectional layer of the atomic model revealed a novel ‘H-fold’ conformation of the CP filaments that consists of six linked β-strands with rigid loops and turns. (Fig. 5g–j). The surface properties of the CP fibrils possessed a rugged nature on the ends but with prominent grooves on the sides (Fig. 5k). The cryo-EM reconstruction of the CP fibril was sufficient to recognize that the protofilament had an H-shaped fold and enabled us to propose a structural model. Further studies are required to obtain a higher-resolution 3D reconstruction of the CP fibril to facilitate an accurate model building.

## Proteinase K-exposed TDP-43 filaments have higher seeding activity

**Fig. 6:**
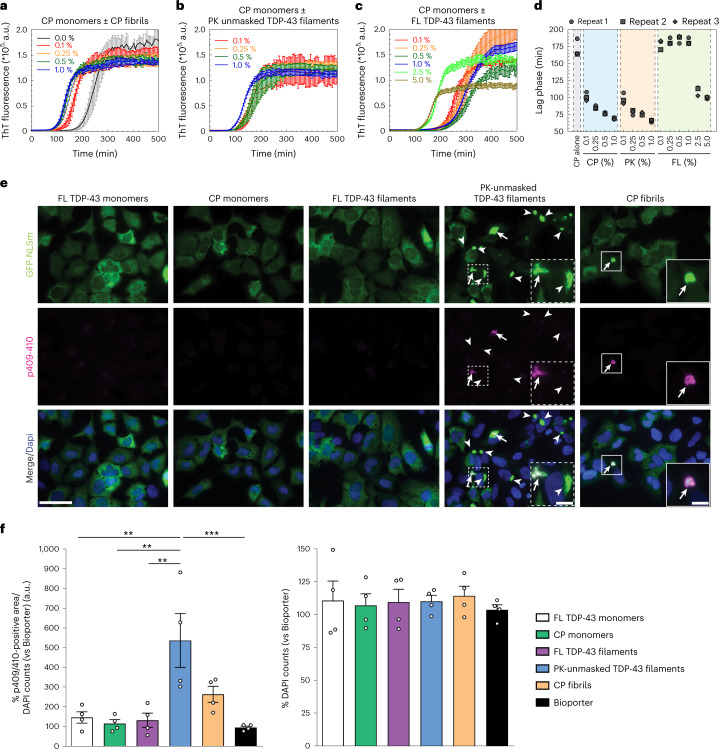
Unmasking the core of FL TDP-43 filaments enhances their uptake and seeding activity. **a**–**c**, Aggregation kinetics of CP monomers monitored by ThT fluorescence in the absence and presence of varying concentrations of CP fibrils (**a**), PK-unmasked TDP-43 filaments (**b**) and FL TDP-43 filaments (**c**) used as seeds. Averages of three traces are shown as solid lines in all the samples. All figures show ThT intensity as a function of time (non-normalized raw data). **d**, Measurement of the lag phase of all the samples from **a**–**c**. Data are shown as the mean ± s.d., *n* = 3 independent experiments. **e**, Representative fluorescence images of ICC analysis using the p409–410 antibody (red and merge) in GFP-NLSm-expressing cells (green and merge) transduced with FL TDP-43, CP monomers, FL TDP filaments, PK-unmasked TDP-43 filaments and CP fibrils, at 3 d.p.t. Arrows point to p409–410-positive GFP-NLSm aggregates; arrowheads point to non-phosphorylated GFP-NLS aggregates. Cells were counterstained with DAPI to label the nuclei. Scale bars, 50 µm and 10 µm (insets). **f**, Plots show the quantification of the percentage area occupied by p409–410 staining (left plot) at 3 d.p.t. measured in **e** and the number of DAPI counts (percentage; right plot). Bar plots show the mean, and whiskers are the s.e.m., with individual points representing a different experimental replica (*n* = 4). A one-way analysis of variance (ANOVA) followed by Tukey’s multiple-comparisons test was used for the analysis; left plot, *P* = 0.0006, *F*(5,18) = 7.433. **P < 0.01, ***P < 0.001. Source data

**Extended Data Fig. 6:**
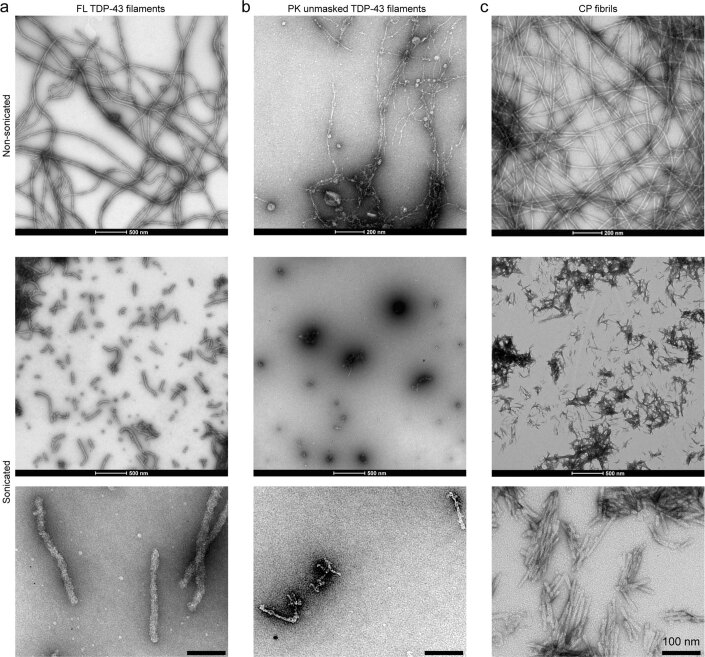
Fibril preparations for assessing TDP-43 and core peptide seeding. **a-c**) Representative electron micrographs of non-sonicated and sonicated FL TDP-43 filaments (**a**) PK-unmasked TDP-43 filaments (**b**) and CP fibrils (**c**).

The presence of FL TDP-43 and CTFs in pathological aggregates in the brains of individuals with ALS and FTLD-TDP is well documented in the literature^2,4,23^. This led us to hypothesize that post-aggregation proteolytic cleavage may be required to unmask the buried amyloid core in FL TDP-43 filaments, which could facilitate secondary nucleation and seeding and aggregation of non-pathological TDP-43 protein in the brain. To test this hypothesis, we investigated whether the seeding activity of FL TDP-43 filaments is enhanced by pretreatment with PK. Because the FL TDP-43 filaments do not bind the amyloid-specific dyes (ThT/ThS or CR), we used CP monomers as a substrate to assess the seeding activity of the amyloid core using the ThT assay (Supplementary Fig. 8). We compared the seeding activity of sonicated FL TDP-43 filaments, PK-unmasked TDP-43 filaments and CP fibrils (Extended Data Fig. 6) at increasing concentrations of seeds. Even the lowest concentration of the CP and PK seeds (0.1% (wt/vol)) resulted in a significant decrease in the lag phase (~100 min) compared to the unseeded sample (~175 min). The PK-unmasked TDP-43 filaments showed significantly higher seeding activity than the FL TDP-43 filaments at all seeding concentrations tested (0.1%, 0.25%, 0.5% and 1% (wt/vol); Fig. 6a–d). Interestingly, the seeding with CP fibrils or PK-unmasked TDP-43 filaments resulted in virtually identical aggregation profiles and lag phases (Fig. 6a,b,d). In contrast, FL TDP-43 filaments seeding was observed only at a concentration of seeds 25 times higher than that used with the CP fibrils (Fig. 6c,d). This might be because FL TDP-43 filament ends, presumably, retain some seeding activity, as observed by EM (Supplementary Fig. 9, white arrowheads).

## TDP-43 filament cleavage is essential for seeding in cells

Next, we sought to determine whether FL TDP-43 filaments, PK-unmasked TDP-43 filaments and CP fibrils were capable of seeding and inducing de novo TDP-43 aggregation in cells. We used a doxycycline-inducible QBI-293 stable cell line expressing mislocalized cytoplasmic TDP-43 (iGFP-NLSm) due to the inclusion of mutations within the bipartite nuclear localization signal^33^. iGFP-NLSm cells were transduced with FL TDP-43 monomers and filaments, PK-unmasked TDP-43 filaments or CP monomers and fibrils. The formation of phosphorylated TDP-43 aggregates was assessed 3 days post-transduction (d.p.t.) by immunocytochemistry (ICC) using the p409–410 antibody (Fig. 6e). The transduction of FL TDP-43 monomers, CP monomers and FL TDP-43 filaments did not result in a significant formation of de novo TDP-43 aggregates (Fig. 6e). However, we detected a large number of GFP-NLSm and phospho-positive aggregates in PK-unmasked TDP-43 filament-treated cells and, to a lesser extent, in cells transduced with CP fibrils (Fig. 6e, arrows). Notice that not all the GFP-NLSm aggregates were positive for p409–410 in PK-unmasked treated cells (Fig. 6e, arrowheads). Further quantification of the percentage of area occupied by p409–410 immunostaining showed a fivefold increase as compared to either FL TDP-43 monomers and filaments or Bioporter (transduction reagent). The increase in the burden of p409–410-positive aggregates in cells transduced with CP fibrils showed a trend toward significance (Fig. 6f). No significant differences in cell viability were found at 3 d.p.t. when comparing iGFP-NLSm cells transduced with the different samples (Fig. 6f, DAPI counts). As we observed very minimal p409–410 immunostaining for FL TDP-43 filaments compared to PK-unmasked and CP fibrils, we examined the uptake efficiency of these different seeds after fluorescence labeling (Supplementary Fig. 10a–c). Supplementary Fig. 10d shows that the PK-unmasked and CP fibrils were internalized more readily and in higher amounts compared to FL TDP-43 filaments.

**Extended Data Fig. 7:**
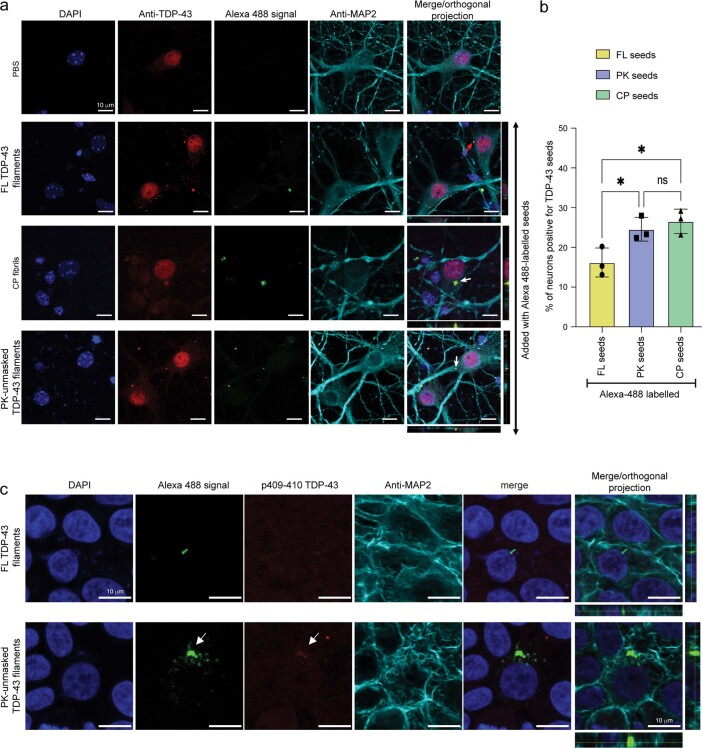
Unmasking the amyloid core is essential for seeding in neurons. **a**) Mouse primary cortical neurons treated with PBS, Alexa 488-labeled FL TDP-43 filaments, Alexa 488-labeled CP fibrils, and Alexa 488-labeled PK-unmasked TDP-43 filaments for 3 days and analyzed by ICC using an anti-full-length TDP-43 antibody. From left to right: DAPI staining showing the nucleus (blue), staining using anti- TDP-43 antibody (red), the fluorescence signal from Alexa 488 labeled fibrils (green), and staining with anti-MAP2 antibody identifying neurons (red), and orthogonal projections. **b**) Quantification of Alexa-488 labeled TDP-43 filaments/fibrils uptake in mouse primary cortical neurons. Bar plots show mean and whiskers s.e.m., with individual points representing a different experimental replica (n = 3). A one-way ANOVA followed by Tukey’s multiple comparisons test was used for the analysis; ∗P < 0.05. ns: non-significant. The statistics results and the exact p-value are provided in the source data. **c**) Mouse primary neurons after 3 days of treatment with Alexa 488-labeled FL TDP-43 filaments and PK-unmasked TDP-43 filaments and analyzed by ICC using an anti-phospho TDP-43 antibody (409/410). From left to right: DAPI staining showing the nucleus (blue), the fluorescence signal from Alexa 488 labeled fibrils (green), staining using anti-phospho TDP-43 antibody (red), and staining with anti-MAP2 antibody identifying neurons (red), and orthogonal projections. Source data

In mouse primary neurons transduction with fluorescently labeled CP fibrils and PK-unmasked TDP-43 filaments, we detected the aggregation of endogenous TDP-43 protein (Extended Data Fig. 7a), using antibodies against full-length and phosphorylated TDP-43, co-localizing with the exogenously added Alexa Fluor 488-labeled PK-unmasked TDP-43 filaments (Extended Data Fig. 7a,c, white arrows). Although the FL TDP-43 filaments were not readily uptaken by neurons, compared to the unmasked filaments or CP fibrils (Extended Data Fig. 7b), we observed no seeding in cells that internalized FL TDP-43 filaments (Extended Data Fig. 7a,c), similar to what we observed with the QBI-293 cells (Fig. 6e,f and Supplementary Fig. 10d). These results further support our hypothesis that unmasking the amyloid core of TDP-43 filaments may be necessary to induce more efficiently the uptake and/or seeding and aggregation of soluble TDP-43 protein.

## Amyloid core exposure boosts seeding of brain-derived TDP-43

**Fig. 7:**
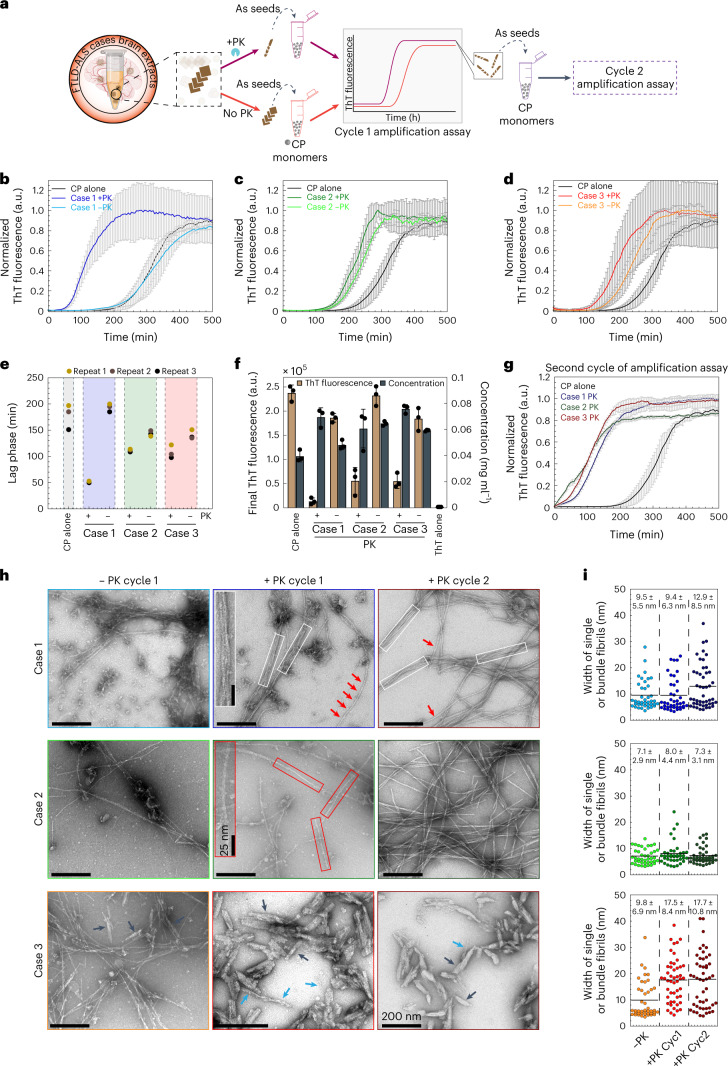
Exposure of the amyloid core boosts seeding of brain-derived TDP-43. **a**, A schematic depiction of the workflow of the aggregation kinetics of CP monomer seeding with brain-derived TDP-43 extracts treated with or without the PK treatment. **b**–**d**, ThT fluorescence-based first cycle seeding assay of CP monomers in the absence or presence of PK-untreated (−PK) or treated (+PK) brain extracts from three cases (1–3). Solid lines represent means from three independent repeats and error bars show the s.d. **e**, Dot plot showing the lag phase of aggregation curves estimated from **b**–**d**. **f**, Bar graph displays the mean of final ThT fluorescence and concentration of protein sedimented at the kinetic end time point of all the samples from **b**–**d**. The graph represents the mean ± s.d. of three independent experiments. **g**, The second cycle of ThT-based fluorescence seeding assay of CP monomers re-seeded with end time point aggregates from (+PK) brain extracts of all three cases (1–3) from **b**–**d**. Solid lines represent means from three independent repeats and error bars indicate the s.d. **h**, EM images from the endpoint of the first cycle (**a**–**c**) and second cycle (**g**) aggregation assays of samples seeded with PK-untreated (−PK) and PK-treated (+PK) brain extracts from the first cycle seeding assay (−PK and +PK cycle 1) and also from the second cycle seeding assay (+PK cycle 2). White rectangular boxes display the fibrils appearing as bundles. Red and blue arrows show the single fibrils, and black arrows present the short and sturdy fibrillar morphologies. **i**, Dot plots showing the distribution and average of the width of single and bundled fibrils measured from all the images from **f**. Each dot represents the diameter of a single filament with *n* = 40–48 numbers of filaments analyzed for each sample from **h** over three independent experiments. Source data

**Extended Data Fig. 8:**
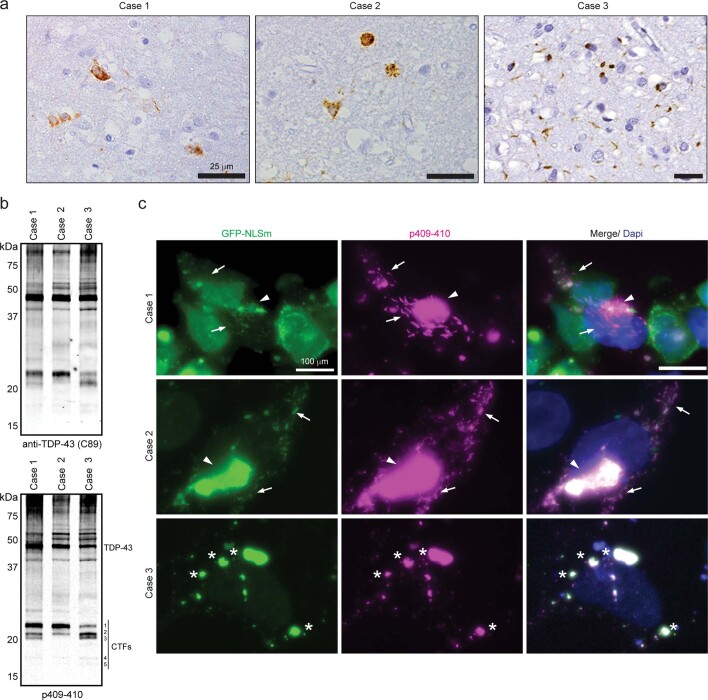
Characterization of the three ALS/TFLD-TDP cases used in this study. **a**) Representative photomicrographs of p409-410 immunostaining in frontal cortex sections of three different ALS/FTLD-TDP cases used in the study. Scale bar=50 µm. **b**) Immunoblot analysis of the sarkosyl-insoluble fractions obtained from the frontal cortex using a pan-TDP43 antibody (C89) and a phosphorylation-specific TDP-43 specific antibody (p409-410). Molecular weight markers in kDa are shown on the left and the position of full-length TDP-43 protein (TDP-43) and C-terminal fragments CTFs (bands #1, #2, #3, #4, and #5) are shown on the right. **c**) Representative ICC images of GFP-NLSm expressing cells (green and merge) transduced with sarkosyl-insoluble brain extracts analyzed in **b**) (that is, cases 1, 2, and 3) at 3 days post-transduction showing 1% Triton-insoluble phospho-TDP-43-positive cytoplasmic aggregates (p409-410, magenta, and merge). In case 1 and case 2, transduced cells’ arrows point to small linear, straight, and wavy aggregates, and the arrowhead points to bigger heterogeneous filamentous aggregates. On the other hand, asterisk labels in cells transduced with the sarkosyl-insoluble brain (from case 3) reveal the formation of round and spherical phosphorylated aggregates. Cells were counterstained with DAPI to label the nuclei (blue). Scale bar=20 µm. All experiments were repeated at least 3 times.

**Extended Data Fig. 9:**
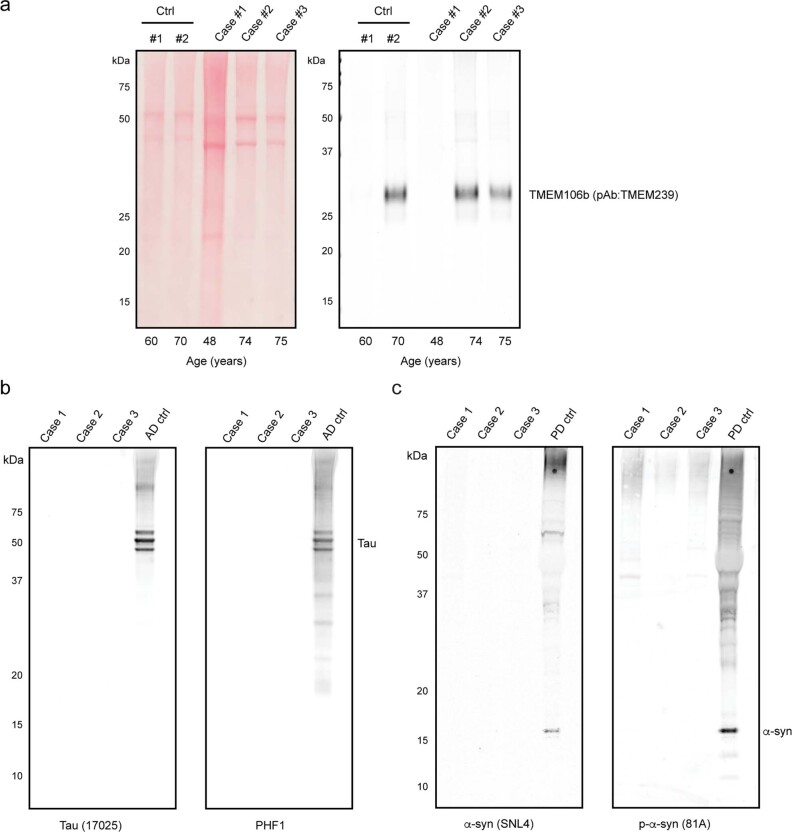
Presence of TMEM106b fragments in controls and individuals > 60 years. **a**) Ponceau S staining (left) and immunoblotting (right) of sarkosyl-insoluble brain extracts (2 neurologically normal controls and 3 ALS/FTLD-TDP cases). Immunoblotting was done using rabbit polyclonal antibody specific for TMEM239. **b-c**) ALS/FTLD-TDP patients’ brain-derived sarkosyl-insoluble fractions analyzed in A were immunoblotted using antibodies specific for total tau (17025) and hyper-phosphorylated pathological tau (PHF-1) and specific for total α-synuclein (SNL4) or phosphorylated α-syn (81 A). Enriched tau preparations from an AD brain (AD ctrl) or α-synuclein enriched from a PD brain (PD ctrl) were used as positive controls.

Next, we investigated whether exposure of the amyloid core is necessary to reveal or enhance the seeding activity of human brain-derived TDP-43 pathogenic seeds (Fig. 7a). We used sarkosyl-insoluble TDP-43 protein extract from three different ALS/FTLD-TDP cases that presented distinctive morphological phospho-positive TDP-43 aggregates in the frontal cortex and differential TDP-43 band patterns specifically related to the CTFs (Extended Data Fig. 8a,b and Supplementary Table 3). The content of TDP-43 protein was measured by enzyme-linked immunosorbent assay (Supplementary Table 4), and an equivalent amount of sarkosyl-insoluble TDP-43 protein was used as pathogenic seeds to test their biological seeding activity inducing de novo TDP-43 aggregates using the iGFP-NLSm cell-based system^33,34^. Whereas brain-derived seeds from cases 1 and 2 induced the formation of filamentous structures (Extended Data Fig. 8c, arrows, and arrowheads), seeds from case 3 induced the formation of more round and compact aggregates (Extended Data Fig. 8d, asterisks). Interestingly, immunogold labeling of the sarkosyl-insoluble TDP-43 extracts from the three ALS/FTLD-TDP cases revealed the presence of filamentous structures, which were all positive for TDP-43 (Supplementary Fig. 11). Several groups have recently reported the presence of TMEM106B in brain samples from individuals with FTLD-TDP and other NDs^35–38^. Therefore, we also assessed the presence of CTFs of TMEM106B protein in our participant brain-derived extracts and aged-human brains (healthy controls) by immunoblot using TMEM106B-specific antibody. As shown in Extended Data Fig. 9a and consistent with the previous report^35^, TMEM106B fragments were only present in samples from individuals >60 years old (cases 2 and 3 and healthy control >60 years), whereas TDP-43 filaments were detected by immunogold labeling in all participant cases (Extended Data Fig. 9a and Supplementary Fig. 11). Furthermore, all the TDP-43-enriched brain extracts used here are also devoid of other amyloid proteins such as αSyn or tau protein (Extended Data Fig. 9b,c).

As shown in Fig. 7, the PK-treated brain extracts from all three ALS/FTLD-TDP cases efficiently seeded the aggregation of the CP monomers (Fig. 7b–f), although they exhibited differences in seeding efficiency (Fig. 7e). While case 1-derived aggregates, after PK treatment, showed significantly higher seeding activity compared to untreated aggregates, differences in the lag time for the PK-treated and PK-untreated aggregates derived from cases 2 and 3 were less pronounced. This could be due to differences in the levels of truncated and/or modified TDP-43 fragments in these cases compared to case 1.

EM analysis revealed striking differences in the morphologies of the fibrils formed in the three seeded samples with respect to variations in fibrillar width and shapes (Fig. 7h). CP samples seeded with PK-treated extracts from case 1 showed a mixture of fibril morphologies, including single straight and long fibrils (Fig. 7h, red arrows, case 1 +PK cycle 1) and laterally associated fibril bundles (Fig. 7h, white boxes; case 1 +PK cycle 1). However, in samples seeded with brain PK-treated extracts from case 2, single fibrils (Fig. 7h, red boxes; case 2 +PK cycle 1) were observed to be the predominant species. Interestingly, the sample seeded with PK-treated brain extracts from case 3 showed distinct polymorphic structures consisting of short and sturdy fibrillar structures (Fig. 7h, black arrows; case 3 +PK cycle 1) and another population of long and straight fibril (Fig. 7h, blue arrows, case 3 +PK). Comparative width analysis of the single fibrils formed in the seeded samples revealed significant differences, with single fibrils induced by aggregates from cases 1 and 2 having average widths of ~9 and ~7 nm, respectively, whereas single fibrils induced by PK-treated aggregates from case 3 exhibited an average diameter of ~17.5 nm (Fig. 7i). Interestingly, no major differences in the fibril morphologies were observed for all the samples seeded with PK-untreated brain extracts (Fig. 7h, black arrows; case 3 −PK). Interestingly, the final product of the first cycle seeding reactions could seed CP monomers and propagate their structural features in the second cycle of amplification assay (Fig. 7g–i).

Altogether, these results demonstrate that the PK-unmasking protocol combined with the CP-based aggregation assay enables the revelation of the amyloid core, enhances TDP-43 seeding activity and provides indirect evidence of differences in the properties of distinct brain-derived TDP-43 aggregates. Further studies are required to determine the structure of the unmasked core from full-length filaments and to what extent it is similar or different to the CP structure or the core of brain-derived TDP-43 filaments.

## Discussion

We developed and validated a reproducible protocol to generate pure preparations of recombinant FL TDP-43 filaments under physiological conditions (Fig. 1 and Supplementary Figs. 1 and 2). Using Cryo-EM and limited proteolysis, we established the formation of cross-β-sheet-rich fibrils that are buried in the core of FL TDP-43 filaments (Figs. 2 and 3). The amyloid core region of TDP-43 is flanked by highly structured NTD and RRM domains, which results in the complete burial of the amyloid fibril core, rendering it inaccessible to the amyloid dyes. This explains previous reports suggesting that TDP-43 filaments do not bind to the amyloid-specific dyes. Treatment of FL TDP-43 filaments with PK results in the removal of significant parts of the NTD and RRMs domains, exposure of the amyloid core, and a marked increase in ThT binding (Fig. 3). Mass spectrometry analysis of PK-treated samples revealed that the sequence spanning residues 279–360 makes up the core of the fibrils formed by FL TDP-43 (Fig. 4).

Previous studies to elucidate the sequence and structural determinants of TDP-43 aggregation have increasingly relied on fragments of TDP-43, derived mainly from the CTD domain, because of their high propensity to form fibrils with amyloid-like structural and dye-binding properties (Supplementary Table 5). However, the pathological relevance of these fragments is unknown. In addition to their failure to reproduce the structure of brain-derived TDP-43 amyloid filaments, CTD-based peptide model systems do not allow for investigating the role of the flanking domains, which contain most disease-associated mutations and PTMs, in the mechanisms of TDP-43 aggregation. Although several studies have reported conditions for inducing TDP-43 aggregation and amyloid fibril formation in vitro, for the most part, the fibrils generated in these studies are heterogeneous and contain a mixture of morphologies, including amorphous and differently structured aggregates (Supplementary Table 2). Therefore, the methods we developed for the generation of filaments composed of the FL TDP-43 pave the way for systematic studies to elucidate the sequence, molecular and structural determinants of its misfolding and aggregation.

**Extended Data Fig. 10:**
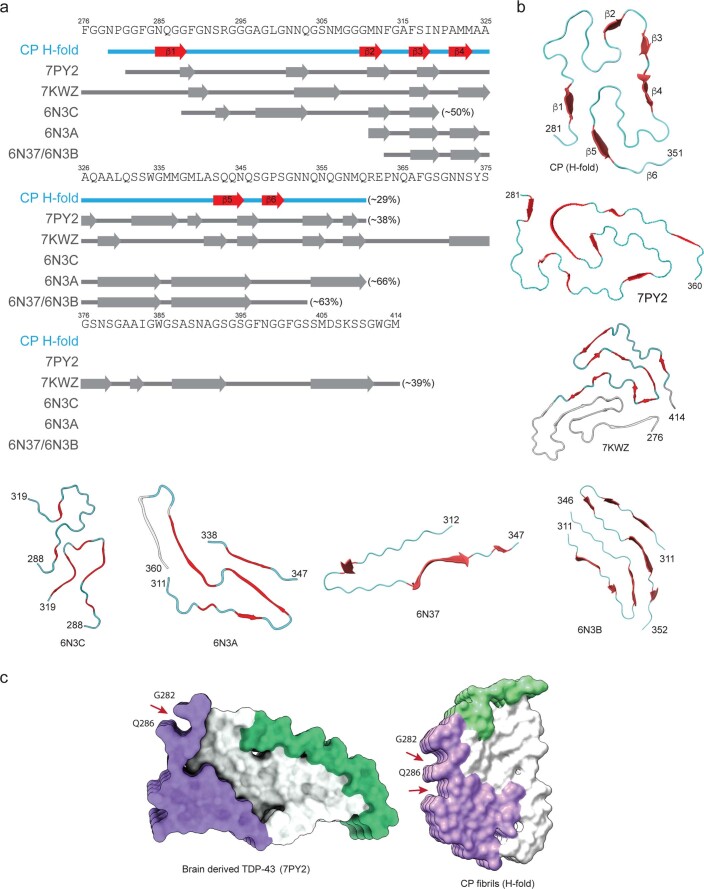
Core peptide and CTD fragments don’t recapitulate brain-derived filament structure. (**a**) Amino acid sequence alignment of the secondary structure (β-strands) of the CP ‘H-fold,’ brain-derived TDP-43 filaments, and recombinant TDP-43 structures with the TDP-43 LC domain and its peptides. PDB IDs are given for the TDP-43 structures. The percentage represents the ratio of β-strand composition to the total peptide length. (**b**) Comparison of structural motifs of ‘H-fold’ TDP43 CP with brain-derived 282–360 TDP-43 filaments from ALS with FTLD (PDB-7PY2)^23^, entire 276–414 TDP43 LCD (PDB 7KwZ)^69^, and short TDP43 LCD fragments 288–319 (PDB-6N3C), 311–360 (PDB-6N3A), 312–347 (PDB-6N37), 312–352 (PDB-6N3B)^68^. (**c**) Comparison of surface representations of cryo-EM structures of TDP-43 core fibrils from brain-derived^23^ and our work. Red arrows point to the grooves on the surfaces of both fibrils. Interestingly, we noted some structural similarities on the surface of the CP fibrils with the brain-derived TDP-43 core structures described by Arseni et al.^23^. For example, a single prominent surface groove formed by the main chain of G282-Q286 and the side chain of Q286 in the brain-derived TDP-43 filaments is also present in our ‘H-fold’ CP structure.

While this paper was in the final stages of preparation, Arseni et al. reported the first cryo-EM structure of the protease-resistant core of TDP-43 filaments isolated from frontal and motor cortices of patients with ALS with FTLD^23^. Consistent with our findings from recombinant FL TDP-43, TDP-43 forms filaments with an amyloid-like structure. We were pleasantly surprised that the structured core of the filament they reported (282–360) is almost identical to the amyloid core sequence we identified by limited proteolysis and mass spectrometry (279–360). Despite their similar sequence, the recombinant peptide 279–360 exhibited a very distinct fold with major differences in secondary structures (β-strands, turns and loops), in comparison with the core amyloid structure of brain-derived TDP-43 filaments or TDP-43 fibrils produced in vitro from the entire CTD domain or shorter CTD-derived peptide fragments (Extended Data Fig. 10). One possible explanation for the differences in the amyloid folds between the brain-derived TDP-43 filaments and the CP fibrils formed by the FL TDP-43 is the presence of the flanking sequences or PTMs that could play an important role in regulating the formation of the amyloid core of brain-derived fibrils. Future studies aimed at solving the PK-resistant amyloid core structure derived from the recombinant FL TDP-43 filaments, using the method we describe here, or from different pathologically relevant C-terminal TDP-43 fragments bearing the residues 279–360, could enable the testing of this hypothesis.

The consistent findings that fibrils from the CTD or CTD-derived fragments possess distinct amyloid core structures compared to that observed from brain-derived TDP-43 fibrils underscore the critical need for systems that enable investigating TDP-43 aggregation in the context of the FL protein. The new method for the generation of highly pure filament preparations of FL TDP-43 described here makes this possible. This paves the way for determining the structure of the FL TDP-43 filaments, which would offer unprecedented insight into how the flanking N-terminal and C-terminal domains contribute to TDP-43 aggregation. For example, the generation of FL TDP-43 filaments from different disease-associated variants or post-translationally modified forms of the protein, followed by treatment with protease and structure determination of the amyloid core, could provide novel insight into how PTMs and disease-linked sequence changes influence TDP-43 aggregation, the final structure of the fibrils and pathology formation and spreading.

Although increasing evidence supports that seeding-directed misfolding and nucleation of TDP-43 aggregation and cell-to-cell propagation as key processes driving TDP-43 pathology spreading in the brain^33,39^, very little is known about the molecular events leading to the formation of the seeding-competent species. TDP-43 filaments found in neuronal inclusions associated with several NDs have been consistently shown to be composed of filaments with diameters of 10–20 nm^22^, which are thinner than what we observed for the intact TDP-43 filaments derived from the full-length protein (Supplementary Table 1)^21,22,40,41^. Therefore, it is reasonable to speculate that most of the filaments detected in brain tissues or isolated from the brain represent filaments where significant segments of the globular domain decorating the core filaments have been cleaved. The fact that different types of brain-derived TDP-43 aggregates (types A to E) could be consistently and efficiently detected and/or immunolabeled with antibodies against extreme C-terminal phosphorylation sites (for example, pSer409/410) suggests that the CTD of many of these filaments remains intact^21,23,40^.

**Fig. 8:**
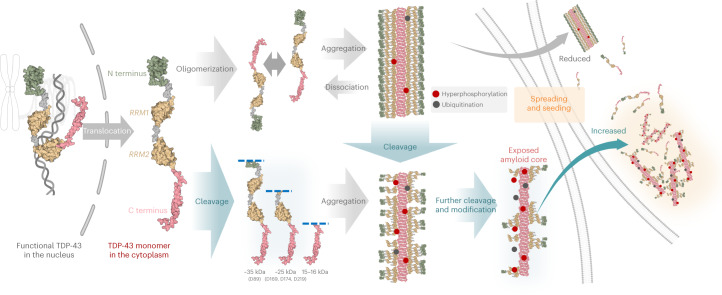
A proposed mechanistic model for the formation of seeding competent TDP-43 fibrils. Schematic of the mechanisms of TDP-43 filament formation, and potential pathways that could lead to the generation of seeding competent cleaved fibrils with exposed amyloid cores.

These observations, combined with previous findings and our in vitro and cell-seeding results (Figs. 6 and 7), lead us to propose that post-aggregation proteolytic processing of TDP-43 and exposure of the amyloid core represent essential steps in the pathogenesis of TDP-43, as this would facilitate and enhance the seeding activity of TDP-43 filaments and possibly other aggregated forms of the protein (Fig. 8). Whether these cleavage events occur before or after TDP-43 filament formation requires further investigation. However, previous time-course studies suggested that FL TDP-43 aggregation precedes the accumulation of TDP-43 CTFs, implying that proteolytic processing of TDP-43 occurs after aggregation and is not required for the initiation of TDP-43 oligomerization and filament formation^40^. Nonaka et al. also showed that PK treatment does not alter the cellular seeding activity of aggregates isolated from diseased brains (FTLD-TDP) or ALS, suggesting that brain-derived aggregates are highly enriched with CTFs with a possibly exposed PK-resistant amyloid core^40^. These findings have significant implications for the mechanisms of TDP-43 pathology formation and point to targeting the proteolysis of TDP-43 monomers and filaments as viable approaches for developing novel therapies to treat TDP-43 proteinopathies based on preventing TDP-43 aggregation, seeding activity, pathology formation and spreading. Therefore, identification of the enzymes that mediate the proteolytic cleavage of TDP-43 filaments and aggregates represents an important first step that could pave the way for novel therapies to slow the spreading of pathology and progression of ALS and other TDP-43 proteinopathies. Interestingly, previous studies from our laboratory showed that post-fibrillization cleavage of the highly negatively charged C terminus of αSyn could be essential for efficiently packing αSyn fibrils within Lewy bodies (LBs)^42^. This is consistent with the C-terminally truncated αSyn fibrils localizing primarily in the core of the LBs^43^. Furthermore, we recently showed that *O*-linked-*N*-acetylglucosaminylation modified αSyn fibrils or post-fibrillization nitrated αSyn fibrils exhibit diminished seeding activity in neurons and in vivo^44,45^. These findings indicate that the PTMs found in pathological aggregates associated with NDs not only are markers of pathology but also serve as key regulators of their processing and pathogenic properties.

Our data also suggest that the sequestration of TDP-43 within the filaments could result in a loss of protein function (Fig. 8). However, this also raises the possibility that the seeding incompetent full-length filaments may represent a functional form of the protein or a reservoir of FL TDP-43 monomers that could be released under specific cellular conditions. To test this hypothesis and the role of PTMs in regulating TDP-43 function in health and disease, it is essential to develop cellular and animal models that faithfully reproduce the formation of FL TDP-43 filaments.

Altogether, our findings contribute to advancing our understanding of the role of TDP-43 aggregation in the pathogenesis of TDP proteinopathies and provide new insights to guide the development of new therapies and diagnostics. For example, our work suggests that upregulating PTMs within the amyloid core sequence and inhibiting the proteolysis of TDP-43 aggregates represent viable strategies to inhibit TDP-43 aggregation and seeding and pathology spreading, respectively. The methodologies and assays we describe should also open new avenues for future studies to: (1) elucidate the sequence, molecular and structural determinants of TDP-43 aggregation, pathology formation, and spreading; (2) develop more robust tools (for example, antibodies) and methods to detect and capture the diversity of TDP-43 pathology in the brain; and (3) develop diagnostic methods based on the detection of differences in the structural properties of native TDP-43 aggregates isolated from human brains. Finally, our work calls for investigating the role of post-fibrillization proteolytic cleavage of TDP-43 in mediating the seeding of other protein pathologies, for example, tau, amyloid-β and αSyn, which increasing studies show to co-occur with TDP-43 pathology^46–48^.

## Methods

### Materials

TDP-43 plasmid constructs used in this study were purchased from ATUM by the insertion of DNA coding for fusion protein (His_6_-Sumo-TDP-43) into a pD451-SR vector. ER2566 *Escherichia coli* (E6901S) competent cells were purchased from New England BioLabs. Isopropyl-ß-d-thiogalactopyranoside was ordered from Applichem (A1008,0025). ThT was purchased from Sigma. ThS was purchased from Fluka. Phenyl methane sulfonyl fluoride (PMSF) was purchased from Axonlab (A0999.0005). Trifluoroacetic acid (TFA) was purchased from Sigma. The primary antibodies such as rabbit polyclonal full-length TDP-43 antibody (18280-1-AP), rabbit N-terminal-specific TDP-43 antibody (10782-2-AP) and rabbit C-terminal-specific TDP-43 antibody (12892-1-AP) were purchased from Proteintech and others included C-terminal anti-TDP-43 antibody (C89; 1:3,000 dilution; in-house CNDR) and p409–410 (1:200 dilution; Ascenion, TAR5P-1D3)^49^. The secondary goat anti-rabbit labeled with Alexa Fluor 680 was purchased from Invitrogen. Goat anti-Rabbit IgG (H&L) was purchased from Aurion (810.011). The PageRuler prestained protein ladder (26617), SeeBlue Plus2 prestained protein standard (LC5925) and the SnakeSkin dialysis tubing (68700) with a molecular-weight cutoff of 7 kDa were purchased from Thermo Scientific.

### Expression and purification of His_6_-Sumo-TDP-43 and His-Sumo-TDP-43 CP

The respective TDP-43 plasmids were transformed into *E. coli* ER2566 cells using a chemical competent method. An isolated single colony from the LB-agar plate was inoculated to 200 ml LB medium containing kanamycin (25 mg ml^−1^) and incubated at 30 °C for 20 h with shaking at 180 r.p.m. Bacterial overexpression was initiated by mixing the pre-culture medium with 3 l LB medium to obtain the OD_600_ of 0.05 and continued to grow at 30 °C until the OD_600_ reached between 0.4 and 0.5. Cells were then induced with 0.4 mM IPTG, growth was continued overnight at 15 °C and pelleted by centrifugation at 4,160*g* for 10 min at 4 °C.

Purification of His_6_-Sumo-TDP-43 was started immediately by resuspending cells in 80 ml lysis buffer (buffer A: 30 mM Tris, 500 mM NaCl, 1 mM dithiothreitol, 20 mM imidazole, 10% glycerol (vol/vol), pH 8.0) with the addition of 0.3 mM PMSF and two protease inhibitor tablets (Roche). Cell lysis was carried out on the ice by sonication (5 min and 3 min, pulse on 30 s, pulse off 30 s, 70% amplitude) using a Vibra cell VCX130 from Sonics. Centrifugation (40 min, 17,217*g*, 4 °C) was followed to separate the supernatant from the crude cell lysate. Next, 12 μl of Benzonase Nuclease HC (Novagen) was added to the supernatant and incubated at room temperature for 30 min with mild stirring. Following that, the supernatant was filtered through 0.45-μm syringe filter membranes and loaded onto a 20-ml Ni-NTA affinity column (GE Healthcare) pre-equilibrated with five column volumes of buffer A. Weakly bound nonspecific proteins were washed off from the column by passing of ten column volumes of buffer A at a flow rate of 2 ml min^−1^. Imidazole gradient with the buffer B (30 mM Tris, 500 mM NaCl, 1 mM dithiothreitol, 500 mM imidazole, 10% glycerol (vol/vol), pH 8.0) from 5% to 100% was carried out to elute His_6_-Sumo-TDP-43. Elution fractions were analyzed to identify the fractions containing high-purity His_6_-Sumo-TDP-43 using SDS–PAGE and analytical C8 reversed-phase ultra-high performance liquid chromatography (UPLC).

### Purification of native full-length TDP-43

Fractions identified for high-purity His_6_-Sumo-TDP-43 were pooled, added with ubiquitin-like-specific protease 1 (His_6_-Ulp-1, prepared in-house; ~1:10 mass ratio), and incubated overnight at 4 °C to cleave the native full-length TDP-43 (FL TDP-43) from the His_6_-Sumo fusion. Cleavage was monitored using UPLC and SDS–PAGE analysis. Subsequently, a cleaved sample was filtered through 0.45 μm syringe filter membranes and the buffer was exchanged with reverse-IMAC buffer A (30 mM Tris, 500 mM NaCl, 1 mM dithiothreitol, 10 mM imidazole, 5 % glycerol (v/v), pH 7.0) using HiPrep^TM^ 26/10 desalting column.

Using reverse-IMAC, native FL TDP-43 was purified from the rest of the His_6_-tag proteins by passing the Ulp-1 treated sample into a 5 mL Ni-NTA affinity column (GE Healthcare) pre-equilibrated with 5 column volumes of reverse-IMAC buffer A. One step imidazole gradient was performed with buffer B (30 mM Tris, 500 mM NaCl, 1 mM dithiothreitol, 500 mM imidazole, 5 % glycerol (v/v), pH 7.0). Native TDP-43, lacking the His_6_ tag, elutes in the wash fractions. His_6_ tag proteins bound to the column eluted at 100% buffer B concentration. Collected fractions were analyzed using UPLC and SDS–PAGE. Protein concentration was determined by BCA assay (Pierce, catalog no. 23227). The molecular weight of His_6_-Sumo-TDP-43 and native FL TDP-43 was measured using LC–MS-LTQ ion trap mass spectrometer (Thermo Scientific) having the C3 poroshell 300SB 1.0 × 75-mm 5-μm column from Agilent (5–95% acetonitrile in 5 min, flow rate of 0.3 ml/min, injection volume of 10 μl). All obtained LC–MS spectra were deconvoluted with MagTran software version 1.03b from Amgen. Theoretical molecular weights of His_6_-SUMO-TDP-43 and full-length TDP-43 are 57775.2 Da and 44608.4 Da, respectively.

### Purification of TDP-43 (279–360) core peptide

Fractions identified for His_6_-Sumo-TDP-43 (279–360) CP were pooled, added with Ulp-1 (His_6_-Ulp-1, prepared in-house; ~1:10 mass ratio) and incubated overnight at 4 °C to cleave the TDP-43 (279–360) CP from the His_6_-Sumo fusion. Cleavage was monitored using UPLC and mass spectrometry analysis. Subsequently, a cleaved sample was filtered through 0.45-μm syringe filter membranes and injected into a reverse-phase HPLC C18 (10 μm, semi-prep; Phenomenex) column, and the peptide was eluted using buffer A, 0.1% TFA in water, and buffer B, 0.1% TFA in acetonitrile. The purity of the elution from HPLC was analyzed by UPLC and electrospray ionization (ESI)–MS. Fractions containing highly pure TDP-43 (279–360) CP were pooled, snap frozen and lyophilized.

### In vitro TDP-43 filament formation

Purified FL TDP-43 fractions at a concentration of 5 µM were dialyzed against the fibril-forming buffer (FFB: 30 mM Tris, 100 mM NaCl, 1 mM dithiothreitol pH 7.4) using a SnakeSkin dialysis tubing with a molecular-weight cutoff of 7,000 Da (Thermo Fisher). Following overnight dialysis, the fibrillization of TDP-43 was performed in FFB for 5 d at room temperature. This resulted in the aggregation of TDP-43 from the oligomeric prefibrillar form to mature TDP-43 filaments over the total incubation period. After 5 d, TDP-43 fibrils were isolated from coexisting oligomeric species by centrifugation of the incubation buffer at 25,000*g* for 30 min at 4 °C (Fig. 2a). The pellet fraction containing only TDP-43 fibrils was resuspended in the buffer having 30 mM Tris, 100 mM NaCl at pH 7.4. The content of TDP-43 in the pellet and supernatant was estimated using SDS–PAGE analysis.

### Far-ultraviolet circular dichroism spectroscopy

CD spectra of TDP-43 samples, loaded in a quartz cuvette with 1-mm path length, were collected using a Jasco J-815 CD spectrophotometer operated at 20 °C within the range of 200–250 nm. Data acquirements used the following parameters: data pitch, 0.2 nm; bandwidth, 1 nm; scanning speed, 50 nm min^−1^ and digital integration time, 2 s. The spectrum of each sample is the average of ten repeats followed by a binomial approximation and plotted as the mean residue molar ellipticity.

### Attenuated total reflectance-Fourier transform infrared spectroscopy

Spectra were recorded on a Tensor 27 Fourier transform infrared spectroscopy spectrometer (Bruker) equipped with a BIO-ATR II cell and a photovoltaic LN-MCT detector cooled with liquid nitrogen. Around 30 µl of the sample was placed onto the zinc selenide (ZnSe) crystal of the BIO-ATR II cell. Spectra represent averages of 128 scans at room temperature, using an aperture of 4 mm and an instrument resolution of 4 cm^−1^ with four times zero filling.

### Denaturing gel electrophoresis

Samples for SDS–PAGE were mixed with 2× Laemmli buffer and loaded onto 15% polyacrylamide gels. The gel was run at 180 V for 1 h in a running buffer (Tris-glycine-SDS running buffer), followed by staining with a solution containing 20% (vol/vol) isopropanol, 10% acetic acid (vol/vol) and 0.05% (wt/vol) Coomassie brilliant blue R and destaining with boiling distilled water.

### Fluorescence spectroscopy

Samples for ThT fluorescence assay were prepared with pelleted TDP-43 filaments at the concentration of 250 µg ml^−1^ and αSyn fibrils at 150 µg ml^−1^ were mixed with 20 µM ThT in 30 mM Tris and 100 mM NaCl at pH 7.4. All fluorescence spectra obtained were an accumulation of three curves, recorded using FluoroMax3 spectrometer (Horiba) at room temperature using an excitation wavelength of 450 nm with a slit width adjusted to 1 nm and emission wavelength from 460 to 650 nm with a slit width at 5 nm. For ThS fluorescence, samples were mixed with 20 mM ThS and measured using the same settings as described above.

### Congo Red absorbance spectroscopy

FL TDP-43 filaments at 5 µM or αSyn fibrils at a concentration of 5 µM in 30 mM Tris and 100 mM NaCl at pH 7.4 was added with 20 µM CR and incubated for 5 min at room temperature. Following this, CR absorbance spectra were collected at 400–600 nm using a Cary 100 Bio UV–vis spectrophotometer (Varian, CH) at room temperature. The stock solution of CR was prepared in 30 mM Tris and 100 mM NaCl at pH 7.4 and 1 mM concentration and filtered using a 0.45-µm filter.

### Western blot analysis

Following electrophoresis, the proteins in the gel were transferred onto nitrocellulose membranes (0.45 μm; Omnilab) using a semidry transfer system (Bio-Rad) under a constant current of 0.6 A at 20 V for 1 h. The membranes were then blocked for 1 h at room temperature in a 1:3 ratio of Odyssey blocking buffer (Li-COR Biosciences):PBS. The membranes were then probed with the respective TDP-43 (full-length) antibody (18280-1-AP) overnight at 4 °C. After three washes with PBS buffer containing 0.1% (vol/vol) Tween 20 (Fluka), the membranes were incubated with secondary goat anti-rabbit antibody conjugated to Alexa 680 (dilution 1:5,000; Invitrogen). The membranes were then washed three times with PBS buffer containing 0.01% (vol/vol) Tween 20, once with PBS buffer, and scanned on a Li-COR scanner at 700 nm.

### Transmission electron microscopy

Before the application of the sample, Formvar and carbon-coated 200 mesh containing copper EM grids (Electron Microscopy Sciences) underwent a glow discharge for 30 s at 20 mA using a PELCO easiGlow Glow Discharge Cleaning System (TED PELLA). Subsequently, 5 μl of the sample was placed onto the EM grids and waited for a minute. Then, samples in the grids were carefully blotted out using filter paper and air-dried for 30 s. Following that, the grids were washed three times with ultrapure water and 0.7% (wt/vol) uranyl formate solution, respectively. Grids were examined using a Tecnai Spirit BioTWIN electron microscope. The microscope was equipped with a LaB6 gun, operated at an acceleration voltage of 80 kV, and images were captured using a 4k × 4k charge-coupled device camera (FEI Eagle). The crossover distance and the width of the helical filaments were measured manually on the EM images using ImageJ software. For each filament, the average crossover distance and width were calculated and plotted in the graphs using OriginPro software. A total of 21 helical FL TDP-43 filaments were evaluated from the acquired EM images.

### Immunogold labeling

For immunogold labeling of filaments, 5 μl of TDP-43 filaments were pipetted onto glow-discharged Formvar/carbon-coated grids, blotted off of the excess samples after 2 min, and washed two times with autoclaved distilled water. The grids were then blocked with 0.1% bovine serum albumin (BSA, Aurion) in PBS (Dulbecco) for 5 min. Following that, grids were incubated in a water-saturated chamber for 2 h with primary antibodies of anti-TDP-43 specific for N terminus and C terminus (Proteintech) diluted at 1:100 in 1% BSA in PBS. Later, the samples were washed four times with PBS drops. The grids were then incubated with 10-nm gold-conjugated anti-rabbit antibody (Aurion) diluted at 1:25 in PBS, for an hour in a water-saturated chamber. After rinsing three times (each rinsing is for 5 s) and washing for four times (each washing is for 2 min incubation) with PBS drops, the samples in the grids were cross-linked using 1% glutaraldehyde in PBS for 5 min. Finally, the grids were washed with ten drops of autoclaved distilled water with incubation of 1 min at each drop. Grids were then negatively stained with three drops of 0.7% (wt/vol) uranyl formate solution. Immunogold-labeled grids were examined at transmission EM as described above.

### Proteolysis of fibrillar TDP-43

A reaction mixture consisting of 750 μl of TDP-43 filaments at the concentration of 250 μg ml^−1^ was added with 5 μl of PK (0.4 mg ml^−1^, Promega) solution or 5 μl of trypsin (1 mg ml^−1^, Promega) solution prepared in Tris calcium buffer (50 mM Tris, 100 mM NaCl, 1 mM CaCl_2_, pH 7.4) incubated at 37 °C. At 0, 5, 15, 30 and 60 min, 150 μl from the reaction mixture (PK and trypsin) was taken out, and we added 2.5 μl of 300 mM PMSF prepared in dimethylsulfoxide (DMSO; Sigma) to inhibit the proteolysis. Samples at these different time points were further used for transmission EM and dot blot analysis.

### Dot blot analysis

Around 20 μl of protease-treated and untreated TDP-43 fibrils at different time points (see above) was loaded in replicates (*n* = 3) on the nitrocellulose membranes (0.45 μm; Omnilab) packed using a 96-well dot blot apparatus (GE Healthcare) connected under vacuum conditions. After the adsorption of samples, the membrane was washed twice with PBS-T (PBS with 0.1% Tween 20, pH 7.4) while still packed in the dot blot apparatus. Following that, the membrane was blocked in a 1:3 ratio of Odyssey blocking buffer (Li-COR Biosciences):PBS-T. After an hour of incubation at room temperature, the membrane was treated with respective TDP-43 N terminus and C terminus (Proteintech) overnight at 4 °C. After three washes with PBS-T, the membrane was incubated with secondary goat anti-mouse or anti-rabbit antibodies conjugated to Alexa Fluor 680 (dilution of 1:5,000; Invitrogen). The membrane was then washed three times with PBS-T, once with PBS buffer and scanned on a Li-COR scanner at 700 nm. The intensities of antibody staining were quantified using the densitometry analysis tool from ImageJ (Fiji) software. The intensity of undigested TDP-43 fibrils at time 0 was used as standard and set to 100%.

### LC–MS/MS analysis

In total, 300 μl of TDP-43 filaments at 250 μg ml^−1^ was digested with 2 μl of PK (0.4 mg ml^−1^, Promega) solution prepared in Tris calcium buffer (50 mM Tris, 100 mM NaCl, 1 mM CaCl_2_, pH 7.4) incubated at 37 °C for 30 min. Following inhibition with 5 mM PMSF, samples were ultracentrifuged (Beckman Coulter) for 45 min at 200,000*g*. After the centrifugation, the supernatant was removed and the remaining pellet was washed twice with 30 mM Tris, and 100 mM NaCl pH 7.4 to remove the soluble species after cleavage. To the pellet, 100 μl of a 1:1 TFA/HFIP solution was added and incubated at room temperature for 30 min under mild shaking conditions for the disaggregation of the core. The TFA/HFIP solution was removed using a gentle nitrogen stream, and the sample tubes were flash-frozen in liquid nitrogen and lyophilized. The lyophilized material was dissolved in 30–40 μl of 2% acetonitrile/0.1% formic acid for subsequent LC–MS/MS analysis.

For the MS/MS analysis of non-fibrillar TDP-43 following the PK treatment, non-fibrillar TDP-43 (purified fresh from reverse-IMAC purification) was buffer exchanged to Tris calcium buffer (50 mM Tris, 100 mM NaCl, 1 mM CaCl_2_, pH 7.4), adjusted to a concentration of 250 μg ml^−1^ and added 2 μl of PK (0.4 mg ml^−1^; Promega). Following the incubation for 30 min at 37 °C, 5 mM PMSF was added and subsequently used for LC–MS/MS analysis.

In total, 15 μl of samples were analyzed using ESI in positive ion mode (3.5 kV ionization voltage) on an LTQ Orbitrap XL (Thermo Fisher), equipped with an Accela Pump HPLC and a CTC ThermoPAL autosampler (Thermo). Peptides were separated on a 4.6 mm inner diameter × 75 mm length column, packed with 3.5 μm, 100 Å Symmetry C18 (Waters). LC–MS/MS was performed using a 25-min gradient at a flow rate of 150 μl min^−1^ with a mixture of 0.1% formic acid in 2% acetonitrile (mobile phase A) and 0.1% formic acid in 98% acetonitrile (mobile phase B). The mass spectrometer operated in a data-dependent MS/MS mode with a full scan range of 400–1,600 *m/z* and a resolving power of 60,000 *m/z*. The five most intense ions detected from the MS1 survey scan were selected for fragmentation by collisional induced dissociation (CID) at 35% normalized collision energy over 30 ms reaction and with dynamic exclusion enabled for 40 s, to minimize repeated selection of the same parent ion. An isolation width of 2 *m/z* was used for parent ion selection.

After data acquisition, raw data were converted into peak list (mzML) using ProteoWizard tools^50^. Peptides were identified with the X! Tandem search engine as an internal tool of OpenMS platform^51^, using TDP-43 as a sequence database. An accuracy of 10 ppm and 0.5 Da was used for parent and fragment ions assignment, respectively. The search was carried out with no specificity and with the maximum missed cleavages admitted. Oxidation on methionine residues was selected as a variable modification. The most representative peptide spectrum matches of the identification list were selected for ion chromatogram extraction and mass deconvolution.

### Total internal reflection fluorescence microscopy

Around 10 μl of samples containing FL TDP-43 filaments or PK-unmasked FL TDP-43 filaments in 30 mM Tris buffer pH 7.4 with 100 mM NaCl were mixed with 10 μM ThT and were pipetted into a laboratory-made flow cell on a glass slide, sealed using nail polish and incubated overnight upside down at room temperature in a dark chamber. Samples were visualized on a Nikon Eclipse Ti microscope by mounting on a Plan Apo ×100/1.45 oil TIRFM objective (Nikon) in TIRF mode. ThT in the samples was excited with 405 nm laser and emission was collected using an excitation 455/10-nm and emission 485/30-nm bandpass filter cube (TIRF). Fluorescence images were captured using an ANDOR camera.

### Quantification of thioflavin-T fluorescence

Following the protein concentration estimation using BCA assay, 200 μl of samples containing FL TDP-43 filaments or PK-unmasked TDP-43 filaments (at two different amounts of 20 μg and 40 μg for each) in 30 mM Tris buffer at pH 7.4 with 100 mM NaCl was taken in different microcentrifuge tubes. To each of which, 10 μM ThT was added. For every sample, a triplicate of 60 μl was pipetted into a 384-well plate (Falcon, 353962). ThT fluorescence was measured using a 440-nm excitation filter, and a 480-nm emission filter set at 37 °C. The final ThT fluorescence values shown are the average of three independent recorded triplicates and error bars show the s.d. of the triplicate measurements.

### Atomic force microscopy

All samples used for AFM measurements were incubated at room temperature on freshly cleaved and positively functionalized mica discs. Functionalization was done by a 10-μl droplet of 0.5% (vol/vol) APTES (3-aminopropyl) triethoxysilane deposition on a freshly cleaved mica surface. After 1 min of incubation, APTES was flushed three times with 1 ml of milli-Q water and the surface was dried by a gentle stream of nitrogen gas. Ten microliters of each sample diluted in PBS buffer was incubated at room temperature for 10 min on freshly functionalized mica. After 10 min, the sample was rinsed with 1 ml of milli-Q water and the mica surface was dried by a gentle nitrogen stream.

AFM measurements and image processing were performed as described^52^. All images were obtained using the NX10 Atomic Force Microscope (Park Systems) in non-contact amplitude modulation mode in ambient conditions. Commercially available silicon ultra-sharp tips (SSS–NCHR, Park Systems) with a nominal tip radius of 2 nm and nominal resonance frequency of 330 kHz were used. Close to the surface, tip–sample interaction forces cause a reduction in the amplitude of the oscillation and a phase shift lag or advance. The phase shift signal contains information regarding the specific nature of the tip–sample interaction dissipative forces. Mechanical interaction between the tip and the sample can introduce imaging artifacts or damage the TDP-43 filaments, altering in both cases the recorded features. To control these interactions, phase images were recorded simultaneously with height images. Height images were used for further analysis of a sample and phase images were used as a control of tip–sample interactions. To consistently compare the topographical features, only images obtained with a comparable phase shift (≈ ∆20° along the fibril axis) were used in further analysis. All images were recorded at 1,024 × 1,024 pixels for 4 × 4 mm^2^ of sample area and were subsequently flattened with XEI software (Park System) with the same guidelines to keep consistency between different samples. Images were flattened with 1st regression order first by plane and then line by line until images reached a flat baseline on their profile. During this process, all structures were masked from software calculations to avoid any modification of their height.

For the height analysis of the filaments, home-build software ‘DNA trace’ was used^52^. ‘DNA trace’ allows a connection of the highest points along the fibril axis within a given cross-sectional area on the AFM image. A constant step for tracing equal to 4 nm was kept for all analyzed images, which corresponds to 2 pixels on 4 × 4 mm^2^ images. The average height of a single filament was obtained by calculating the mean height of all traced and connected points along the filament axis. To avoid the artificial height due to an increase in the calculations of the mean, areas of crossing between filaments were excluded. For filaments with a length exceeding the area of an image, only the longest part was traced. In this case, only one representative part of filament per structure was taken to avoid a false count of measured structures. Histograms with the distribution of filament height were created using the OriginPro software (OriginLab).

### Thioflavin-T aggregation assay

Lyophilized TDP-43 (279–360) CPs in microcentrifuge tubes were weighed in a balance (Sartorius) and added a few microliters of dry DMSO (final concentration at 2.5% vol/vol) was added to the film of the CP, followed by the addition of FFB to give a final concentration of CP at 100 μM. A stock solution of ThT at 500 μM was also prepared. For the ThT-based aggregation assay, 400 μl of respective samples were prepared to have the final composition of 10 μM of CP, 10 μM of ThT, and respective percentages (% wt/vol) of different seeds (see below for the preparation of each seed). For every sample, a triplicate of 120 μl each were pipetted into a polybase black 96-well plate with an optical bottom (Corning). Aggregation kinetics were carried out in a FLUOstar Omega plate reader (BMG Labtech) by measuring the ThT fluorescence as a function of time (min) using a 440-nm excitation filter, and a 480-nm emission filter set at 25 °C. ThT fluorescence was monitored online by obtaining the data points once every 300 s, and before every measurement, the plate was shaken at 100 r.p.m. for 5 s. The aggregation curves of every sample shown are the average of three independent recorded triplicates and error bars show the s.d. of the triplicate measurements.

### Preparation of seeds

#### Core peptide fibrils seeds (CP fibrils)

Lyophilized TDP-43 (279–360) CP was added with dry DMSO (2.5% vol/vol) and FFB to have the final concentration of 50 μM in 400 μl. Following 3 d of incubation at room temperature under no shaking conditions, the presence of fibrils was confirmed by EM analysis. Seeds were prepared by sonication of the fully grown core fibrils at 400 ml using a probe sonicator (Sonic Vibra Cell, Blanc Labo) for 1 s on/1 s off cycle for 5 s at 40% amplitude followed by one more time of sonication for 1 s on/1 s off cycle for 5 s at 20% amplitude.

#### Full-length TDP-43 filaments seeds (FL TDP-43)

Following the preparation of filaments, seeds were prepared by sonication using a probe sonicator (Sonic Vibra Cell, Blanc Labo) for 1 s on/1 s off cycle for 5 s at 40% amplitude followed by one more sonication for 1 s on/1 s off cycle for 5 s at 20% amplitude.

#### Proteinase K-treated TDP-43 filaments seeds (proteinase K-unmasked TDP-43 filaments)

In total, 300 μl of TDP-43 filaments at 250 μg ml^−1^ was added with 2 μl of PK (0.4 mg ml^−1^, Promega) solution prepared in Tris calcium buffer (50 mM Tris, 100 mM NaCl, 1 mM CaCl_2_, pH 7.4) incubated at 37 °C for 30 min. Following inhibition with 5 mM PMSF, samples were ultracentrifuged (Beckman Coulter) for 45 min at 100,000*g*. After centrifugation, the supernatant was removed and the obtained pellet was washed twice with 30 mM Tris, and 100 mM NaCl at pH 7.4 to remove the soluble species after cleavage. To the pellet, 150 μl of 30 mM Tris and 100 mM NaCl at pH 7.4 was added and resuspended very well by vertexing. Seeds were prepared by sonication using a probe sonicator (Sonic Vibra Cell, Blanc Labo) for 1 s on/1 s off cycle for 5 s at 40% amplitude followed by one more sonication for 1 s on/1 s off cycle for 5 s at 20% amplitude.

### Brain seeds

Sarkosyl-insoluble TDP-43 protein from the frozen frontal cortex of FTLD-TDP participants and control cases was prepared as described previously^33^. Human postmortem brains were obtained from the University of Pennsylvania, CNDR Brain Bank^53^. All necessary written informed consent forms were obtained from the participants or their next of kin in accordance with University of Pennsylvania Institutional Review Board guidelines and confirmed at the time of death. For each brain-derived extract, 10 µl of each sample (~15–40 µg total protein) was used to prepare the PK-treated and PK-untreated seeds (+PK and –PK, respectively). Then, 20 μl of brain extract was added with 80 μl of FFB and split into two 50-μl aliquots. To one 50-μl aliquot, 0.5 μl of PK (0.4 mg ml^−1^, Promega) was added with solution prepared in Tris calcium buffer (50 mM Tris, 100 mM NaCl, 1 mM CaCl_2_, pH 7.4 (+PK). To the other aliquot, 0.5 μl of FFB was added and incubated at 37 °C for 15 min (–PK). Both samples were incubated at 37 °C for 15 min, followed by the addition of 1 μl of 300 mM PMSF. Finally, samples were water bath sonicated for 30 s and added to 350 μl of TDP-43 CP aggregation assay reaction mixture having the final 10 μM CP concentration and monitored for ThT-based aggregation kinetics as mentioned before.

### Immunogold labeling of brain extracts

Sarkosyl-insoluble brain extracts were diluted (1:5 times for case 1, and 1:2 times for cases 2 and 3, respectively) with PBS (Dulbecco). For the immunogold labeling, 4 μl of diluted brain extracts was pipetted onto a glow-discharged Formvar/carbon-coated grids, blotted off of the excess samples after 2 min and washed two times with autoclaved distilled water. The grids were then blocked with 0.1% BSA (Aurion) in PBS (Dulbecco) for 5 min. Following that, grids were incubated in a water-saturated chamber for 2 h with rabbit polyclonal primary TDP-43 antibody (C2089, epitope: 394–414)^33^ diluted at a 1:40 ratio in 1% BSA in PBS. Later, the samples were washed four times with PBS drops. The grids were then incubated with 10-nm gold-conjugated anti-rabbit antibody (Aurion) diluted 1:20 in PBS, for 1 h in a water-saturated chamber. After rinsing three times (each rinsing is for 5 s) and washing for four times (each washing was for a 2-min incubation) with PBS drops, the samples in the grids were cross-linked using 1% glutaraldehyde in PBS for 5 min. Finally, the grids were washed with ten drops of autoclaved distilled water with incubation of 1 min at each drop. Grids were then negatively stained with three drops of 0.7% (wt/vol) uranyl formate solution. Immunogold-labeled grids were examined at transmission EM as described above.

### Immunoblotting TMEM239

In total, 5 µl of sarkosyl-insoluble fraction was resolved on 12% Bis-Tris gels (NuPAGE Novex, Thermo Scientific) using MOPS-SDS as a running buffer. Proteins were transferred onto 0.45-μm nitrocellulose membranes and blocked with Rockland blocking buffer (MB-070). Membranes were immunoblotted overnight at 4 °C with the rabbit polyclonal antibody TMEM239 at a 1:2,000 dilution in blocking buffer^35^. Blots were scanned using an Odyssey infrared system (ODY-2816 Imager) and the images were analyzed with the Image Studio software (LI-COR Biotechnology).

### Cryo-EM data collection

Fibrils from the TDP-43 CP (279–360) were screened with a negative-staining (NS) transmission EM for fibril concentration and morphology. Aliquots of optimized fibril samples were applied onto gold Ultrafoil 1.2/1.3 grids and plunge frozen in liquid ethane with a Vitrobot Mark IV (Thermo Fisher Scientific: TFS). Frozen cryo-EM grids were imaged on a TFS 300 kV Titan Krios at the NeCEN facility. Images were collected at the NeCEN facility on a K3 electron counting direct detection camera (Gatan) in counting mode (non-CDS mode; 40 fractions) using the EPU 2.6.1 (Thermo Fisher Scientific) at a magnification of ×80,000 (physical pixel size 1.06 Å) and a total dose of 40 electrons per square angstrom (e^−^/Å^2^) for each exposure, and defocus range of −0.9 to 2.0 microns. In total, 3,171 movies were stored in gain-normalized tiff format.

### Image processing

A total of 3,171 movies were imported into RELION (v3.1)^54,55^, drift-corrected and dose-weighted, and estimated for contrast transfer function (CTFn). One hundred micrographs were manually inspected to select a representative set of nonoverlapping straight filaments using the e2helixboxer.py from EMAN2 (ref. ^56^). Dose-weighted averages were denoised with JANNI and subjected to the semi-automated filaments tracing with crYOLO (v1.7.5)^57^. Filament start-end coordinates in STAR format were imported into RELIONv3.1, and helical segments were extracted using a box size of 256 pixels (271 Å) and 10% inter-box distance. A total of ~10^6^ segments were extracted and subjected to multiple rounds of the reference-free 2D classification to select only segments with clear 4.86-Å β-strand separation along the fibril axis, measured from the 2D class-average layer line profile. The helical twist was determined by measuring a half-pitch distance from 2D class-average images of 1,024 pixels extracted and scaled to 256 pixels segments. Initial 3D reference structure was generated with relion_helix_inimodel2d from set of representative 2D class averages and estimated helical parameters. The best segments were subjected to subsequent 3D classifications with the generated 3D initial reference. Several rounds of 3D classification with *T* = 25 and ‘ignore CTFns unit first peak’ option resulted in 8,104 and 5,795 segments contributed to the first (one protofilament) and second (two protofilaments) conformations of TDP-43 CP fibrils.

The best segments of both conformations were subjected to additional rounds of 3D classification with a single class and local optimization of helical twist and rise, resulting in well-separated β-strands and resolved large side chains. The resulting 3D classes were subjected to 3D refinement iterated with particle polishing and CTFn refinement^58,59^. Finally, the resulting 3D reconstructions were post-processed with RELION, and the global resolution was measured, 4.1 Å for the first conformation and 3.7 Å for the second conformation. Post-processed maps were symmetrized with refined helical parameters for both conformations. A second conformation with 3.7-Å resolution and resolved large side chains were used for model building. Both were assumed to have a left-handed twist, according to the handedness of previously reconstructed TDP-43 filaments.

### Model building

The CP atomic model was subsequently built de novo into post-processed and symmetrized maps with COOT^60^. Large side chains of 334 TRP, 316 PHE, 313 PHE, and a few others were used as a guide. Both N−C and C–N orientations were built, and the model with the best fit of side chains was used for subsequent refinement. An initial model was used to build five helical symmetry-related copies with MolRep from CCPEM^61^, which were subjected to real-space refinement with NCS restraints in PHENIX^62^. After several rounds of refinement, side chains were manually inspected and adjusted for the energy-favored geometry in COOT. The final model was validated using MolProbity^63^ and Protein Data Bank (PDB) validation service from wwPDB^64^. Accurate details of the data acquisition and final statistics of the refined model are given in the Supplementary Table 6.

### Cellular TDP-43 aggregation assay

iGFP-NLSm cells (clone 6.B7) were plated on poly-d-lysine-coated coverslips (30,000 cells per well) and transduced after 24 h with 0.25 µg of recombinant TDP-43 monomers, CP monomers, FL TDP-43 filaments, CP fibrils or PK-unmasked TDP-43 filaments. Briefly, samples were diluted with dPBS and mixed with single-use tubes of BioPORTER as a protein delivery reagent (BP509696, Genlantis) as previously described^33,34^. Protein–Bioporter complexes were added to the cells and incubated for 4 h. Cells were placed back on fresh medium in presence of doxycycline (1.0 μg ml^−1^) and cultured for 3 additional days (3 d.p.t.).

### Immunocytochemistry

iGFP-NLSm were fixed with 4% paraformaldehyde (PFA) in PBS and permeabilized with 0.1% Triton X-100 for 15 min. When indicated, to remove cytoplasmic soluble proteins and quantify the formation of TDP-43 pathology, cells were fixed in 4% PFA containing 1% Triton X-100 for 15 min at room temperature as previously described^33,34^. Briefly, after blocking, cells were incubated with the monoclonal antibody phospho-specific p409–410 antibody overnight at 4 °C. After three washes with dPBS, cells were incubated with appropriate secondary antibodies in a blocking buffer for 1 h at room temperature in the dark. Coverslips were mounted with Fluoromount G containing DAPI (Thermo Scientific).

### Quantification of TDP-43 pathology

To quantify the extent of phospho-positive aggregates, whole coverslips were scanned and images were digitalized using a Lamina Multilabel Slide Scanner (Perkin Elmer). The images were processed using the Indica Labs HALO software, and the percentage area occupied by p409–410 immunopositive staining was quantified automatically using HALO^33,65^. The number of DAPI-positive nuclei (no. DAPI/µm) was quantified using HALO.

### Live cell-imaging

Live cell-imaging uptake analysis was performed in QBI-293 cells plated in poly-d-lysine-coated (100 μg ml^−1^; P0899, Millipore Sigma) Quad Ibidi dishes with glass coverslips (35 mm, Ibidi, 80416). Next, 2.5 µg of Atto 488-labeled FL TDP-43 filaments, PK-unmasked TDP-43 filaments and CP fibrils were diluted with dPBS, sonicated and mixed with single-use tubes of BioPORTERTM as a protein delivery reagent (BP509696, Genlantis). Protein–Bioporter complexes were added to the cells and after 4 h of incubation the transduction mix was removed and OptiMEM containing 100 μM of trypan blue was added to the cells to quench the extracellular labeled material. The culture dish was secured inside a moist chamber maintained at 37 °C in a humidified atmosphere containing 5% CO_2_ and cells were imaged at ×40 in a high-resolution Leica DMI6000B microscope using the Leica LASX software.

### Preparation of fluorescently labeled FL TDP-43 filaments, PK-unmasked TDP-43 filaments and CP fibrils

Different forms of TDP-43 aggregates including the FL filaments, PK-treated TDP-43 filaments and CP fibrils were taken at a concentration of 10–20 μM in a final volume of 300 μl of PBS and the pH was adjusted to 8.5. One equivalent of Alexa Fluor 488 maleimide was added and incubated at 4 °C overnight. The labeled fibrils/filaments were then ultracentrifuged at 100,000*g* for 1 h at 4 °C. The supernatants were collected and stored for later SDS–PAGE analysis. The sedimented pellets were resuspended in PBS. This wash step was repeated (three times) until the dye in excess was removed. The samples collected during the repeated centrifugations steps were loaded onto an SDS–PAGE gel, and labeling on the pelleted samples was confirmed by scanning the gel using Typhoon FLA 7000 (GE Healthcare Life Sciences) with excitation and emission wavelengths of 400 and 505 nm, respectively. Alexa Fluor 488-labeled filaments/fibrils were then fragmented via sonication using a probe sonicator (Sonic Vibra Cell, Blanc Labo) for 1 s on/1 s off cycle for 5 s at 40% amplitude followed by one more time of sonication for 1 s on/1 s off cycle for 5 s at 20% amplitude. Alexa Fluor 488-labeled filaments/fibrils were then assessed by EM analysis and SDS–PAGE/fluorescence scanning.

### Primary neuron culture and treatment with TDP-43 filaments/fibrils

Primary cortical neurons were prepared from postnatal day 0 pups of wild-type mice (C57BL/6J RccHSD, Harlan) and cultured as previously described^66^. In brief, the cortex was isolated and cleaned in HBSS and was dissociated enzymatically (papain) at 37 °C for 30 min followed by gentle trituration in triturating media (MEM, 30% glucose, 50 mM l-glutamine and 10% horse serum). Cortical neurons were then resuspended in adhesion medium (MEM, 30% glucose, 50 mM l-glutamine, penicillin–streptomycin and 10% horse serum) plated on poly-d-lysine-coated glass coverslips on 24-well plates. After 3 h, adhesion medium was replaced with neuronal growth medium (Neurobasal medium, B27, 50 mM l-glutamine, and penicillin–streptomycin and incubated in an incubator with 37 °C and 5% CO_2_ for 7 d. On day 7, primary cortical neurons were treated with either PBS as a control or different TDP-43 preformed fibrils with a final concentration of 100 nM using Bioporter. After 3 d of treatment, cells were washed twice with PBS, and cells were incubated for 5 min with trypan blue (diluted at a 1:1 ratio with 1× PBS) to quench fluorescence from fibrils present outside the neuron. Cells were then fixed using 4% PFA for 15 min at room temperature and then co-stained together with anti-TDP-43 (1:500 dilution; 18280-1-AP) and MAP2 antibody (1:500 dilution).

### Statistics and reproducibility

The statistical analyses were carried out using KaleidaGraph (RRID: SCR_014980) or GraphPad Prism 9.1.1. The statistical tests were performed using ANOVA followed by Tukey’s multiple-comparisons test. Sample sizes (*n*) are indicated in the figure legends. The corresponding results of the statistics and the exact *P* values are provided in the figures legends of the respective experiments. All experiments using the recombinantly produced FL TDP-43, its variants and CP were carried out independently at least three times. No statistical methods were used to predetermine sample sizes but our sample sizes are similar to those reported in previous publications^39,42,66–68^. All data distribution was assumed to be normal, but this was not formally tested. No data were excluded from the analyses. Data collection and analysis were not randomized and were not performed blind to the conditions of the experiments.

### Reporting summary

Further information on research design is available in the Nature Portfolio Reporting Summary linked to this article.

## Online content

Any methods, additional references, Nature Portfolio reporting summaries, source data, extended data, supplementary information, acknowledgements, peer review information; details of author contributions and competing interests; and statements of data and code availability are available at 10.1038/s41593-023-01341-4.

## Supplementary information

Supplementary Information Supplementary Figs. 1–11, Supplementary Tables 1–6, Supplementary references and source data of gels and immunoblots for Supplementary Figs. 1, 2 and 3

Reporting Summary

Supplementary Data 1 Source data for Supplementary Figs. 5 and 6

## Data Availability

Raw cryo-EM images were deposited to the Electron Microscopy Public Image Archive (EMPIAR) under accession code EMPIAR-10840. Both cryo-EM maps of TDP-43 CP filaments were deposited to the Electron Microscopy Data Bank (EMDB) under accession code EMD-13795. The atomic model built was deposited to the PDB under accession code PDB 7Q3U. Source data are provided with this paper. All other data that are provided in the article and Supplementary Information are available from the corresponding author on reasonable request.

## Source data

Source Data Fig. 1 Statistical source data.
Source Data Fig. 1 Unprocessed gel.
Source Data Fig. 3 Statistical source data.
Source Data Fig. 4 Statistical source data.
Source Data Fig. 5 Statistical source data.
Source Data Fig. 6 Statistical source data.
Source Data Fig. 7 Statistical source data.
Source Data Extended Data Fig. 1 Statistical source data.
Source Data Extended Data Fig.2 Statistical source data.
Source Data Extended Data Fig. 4 Statistical source data.
Source Data Extended Data Fig. 7 Statistical source data.

## Code availability

No custom software codes were used.
