# Supplementary material for: Seeding the aggregation of TDP-43 requires post-fibrillization proteolytic cleavage
Source: Nat Neurosci. 2023 May 29;26(6):983–96. doi: 10.1038/s41593-023-01341-4 (PMC10244175; doi:10.1038/s41593-023-01341-4)

nature neuroscience

Article

https://doi.org/10.1038/s41593-023-01341-4

# Seeding the aggregation of TDP-43 requires post-fibrillization proteolytic cleavage

In the format provided by the
authors and unedited

# Seeding the aggregation of TDP-43 requires post-fibrillization proteolytic cleavage

## SUPPLEMENTARY DATA

Senthil T. Kumar[1], Sergey Nazarov[1], Sílvia Porta[2], Niran Maharjan[1], Urszula Cendrowska[1], Malek Kabani[1], Francesco Finamore[1], Yan Xu[2], Virginia M.-Y. Lee[2] & Hilal A. Lashuel*[1]

[1] Laboratory of Molecular and Chemical Biology of Neurodegeneration, Brain Mind Institute, EPFL, Switzerland

[2] Center for Neurodegenerative Disease Research (CNDR), Department of Pathology and Laboratory Medicine, University of Pennsylvania, Perelman School of Medicine, Philadelphia, PA, USA

*Corresponding author. Email: hilal.lashuel@epfl.ch

**This file includes:**

Supplementary Figs. 1- 11
Supplementary Tables 1-6
Supplementary references
Source data of gels and immunoblots for Supplementary Figs. 1, 2 and 3

## Supplementary figures

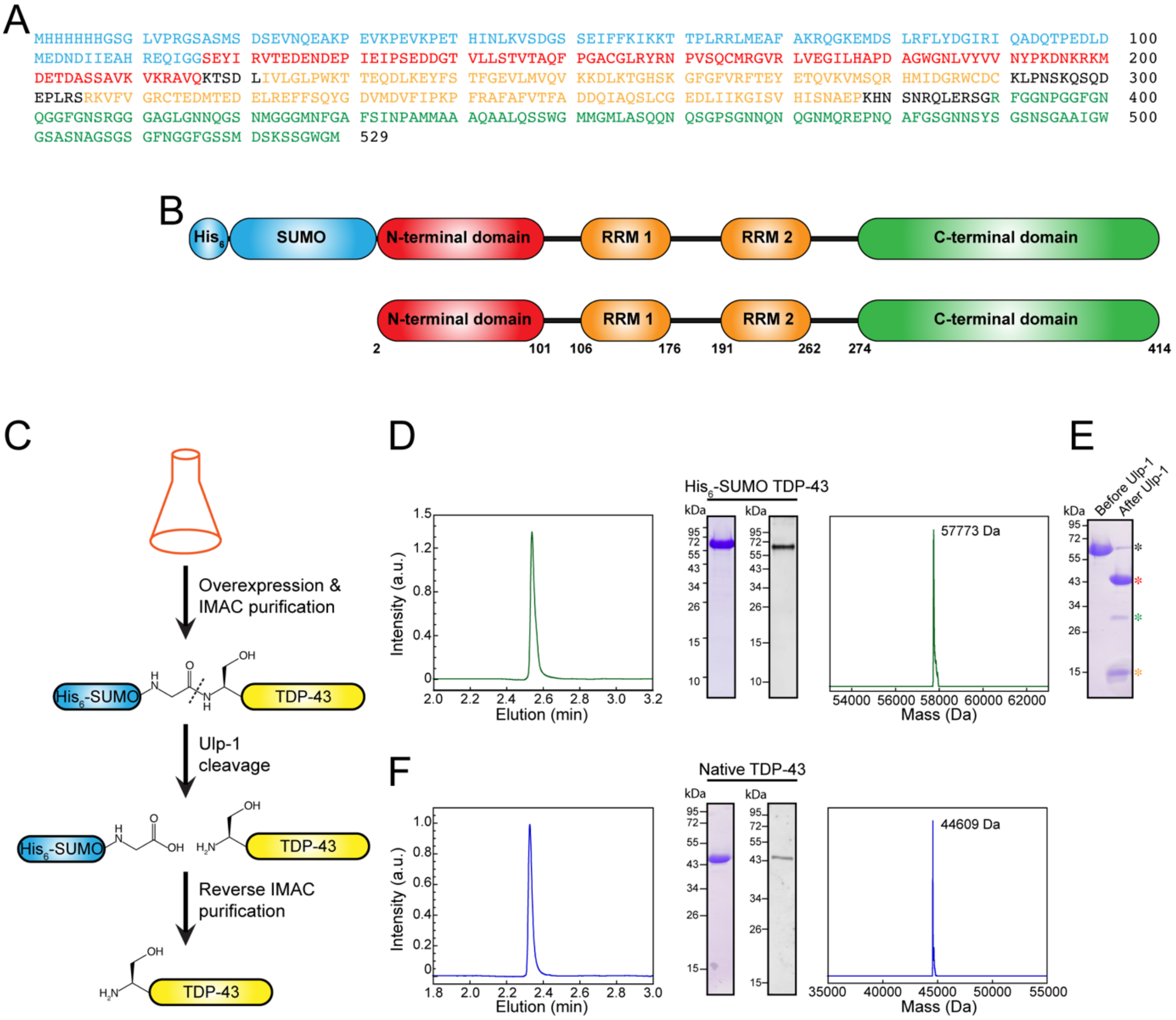


**Supplementary Figure 1.** Production of highly pure and tag-free TDP-43 from a SUMO-TDP-43 fusion protein. A) Amino acid sequence of His$_6$-SUMO-TDP-43. B) Schematic depiction highlighting the different domains of His$_6$-SUMO-TDP-43 and native TDP-43. Color coding is the same for the sequence (A) and schemes (B) with His$_6$-SUMO in blue, the N-terminal domain in red, RNA recognition motifs (RRM 1 and 2) in orange, the C-terminal region in green, and the loop regions in black. C) Schematic depiction of the design of a transient fusion strategy based on the fusion of the SUMO (Small Ubiquitin-like Modifier, yeast SMT3) protein with N-terminal His$_6$-tag to the full-length TDP-43, which involves two affinity purification (IMAC) steps. In the second step of reverse IMAC, because the Histidine tag is on the SUMO protein, cleavage of SUMO results in the generation of native TDP-43. D) Purity analysis of the SUMO-TDP-43 fusion protein by UPLC, Coomassie dye stained SDS-PAGE, Western blot analysis using TDP-43 (full length) antibody, and electron spray ionization-mass spectra. The theoretical molecular weight of His$_6$-sumo-TDP43 is 57775.2 Da. E) Coomassie dye stained SDS-PAGE of His$_6$-SUMO-TDP43 before and after overnight incubation of Ulp-1. Orange, green, red, and black asterisks show the positions of His$_6$-Sumo (13 kDa) Ulp-1 (27 kDa), native TDP-43 (44 kDa), and His$_6$-Sumo-TDP-43 (57 k Da), respectively. F) UPLC, Coomassie-stained SDS-PAGE, WB analysis, and ESI-MS show the purity of the native TDP-43. The theoretical molecular weight of native TDP-43 is 44608.4 Da. Using our purification

method, we obtained a high yield (˜15 mg/liter) of pure (> 98%) $His_6$-SUMO-TDP-43 after purification by IMAC affinity chromatography (from D and E).

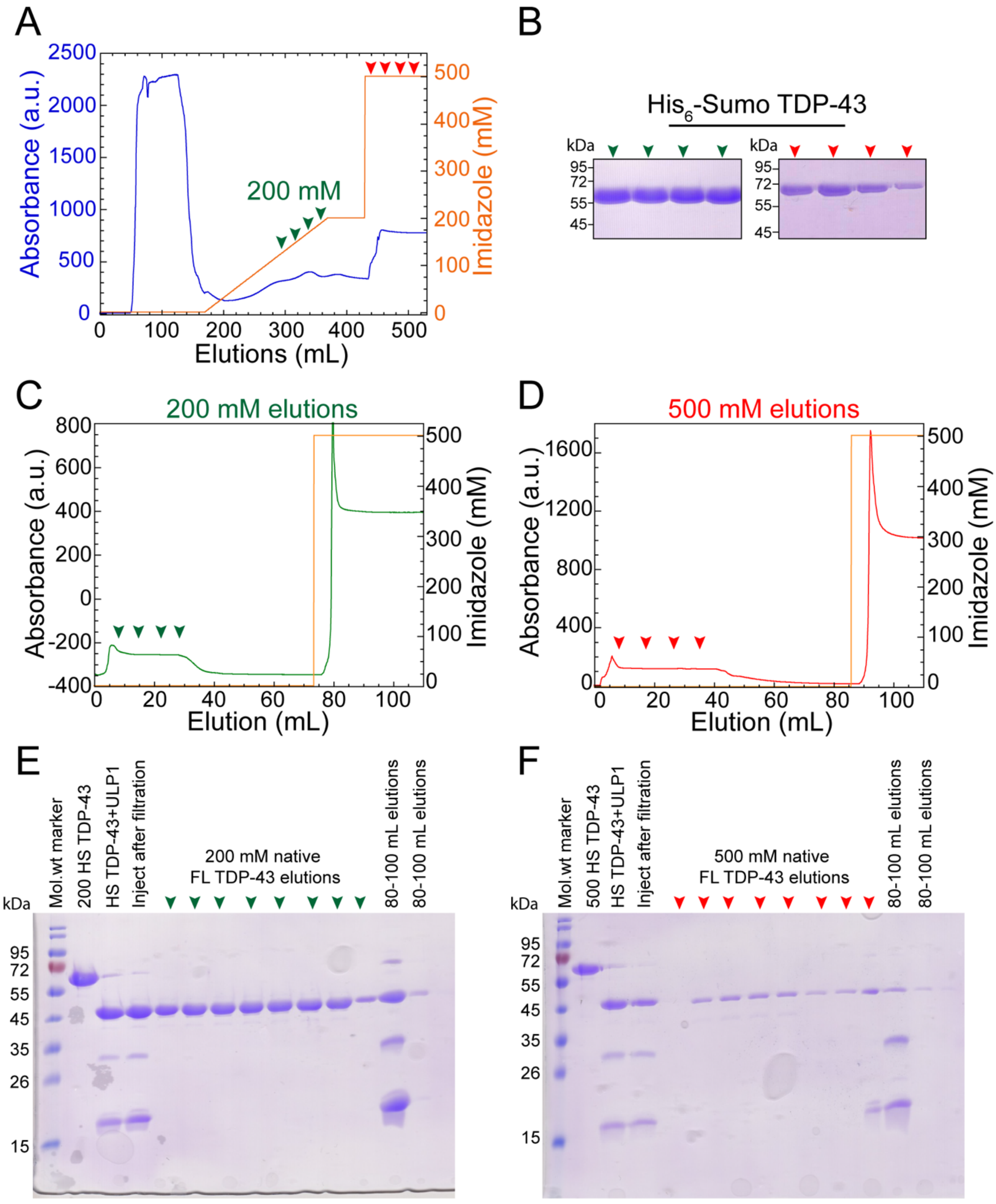


**Supplementary Figure 2.** Two major populations of pure $His_6$-SUMO-TDP-43 fractions; one eluted around 200 mM imidazole (200-TDP-43) and another population at 500 mM imidazole (500-TDP-43) concentrations. A) Affinity chromatogram of $His_6$-SUMO-TDP-43 showing the UV-absorbance as a blue curve and orange line as the linear increase in imidazole concentrations to elute TDP-43. Green and red arrowheads indicate the two populations of $His_6$-SUMO-TDP-43 eluting at 200 mM imidazole and 500 mM imidazole concentrations, respectively. B) Coomassie-stained denaturing PAGE of 200 mM imidazole (green arrowheads) and 500 mM imidazole (red arrowheads) elution fractions of His6-SUMO-TDP-

43. C and D) Reverse IMAC chromatogram of native TDP-43 purification at pH 7.0 showing the UV-absorbance as a green curve (C, 200-TDP-43) or red curve (D, 500-TDP-43) and an orange line as the step-wise imidazole concentrations to elute unbound native FL TDP-43 (green (C) and red (D) arrowheads) and His-SUMO or Ulp-1 in the 100% buffer B. E) SDS-PAGE analysis of fractions eluted from the reverse IMAC of native TDP-43 eluted at 200 mM imidazole concentrations during His-Sumo-TDP-43 purifications and F) from 500 mM imidazole concentrations.

The idea of the reverse IMAC strategy is that the native form of FL TDP-43 from Ulp-1 cleaved mixture, devoid of $His_6$-tag, elutes with the sample injection without binding to the column. Reducing the pH of reverse IMAC buffers from 8.0 to 7.0 changes the net charge of TDP-43 from -6.1 to -3.0, respectively. At pH 8.0, full-length TDP-43 was binding tightly to the IMAC column even without $His_6$-tag and hindered its separation from other $His_6$-tag proteins. However, changing the pH to 7.0 eluted the full-length TDP-43 in a high quantity, and more importantly with high purity, which was determined using SDS-PAGE and WB analysis (Fig. S1F). Mass spectrometry determined the molecular weight of the full-length TDP-43 protein at 44609 Da (Fig. S1F) which matched very well with the theoretical mass calculated from the sequence (44608.4 Da).

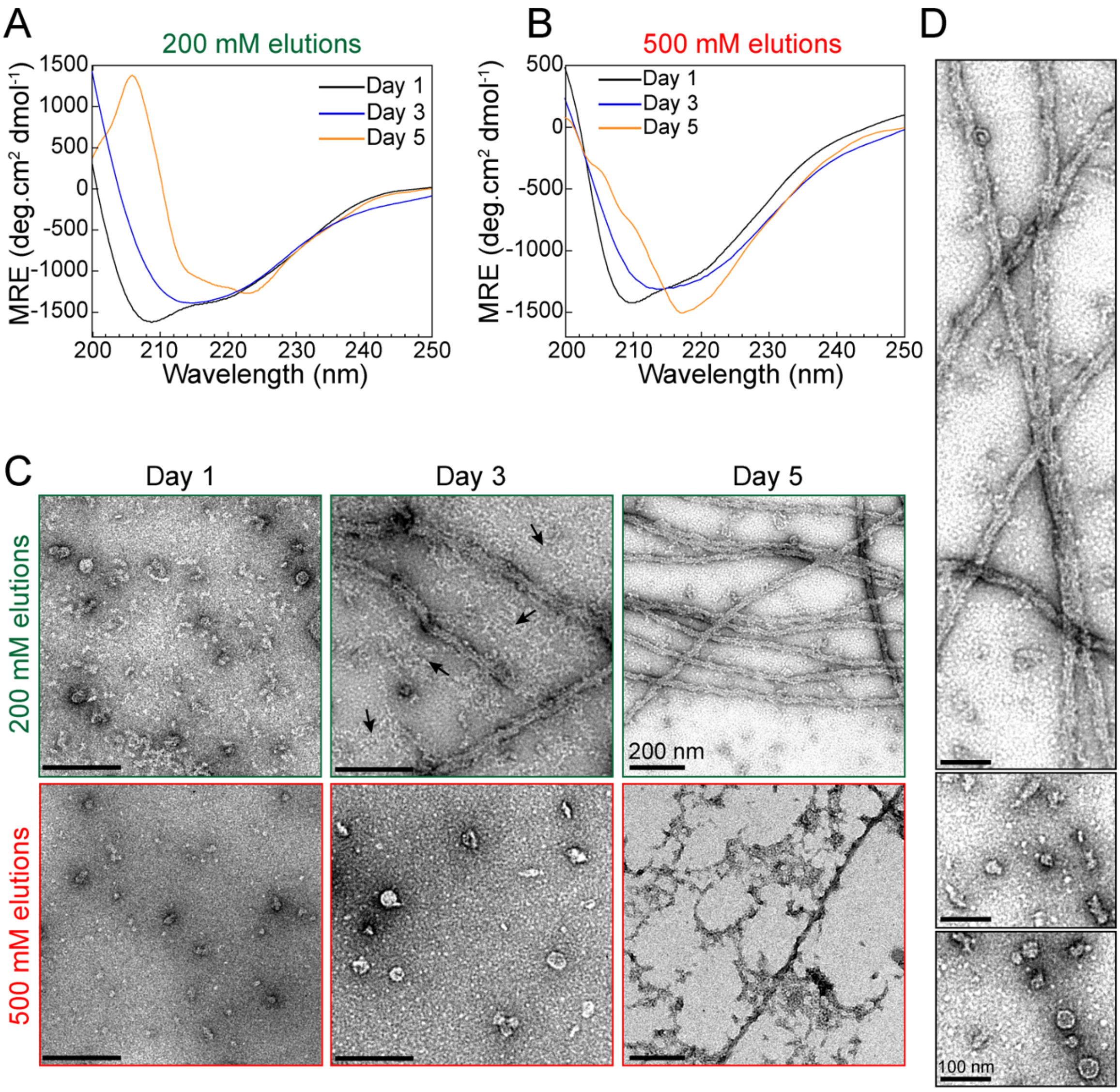


**Supplementary Figure 3.** Two distinct species of FL TDP-43 (200-TDP-43 and 500-TDP-43), after removal of the His$_6$-SUMO protein, exhibit different aggregation properties. A and B) Far UV circular dichroism spectra of native TDP-43 fibrillation eluted at 200 mM imidazole (A) and 500 mM imidazole (B) on day 1 (black), day 3 (blue), and day 5 (yellow). C) EM images of 200 mM imidazole (green) and 500 mM imidazole (red) eluted native TDP-43 after day 1, day 3, and day 5. Black arrows on the day 3 image show the presence of protofibril-like structures. D) EM images of 200 mM imidazole eluted TDP-43 after day 5 show the presence of filaments and oligomers in the same sample.

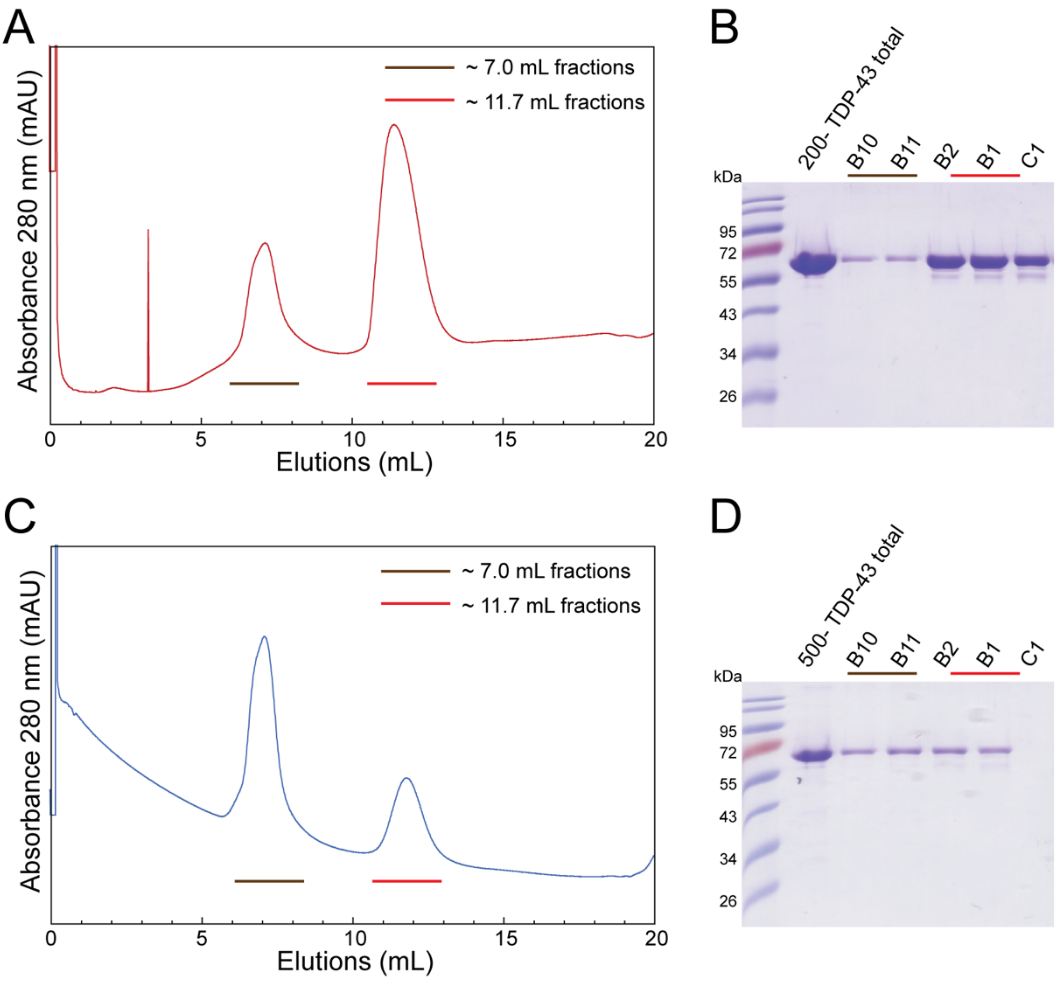


**Supplementary Figure 4.** A and B) Size exclusion analysis of 200 mM fraction of His-SUMO-TDP-43 (A) and SDS-PAGE analysis (B). C and D) Size exclusion analysis of 500 mM fraction of His-SUMO-TDP-43 (C) and (D) SDS-PAGE analysis. SEC analysis of the freshly purified IMAC samples showed that the majority of the 200-TDP-43 fractions elute as monomers (>80%) (Fig. S4A and B), whereas 500-TDP-43 fractions contain >50% as oligomers (Fig. S4C and D). After removal of the $His_6$-SUMO tag, FL TDP-43 from 200 TDP-43 and 500-TDP-43 fractions was purified (Figs. S2C-D). The aggregation of 200-TDP-43 and 500-TDP-43 proteins at 37°C was monitored over time by circular dichroism spectroscopy (CD) and analyzed by electron microscopy (EM). At day 1, both 200-TDP-43 and 500-TDP-43 proteins exhibited identical CD spectra possessing two minima (~210 nm and ~ 222 nm) consistent with predominantly α-helical structures (Fig. S3A-B). Interestingly, on days 3 and 5, both 200-TDP-43 and 500-TDP-43 exhibited a spectral shift from double minima to a broader minima center around 222 nm (Fig. S3A-B), suggesting a transition from α-helices to β-sheet-rich secondary structures. Despite the similar CD spectra between 200-TDP-43 and 500-TDP-43 samples, EM analysis revealed striking morphological differences over time. EM analysis revealed the presence of ring/pore-like structures with an average diameter of 36 ± 12 nm in both samples on day 1 (Fig. S3C, day 1). However, exclusively 200-TDP-43 showed also tubular oligomers with an average diameter of 20 nm (Fig. S3C, day 1). On day 3, the 200-TDP-43 samples showed long filamentous species and short protofibril-like curvilinear aggregates, which seem to disappear over time (Fig. S3C, day 3 black arrows). At 5 days, the filamentous species with an average diameter of ~22 nm were observed as the major species (Fig. S3C-D, days 3 and 5). In contrast, the 500-TDP-43 samples showed mainly

heterogeneous oligomers and amorphous aggregates and only a very few occurrences of filamentous-shaped structures similar to those observed in the 200-TDP-43 sample even after a longer period of incubation (Fig. S3C). The filamentous structures formed in the 200-TDP-43 sample resemble the filamentous structures observed in brain-derived TDP-43 extracted from ALS and FTLD-TDP cases (Table S1).

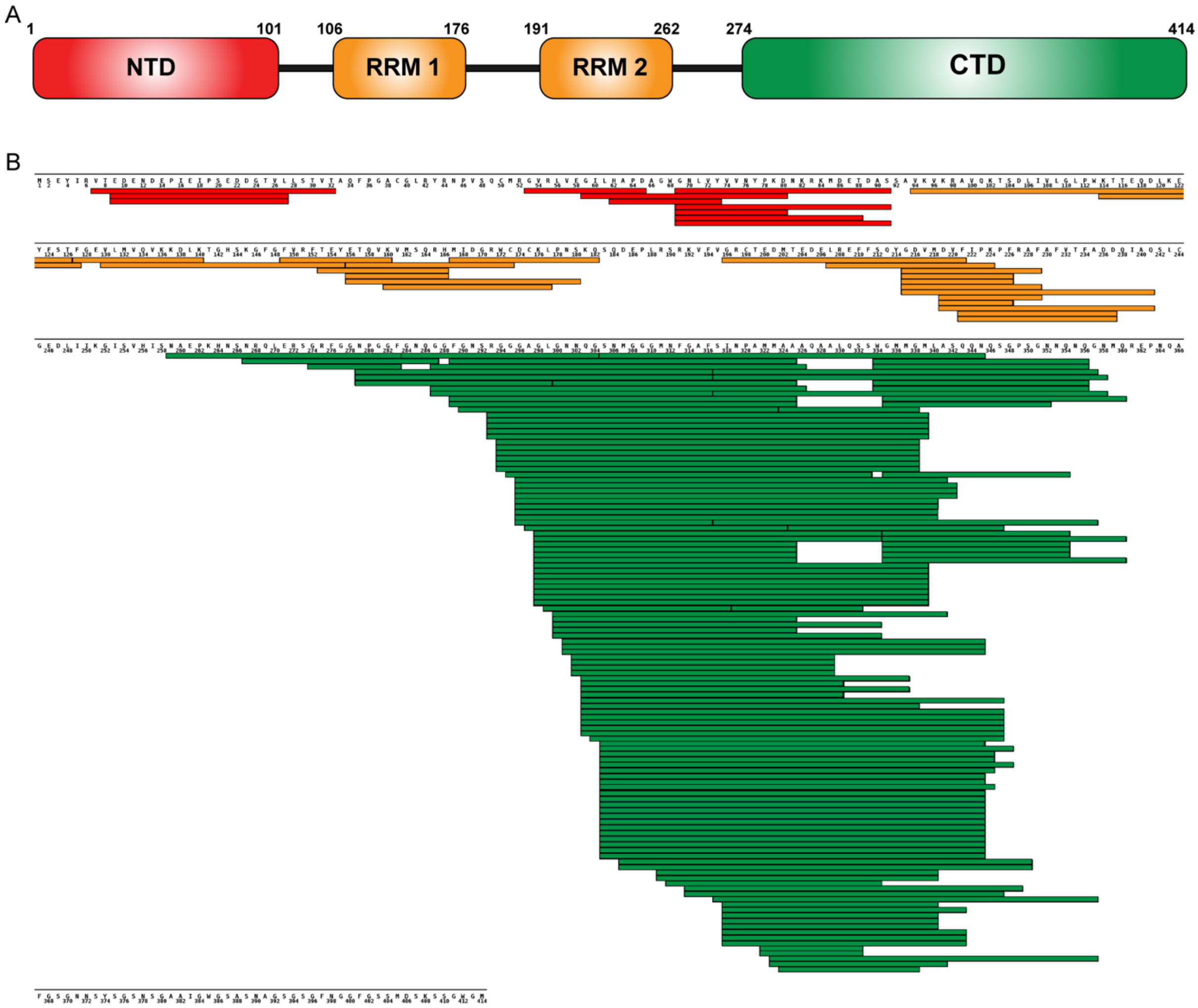


**Supplementary Figure 5.** A) Schematic depiction highlighting the different domains of native TDP-43. Color coding is the N-terminal domain in red (NTD), RNA recognition motifs (RRM 1 and 2) in orange, the C-terminal region (CTD) in green, and loop regions in black. B) Distribution of peptides identified using LC/MS analysis following the PK digestion of TDP-43 filaments. Red, orange, and green bars represent the peptides from the region of NTD, RRMs, and CTD, respectively.

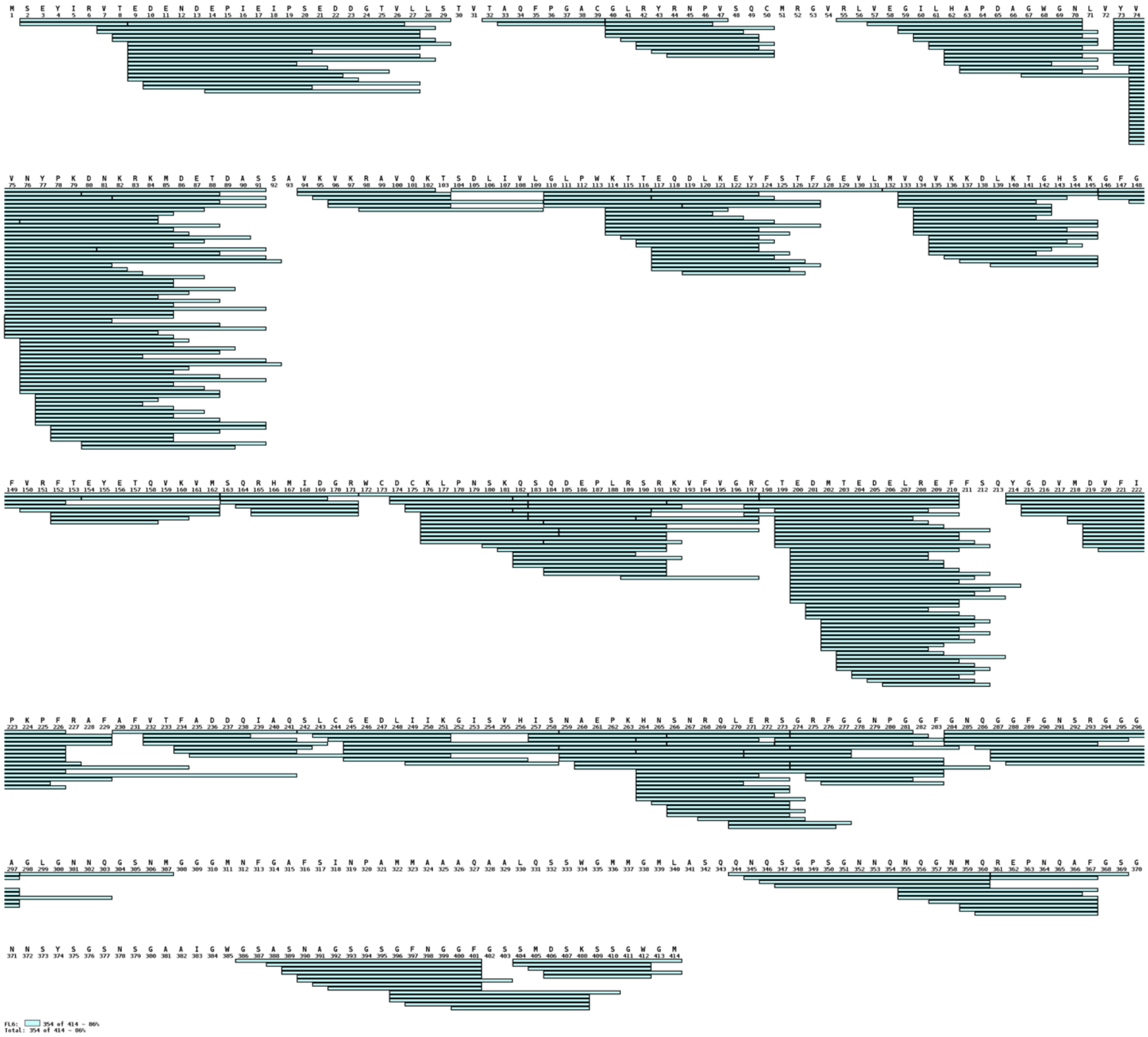

**Supplementary Figure 6.** Distribution of peptides identified using LC/MS analysis following the PK digestion of non-fibrillar TDP-43.

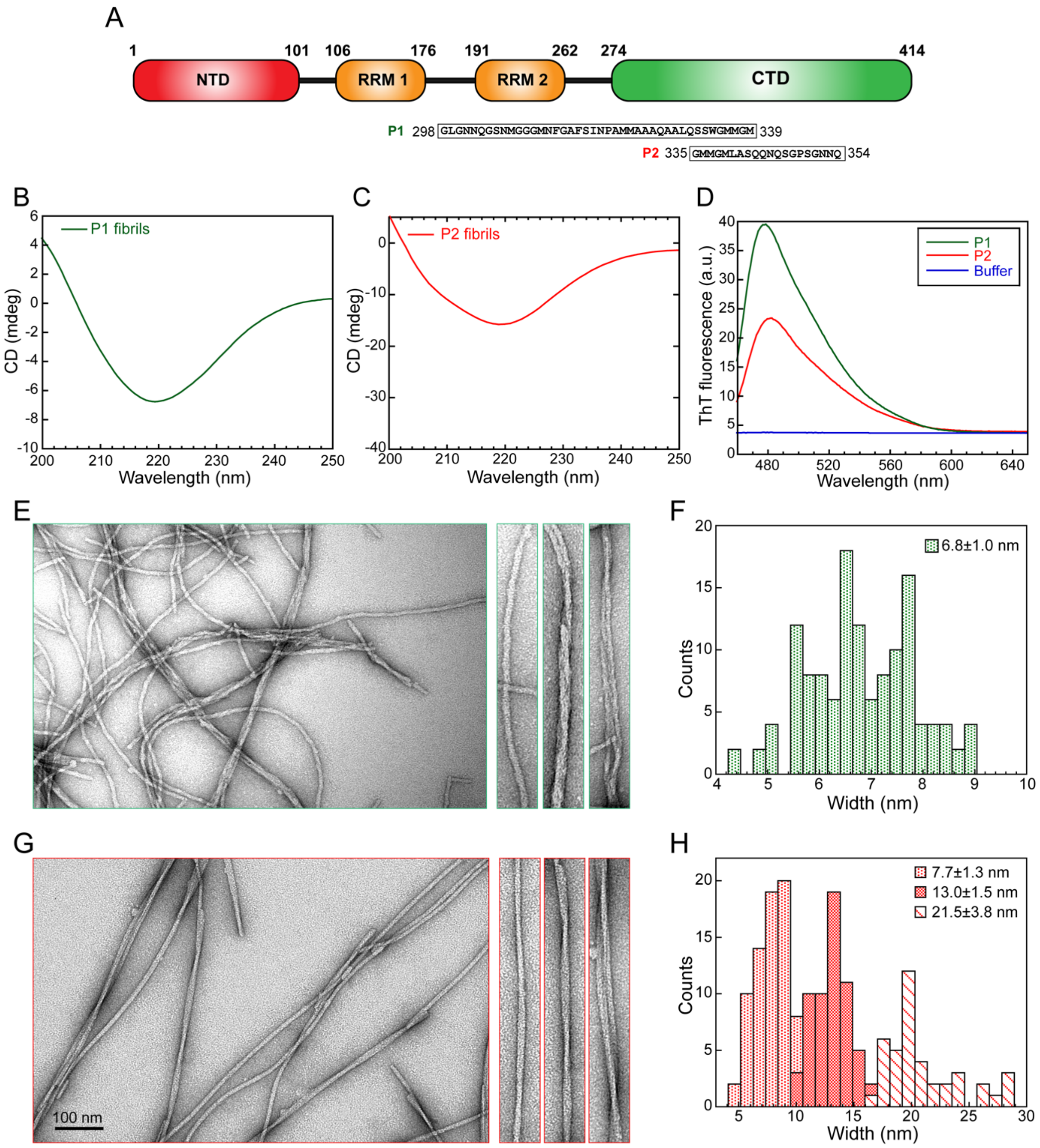


**Supplementary Figure 7.** Structural characterization of fibrillation of P1 (298-339) and P2 (334-354) peptides. A) Schematic of regions and their amino acids of peptides P1 and P2 covering the FL TDP-43. B and C) CD spectra of P1 and P2 fibrils, respectively. D) ThT fluorescence spectra of P1 and P2 fibrils. E) Representative EM image of P1 fibrils and their (F) width analysis. G Representative EM image of P2 fibrils and their (H) width analysis.

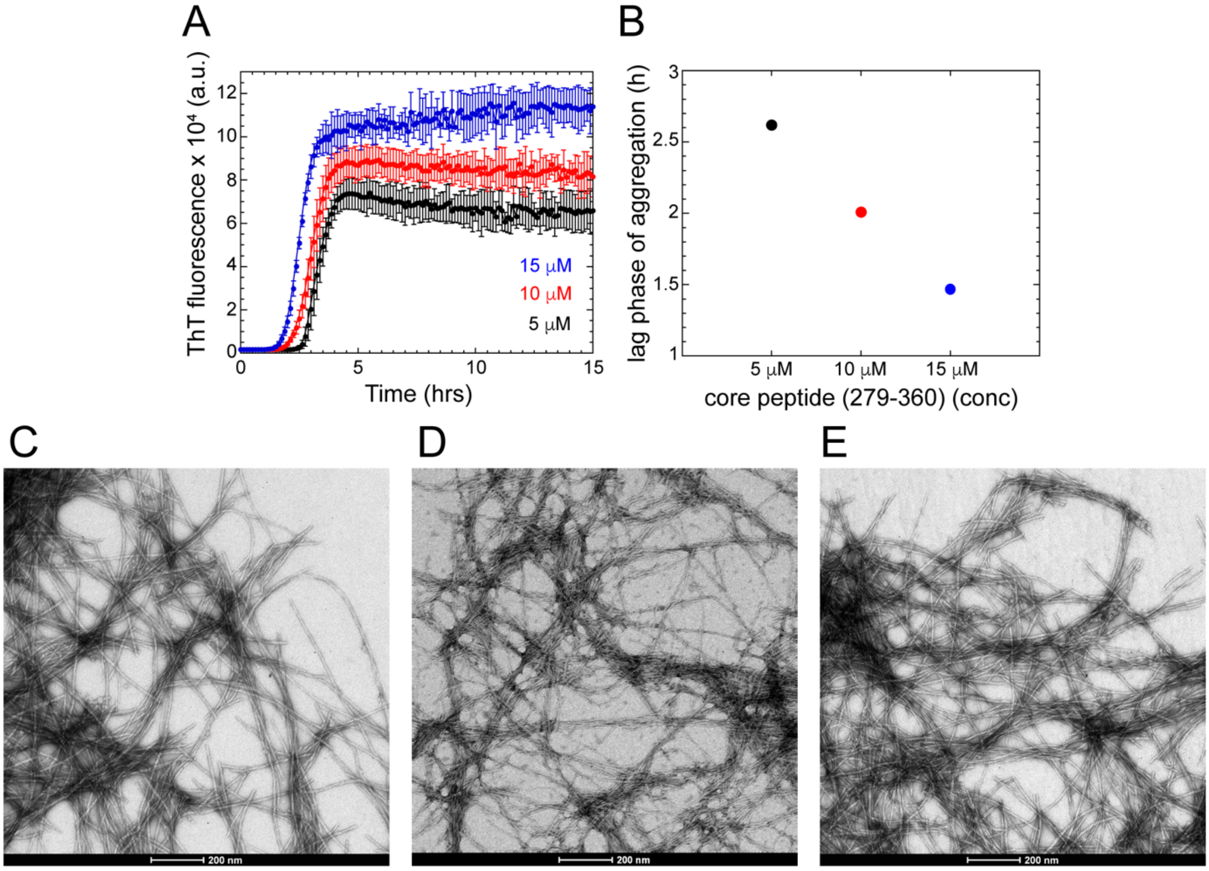


**Supplementary Figure 8.** Optimization and development of conditions to obtain reproducible ThT-based aggregation kinetics for the CP A) ThT fluorescence monitored aggregation kinetics of recombinant CP at varying initial concentrations at 5 µM, 10 µM, and 15 µM. Averages of three traces are shown as solid lines in all samples. All figures show ThT intensity as a function of time (non-normalized raw data). Error bars represent SD. B) Measurement of the lag phase of all the samples from (A). C-E) Representative electron micrographs of CP fibrils at varying initial concentrations at 5 µM (C), 10 µM (D), and 15 µM (E).

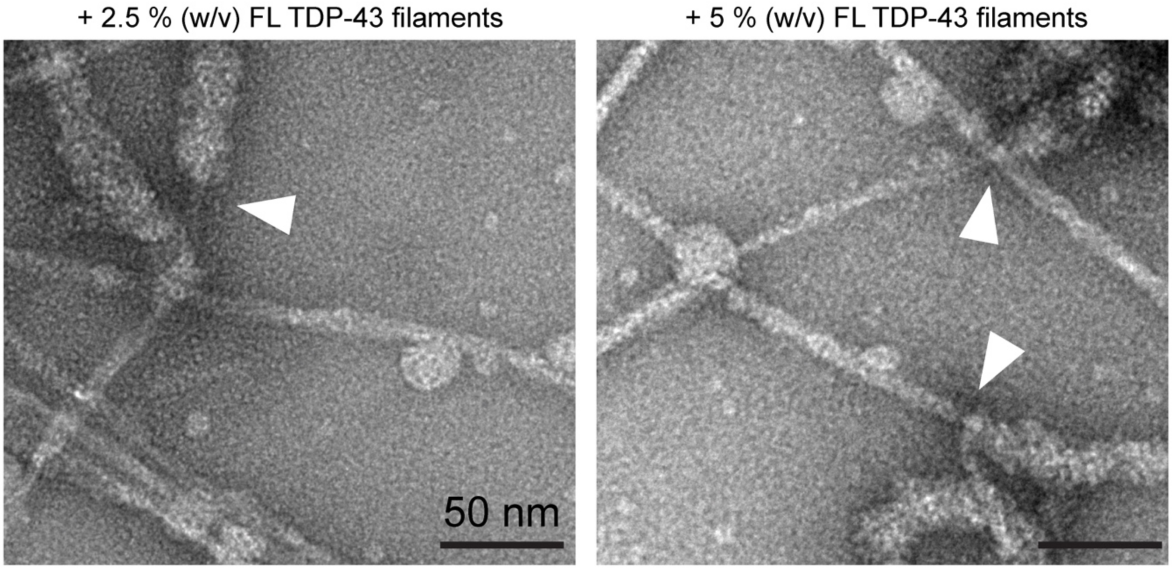


**Supplementary Figure 9.** EM analysis at the CP aggregation endpoint (Fig. 6C) showed the formation of CP fibril morphologies seeded with 2.5% and 5.0% (w/v) FL TDP-43 filaments. White arrowheads on the 2.5% and 5.0% FL TDP-43 filaments seeded EM images show the edges of a few FL seeds from which CP fibrils are growing. Scalebars=50 nm

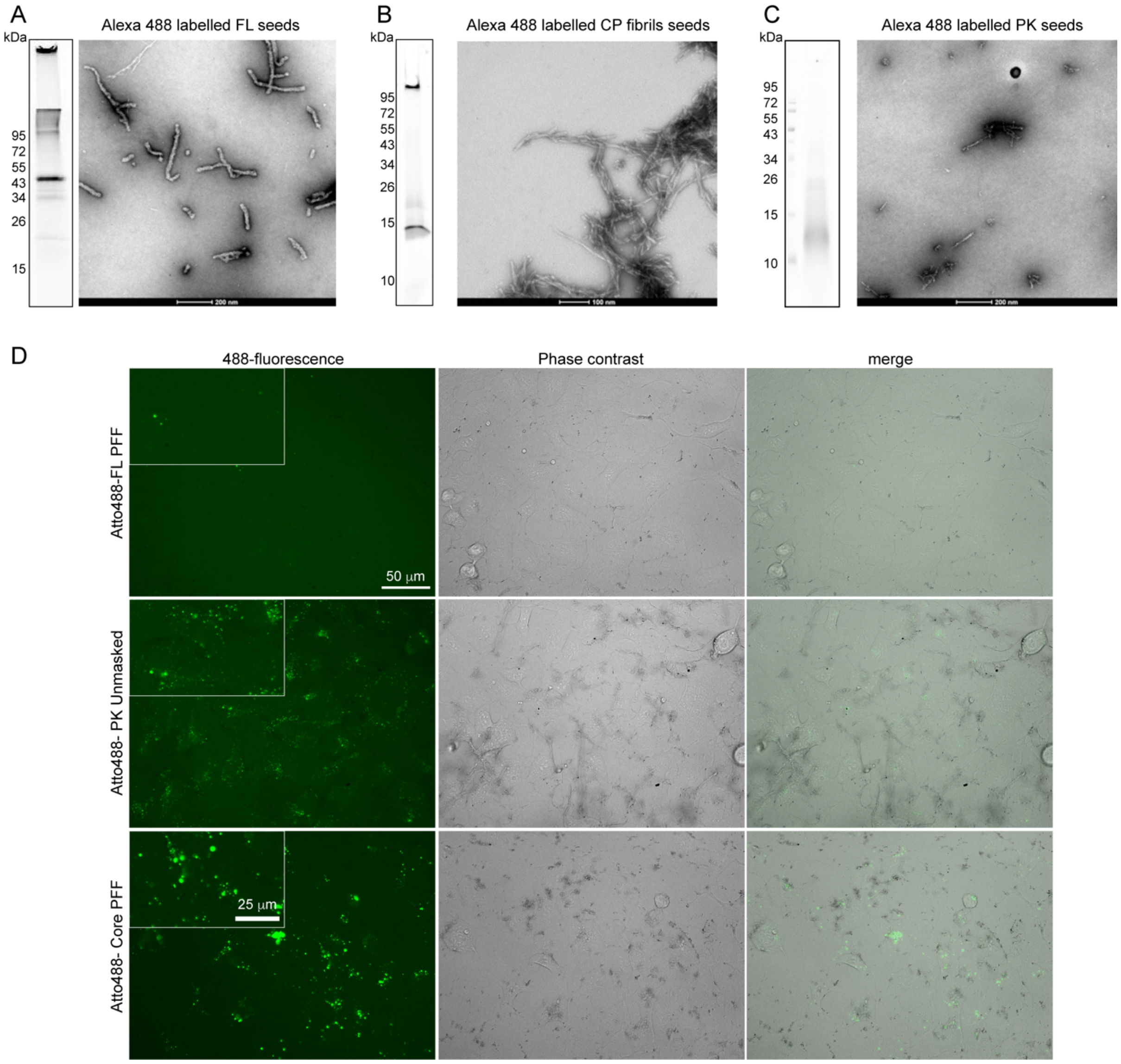


**Supplementary Figure 10.** A-C) Fluorescence images and representative electron micrographs of different kinds of Alexa 488 fluorescently labeled seeds of A) FL TDP-43 filaments seeds (FL seeds) B) core peptide fibril seeds (core seeds), and C) seeds from PK-unmasked FL TDP-43 filaments (PK seeds). D) Uptake of Atto-488 labeled FL seeds, core seeds, and PK seeds on QBI-293 cells. White boxes in 488-fluorescence panel shows the magnified view of 488-fluorescence images. The experiment was repeated at least 3 times.

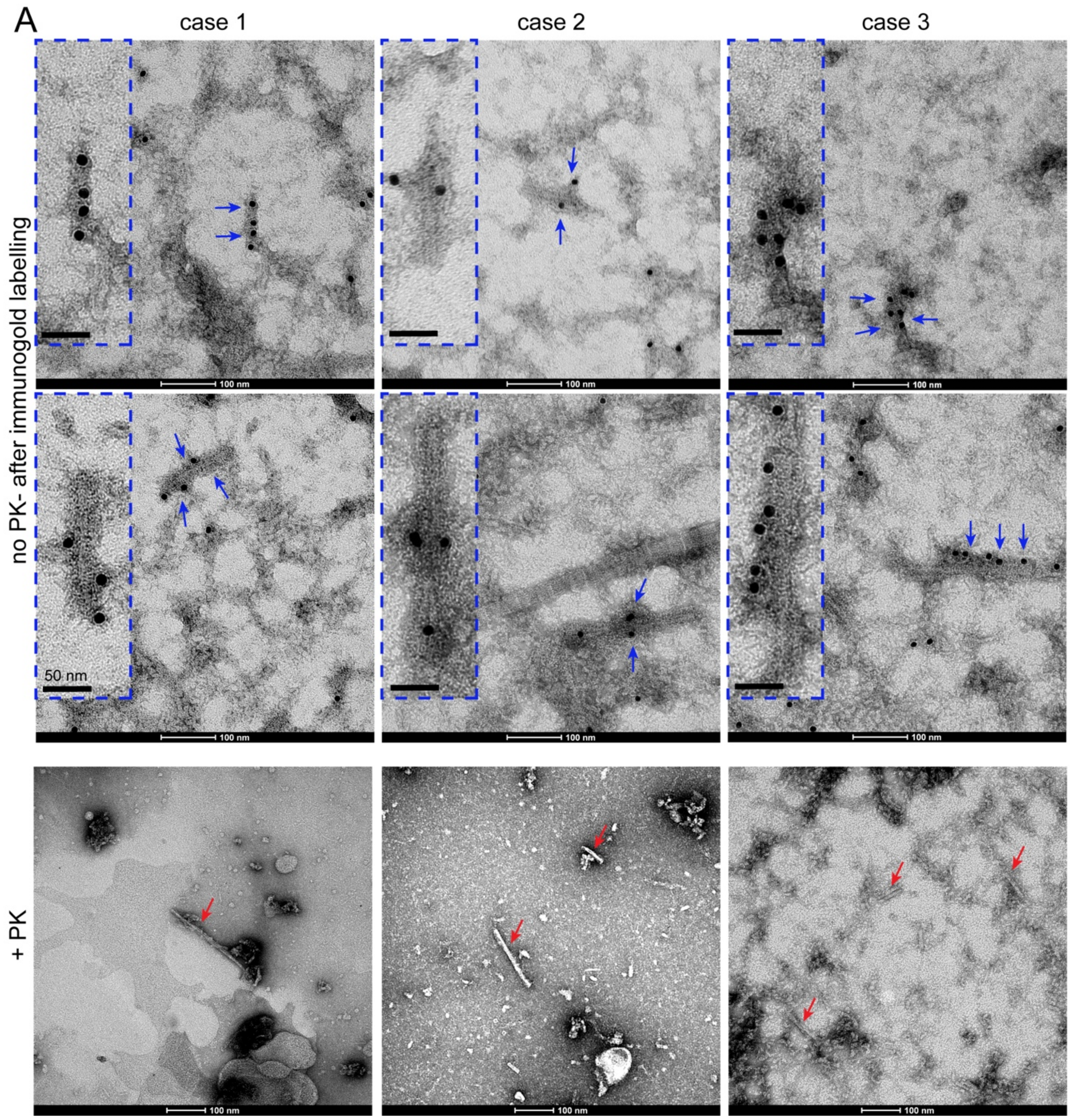


**Supplementary Figure 11.** A) EM images of sarkosyl-insoluble extracts from the different ALS/FTLD-TDP cases before and after PK treatment. Immunogold labeling using TDP-43 antibody (epitope: 394-414) followed by EM analysis was carried out for ALS/FTLD-TDP cases before PK treatment. Blue arrows in all cases (before PK treatment) point to the immunogold-positive TDP-43 filament structures. Blue arrows pointed structures are magnified and shown in the blue boxes within the same EM images. Red arrows point to the filament structures after PK treatment.

## Supplementary Tables

## **Supplementary Table 1:** Structural characteristics of TDP-43 aggregates *in vivo*

| Tissue details | Amyloid characteristics | Aggregate characteristics | Biochemistry of the TDP-43 aggregates | Reference |
|---|---|---|---|---|
| • Spinal cord motor neuron inclusions of an ALS patient | • Unknown. No amyloid dye binding studies carried out | • Abnormal fibers of 15 nm diameter. | • TDP-43 at 45kDa, with additional 18 to 26 kDa fragments in the sarkosyl-insoluble fractions | [1] |
| • NCI and NII in FTLD-U | • Unknown. No amyloid dye binding studies carried out | • Filaments had a diameter of 10–17 nm, with thickness increasing to 20 nm or more in regions | • No biochemistry analysis | [2] |
| • ALS spinal cord<br>• Hippocampal TDP-43 inclusions | • Has amyloid properties binds ThS<br>• Lacks amyloid properties (granular appearance) | • Skein like inclusions has 10- to 20-nm-diameter straight filaments<br>• Granular shaped in nature | • No biochemistry analysis | [3] |
| • FTLD with NCIs, DNs, and NIIs | • Unknown. No amyloid dye binding studies carried out | • NCI, 9 nm (4-16 nm); DN, 10 nm (5-16 nm); NII, 18 nm (9-50 nm) | • No biochemistry analysis | [4] |
| • FTLD-U | • Has amyloid properties binds ThS | • Filaments appearing straight and had a diameter ranging from 15 to 20 nm | • 45 and 25 kDa of TDP-43 | [5] |
| • ALS and FTLD sub types | • Unknown. No amyloid dye binding studies carried out | • Filaments that are 5–8 nm thick and 30–50 nm long | • Prominent 45 and 25 kDa and lower molecular weight bands of TDP-43 | [6] |
| • FTLD-TDP sub types | • Unknown. No amyloid dye binding studies carried out | • Not described | • No biochemistry analysis | [7] |
| • FTLD-TDP cases | • Unknown. No amyloid dye binding studies carried out | • A wide range of granular filamentous aggregates from 4- 50 nm in diameter in the NCIs, DNs and NIIs | • No biochemistry analysis | [8] |

*Abbreviations: ALS: Amyotrophic lateral sclerosis, FTLD-U: Frontotemporal lobar degeneration with ubiquitin-positive inclusions, FTLD-TDP: Frontotemporal lobar degeneration with TDP-43 inclusions, NCIs: neuronal cytoplasmic inclusions, DNs: dystrophic neurites, NIIs: neuronal intranuclear inclusions, ThS: thioflavin S, kDa: kilodalton, nm: nanometres.*

**Supplementary Table 2:** Recombinant forms of TDP-43, their purification conditions and structural characteristics of FL TDP-43 fibrils *in vitro*

| TDP-43 | TDP-43 purification method | Fibrillation condition | Fibril characteristics | Reference |
|---|---|---|---|---|
| TDP-43 | Purified after ammonium sulfate precipitation as a soluble species | Phosphorylation of TDP-43 using casein kinase II and fibrillation under shaking conditions | • Mixtures of single, double and triple filamentous structures of TDP-43 with 10–12 nm diameter containing double filamets being the major form.<br>• Weakly ThS positive | [8] |
| $His_6$-TDP43 | Purified from soluble material | Fibrillation for 0–120 min with agitation at 1400 rpm at 25 °C | • Less filamentous to more amorphous appearance. Granulo-filamentous<br>• No ThT binding<br>• 18-20 nm of width of filaments | [9] |
| $His_6$-TDP43 | Purified from inclusion bodies and refolded as soluble species | Fibrillation under high shaking condition | • Thin fibril structures with 3.0 nm of diameter.<br>• Bundle of thinner fibrils.<br>• Minimally ThT positive | [10] |
| His-Sumo-TDP-43 | Purified from soluble material | Fibrillation under continuous shaking condition at 37 C | • No EM data on the TDP-43 fibrils alone<br>• ThT negative | [11] |
| GST-TEV-TDP-43 | Purified from soluble material | Addition of TEV protease to the fibril assembly buffer and agitation at 700rpm for 90 min at 25°C | • EM analysis but no details/explanation on the diameter/width of the fibril and its morphological characteristics | [12] |
| TDP-43 | Purified from inclusion bodies and refolded as soluble species | Fibrillation for for 1 week at room temperature in the buffer 20 mm MES buffer pH 7.5 | • Thin, short fibrils with ~ 5–10 nm of diameter<br>• No ThT/S binding studies<br>• Typical amyloid specific XRD diffraction | [13] |
| PelB-FL TDP-43-$His_6$ (~48.6 kDa) | Purified from inclusion bodies and refolded as soluble species | Fibrillation under dialysis condition in 50 mM NaH2PO4, 1 mM DTT, pH 7.4 | • Filamentous structures with 35±5 nm diameter.<br>• No ThT binding and absence of b-sheet structures | [14] |
| SUMO-TDP-43 | Purified from soluble material | Not given | • highly clumped fibril-like structures and disordered aggregates | [15] |
| SUMO-TDP-43 | Purified from soluble material | No fibrillation carried out | • No fibrillation carried out | [16] |
| $His_6$-SUMO-TDP-43 | Purified from soluble material | Addition of Ulp-1 to assay condition to remove $His_6$-SUMO and to initiate aggregation in a 96-well plate at 30 °C under shaking | • formation of granulo-filamentous aggregates | [17] |

To understand the amyloid nature of the TDP-43 fibrils, different attempts were explored to recombinantly purify TDP-43 in its full-length form and several methods were generated for the preparation of fibrils [9-18]. While many studies report the total absence of ThT binding, only a few show minimal/weak ThT binding by the TDP-43 fibrils. Not surprisingly, the fibril morphologies from the FL TDP-43 also varied between studies in the differences in the width of the fibrils ranging between 3 and 35 nm. These discrepancies could be attributed to differences in the purification protocols, an additional tag on the FL TDP-43, and fibrillation conditions. Another limitation of previous studies is that they relied on constructs containing non-native sequences and the use of purification conditions that required denaturing and refolding the protein.

**Supplementary Table 3:** Demographic data of the ALS/FTLD-TDP cases and neurologically normal individuals used in this study

| Case ID# | Sex | Age on onset | Age of death | Genetics | Clinical Dx | NPDx | PMI (hrs) |
|---|---|---|---|---|---|---|---|
| 1 | M | 46 | 48 | *C9orf72 expansion* | ALS<br>FTD-NOS | FTLD-TDP/MND | 12.5 |
| 2 | M | 55 | 74 | - | bvFTD | FTLD-TDP<br>Argyrophilic grain disease<br>Hippocampal Sclerosis | 19 |
| 3 | M | N/A | 75 | *TBK1* | Corticobasal syndrome | FTLD-TDP<br>AD (low) | 10 |
| 4 | F | - | 60 | - | Neurologically normal | NL | 9 |
| 5 | M | - | 72 | - | Neurologically normal | PART | 17 |

*Abbreviations: N/A- not available, TBK1- TANK-binding kinase 1, FTD- frontotemporal degeneration, FTLD-NOS- FTD not otherwise specified, ALS- amyotrophic lateral sclerosis, bvFTD- behavioral variant frontotemporal degeneration, MND- motor neuron disease AD- Alzheimer's Disease, Clinical Dx- Clinical diagnosis, NPDx- Neuropathological diagnosis, PMI- post mortem interval, NL: no lesions, PART- Primary age-related tauopathy*

**Supplementary Table 4.** ELISA measures of TDP-43 protein content and BCA measures in human brain-derived sarkosyl-insoluble extracts used in the study.

| | **ELISA C89*** **(ng TDP-43/ml)** | **ELISA N65*** **(ng TDP-43/ml)** | **BCA (mg/ml)** | **TDP-43 (Elisa c89)*/total protein** **TDP-43 (Elisa N65)*/total protein** **(%)** |
|---|---|---|---|---|
| 1 | 473 | 223 | 3.2 | 0.015-0.007 |
| 2 | 681 | 569 | 1.4 | 0.048-0.04 |
| 3 | 418 | 226 | 1.3 | 0.032-0.017 |

** ELISA measure of TDP-43 content using a C-terminus (C89) or N-terminal (N65) anti-TDP-43 antibody as a reporter* [18]

**Supplementary Table 5:** List of reported shorter peptides from TDP-43, their methods of identification, and structural characteristics of their fibrils *in vitro*

| Peptides | Width | Morphological characteristics | Method of identification of peptide | References |
|---|---|---|---|---|
| 287-322 | ~11 nm | Straight or gently curved with periodicities | PONDR bioinformatic program | [19] |
| 274–313, 314-353 | 10-15 nm | Long, straight or twisted fibrils | Deletion mutants on cell models | [20] |
| 286-331 | 0.29 nm | Straight looking fibrils but no explanation on periodicities | MD simulation and protscale server | [21] |
| 341-357 | 15−25 nm | No description of morphologies | MD simulation | [22] |
| 246–258, 311-320 | 7-12 nm | Dense, long filamentous fibrils | Series of 13-mer peptides spanning the TDP-43 (220-414) | [23] |
| 311-360, 318-342 | No description | Abundant short filaments within 12 h incubation but no description of morphologies | CTD deletion mutants on cell models | [24] |

*Abbreviations: PONDR: Predictor of Natural Disordered Regions, MD: molecular dynamics.*
*ProtScale: https://web.expasy.org/protscale/*

**Supplementary Table 6:** Cryo-EM data acquisition and structure determination

| | EMDB-13795, PDB-7q3u | |
|---|---|---|
| **Data collection and processing** | | |
| | **Two protofilaments** | **One protofilament** |
| Magnification | 80000 | |
| Voltage (kV) | 300 | |
| Electron exposure (e–/Å$^2$) | 40 | |
| Defocus range (μm) | -0.9 to -2.0 | |
| Pixel size (Å) | 1.06 | |
| Symmetry imposed | Helical, rise=4.83 Å, twist= -3.09° | Helical, rise=4.84 Å, twist= -3.11° |
| Initial particle images (no.) | ~1,000,000 helical segments | |
| Final particle images (no.) | 5795 | 8104 |
| Map resolution (Å) $FSC_{0.143}$ | 3.7 | 4.1 |
| | | |
| **Model** | | |
| Composition (#) | | |
| Chains | 5 | |
| Atoms | 2355 (Hydrogens: 0) | |
| Residues | Protein: 355<br>Nucleotide: 0 | |
| Water | 0 | |
| Ligands | 0 | |
| Bonds (RMSD) | | |
| Length (Å) (# > 4σ) | 0.006 (0) | |
| Angles (°) (# > 4σ) | 1.367 (0) | |
| MolProbity score | 2.37 | |
| Clash score | 17.82 | |
| Ramachandran plot (%) | | |
| Outliers | 0 | |
| Allowed | 10.72 | |
| Favored | 89.28 | |
| Rama-Z (Ramachandran plot Z-score, RMSD) | | |
| whole (N = 345) | -5.79 (0.20) | |
| Rotamer outliers (%) | 0 | |
| Cβ outliers (%) | 0 | |
| Peptide plane (%) | | |
| Cis proline/general | 0.0/0.0 | |
| Twisted proline/general | 0.0/0.0 | |
| CaBLAM outliers (%) | 10.45 | |
| ADP (B-factors) | | |

| | | |
|---|---|---|
| Iso/Aniso (#) | 2355/0 | |
| min/max/mean | | |
| Protein | 54.85/128.31/95.59 | |
| Nucleotide | --- | |
| Ligand | --- | |
| Water | --- | |
| Occupancy | | |
| Mean | 1 | |
| occ = 1 (%) | 100 | |
| 0 < occ < 1 (%) | 0 | |
| occ > 1 (%) | 0 | |
| | | |
| **Data** | | |
| Supplied Resolution (Å) | 3.7 | |
| Resolution Estimates (Å) | Masked Unmasked | |
| d FSC (half maps; 0.143) | --- --- | |
| d 99 (full/half1/half2) | 3.4/---/--- 3.4/---/--- | |
| d model | 4.2 4.1 | |
| d FSC model (0/0.143/0.5) | 3.4/3.8/15.6 3.4/3.9/15.6 | |
| Map min/max/mean | -0.08/0.08/0.00 | |
| | | |
| Model vs. Data | | |
| CC (mask) | 0.47 | |
| CC (box) | 0.23 | |
| CC (peaks) | 0.03 | |
| CC (volume) | 0.46 | |
| Mean CC for ligands | --- | |

**References:**


1 Hasegawa, M. *et al.* Phosphorylated TDP-43 in frontotemporal lobar degeneration and amyotrophic lateral sclerosis. *Ann Neurol* **64**, 60-70, doi:10.1002/ana.21425 (2008).

2 Lin, W. L. & Dickson, D. W. Ultrastructural localization of TDP-43 in filamentous neuronal inclusions in various neurodegenerative diseases. *Acta Neuropathol* **116**, 205-213, doi:10.1007/s00401-008-0408-9 (2008).

3 Robinson, J. L. *et al.* TDP-43 skeins show properties of amyloid in a subset of ALS cases. *Acta Neuropathol* **125**, 121-131, doi:10.1007/s00401-012-1055-8 (2013).

4 Thorpe, J. R., Tang, H., Atherton, J. & Cairns, N. J. Fine structural analysis of the neuronal inclusions of frontotemporal lobar degeneration with TDP-43 proteinopathy. *J Neural Transm (Vienna)* **115**, 1661-1671, doi:10.1007/s00702-008-0137-1 (2008).

5 Amador-Ortiz, C. *et al.* TDP-43 immunoreactivity in hippocampal sclerosis and Alzheimer's disease. *Ann Neurol* **61**, 435-445, doi:10.1002/ana.21154 (2007).

6 Laferrière, F. *et al.* TDP-43 extracted from frontotemporal lobar degeneration subject brains displays distinct aggregate assemblies and neurotoxic effects reflecting disease progression rates. *Nature Neuroscience* **22**, 65-77, doi:10.1038/s41593-018-0294-y (2019).

7 Zhu, L. *et al.* An ALS-mutant TDP-43 neurotoxic peptide adopts an anti-parallel β-structure and induces TDP-43 redistribution. *Hum Mol Genet* **23**, 6863-6877, doi:10.1093/hmg/ddu409 (2014).

8 Carlomagno, Y. *et al.* Casein kinase II induced polymerization of soluble TDP-43 into filaments is inhibited by heat shock proteins. *PLoS One* **9**, e90452, doi:10.1371/journal.pone.0090452 (2014).

9 Johnson, B. S. *et al.* TDP-43 is intrinsically aggregation-prone, and amyotrophic lateral sclerosis-linked mutations accelerate aggregation and increase toxicity. *J Biol Chem* **284**, 20329-20339, doi:10.1074/jbc.M109.010264 (2009).

10 Furukawa, Y., Kaneko, K., Watanabe, S., Yamanaka, K. & Nukina, N. A seeding reaction recapitulates intracellular formation of Sarkosyl-insoluble transactivation response element (TAR) DNA-binding protein-43 inclusions. *J Biol Chem* **286**, 18664-18672, doi:10.1074/jbc.M111.231209 (2011).

11 Vogler, T. O. *et al.* TDP-43 and RNA form amyloid-like myo-granules in regenerating muscle. *Nature* **563**, 508-513, doi:10.1038/s41586-018-0665-2 (2018).

12 Guo, L. *et al.* Nuclear-Import Receptors Reverse Aberrant Phase Transitions of RNA-Binding Proteins with Prion-like Domains. *Cell* **173**, 677-692.e620, doi:10.1016/j.cell.2018.03.002 (2018).

13 Shenoy, J. *et al.* Structural dissection of amyloid aggregates of TDP-43 and its C-terminal fragments TDP-35 and TDP-16. *Febs j* **287**, 2449-2467, doi:10.1111/febs.15159 (2020).

14 Capitini, C. *et al.* Full-length TDP-43 and its C-terminal domain form filaments in vitro having non-amyloid properties. *Amyloid* **28**, 56-65, doi:10.1080/13506129.2020.1826425 (2021).

15 Cao, Q., Boyer, D. R., Sawaya, M. R., Ge, P. & Eisenberg, D. S. Cryo-EM structures of four polymorphic TDP-43 amyloid cores. *Nat Struct Mol Biol* **26**, 619-627, doi:10.1038/s41594-019-0248-4 (2019).

16 Li, W. *et al.* Heat Shock-induced Phosphorylation of TAR DNA-binding Protein 43 (TDP-43) by MAPK/ERK Kinase Regulates TDP-43 Function. *J Biol Chem* **292**, 5089-5100, doi:10.1074/jbc.M116.753913 (2017).

17 McGurk, L., Gomes, E., Guo, L., Shorter, J. & Bonini, N. M. Poly(ADP-ribose) Engages the TDP-43 Nuclear-Localization Sequence to Regulate Granulo-Filamentous Aggregation. *Biochemistry* **57**, 6923-6926, doi:10.1021/acs.biochem.8b00910 (2018).

18 Porta, S. *et al.* Patient-derived frontotemporal lobar degeneration brain extracts induce formation and spreading of TDP-43 pathology in vivo. *Nat Commun* **9**, 4220, doi:10.1038/s41467-018-06548-9 (2018).
19 Chen, A. K. *et al.* Induction of amyloid fibrils by the C-terminal fragments of TDP-43 in amyotrophic lateral sclerosis. *J Am Chem Soc* **132**, 1186-1187, doi:10.1021/ja9066207 (2010).
20 Shimonaka, S., Nonaka, T., Suzuki, G., Hisanaga, S. & Hasegawa, M. Templated Aggregation of TAR DNA-binding Protein of 43 kDa (TDP-43) by Seeding with TDP-43 Peptide Fibrils. *J Biol Chem* **291**, 8896-8907, doi:10.1074/jbc.M115.713552 (2016).
21 Guo, W. *et al.* An ALS-associated mutation affecting TDP-43 enhances protein aggregation, fibril formation and neurotoxicity. *Nat Struct Mol Biol* **18**, 822-830, doi:10.1038/nsmb.2053 (2011).
22 Mompeán, M. *et al.* Structural Evidence of Amyloid Fibril Formation in the Putative Aggregation Domain of TDP-43. *J Phys Chem Lett* **6**, 2608-2615, doi:10.1021/acs.jpclett.5b00918 (2015).
23 Saini, A. & Chauhan, V. S. Delineation of the core aggregation sequences of TDP-43 C-terminal fragment. *Chembiochem* **12**, 2495-2501, doi:10.1002/cbic.201100427 (2011).
24 Jiang, L. L. *et al.* Structural transformation of the amyloidogenic core region of TDP-43 protein initiates its aggregation and cytoplasmic inclusion. *J Biol Chem* **288**, 19614-19624, doi:10.1074/jbc.M113.463828 (2013).

# Unmodified gels/blots for Supplementary Figures:

Supplementary Fig. 1D

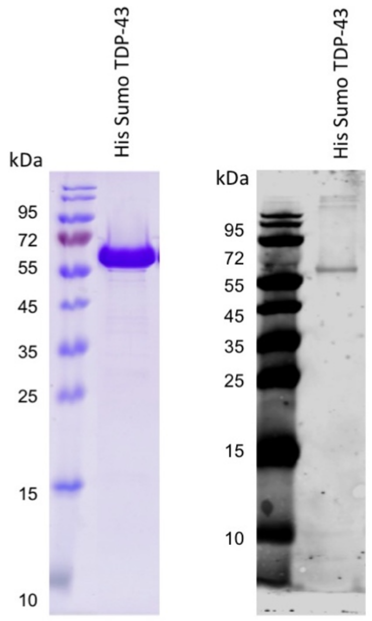


Supplementary Fig. 1E and F (outlined are used in the paper)

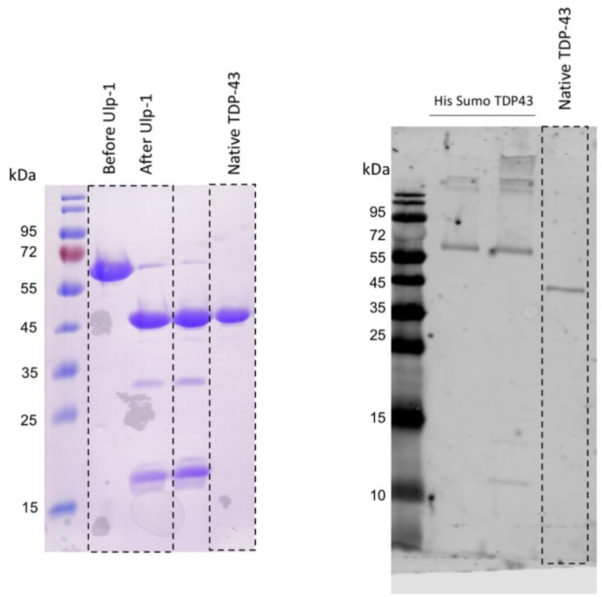

Supplementary Fig. 2B (outlined are used in the paper)

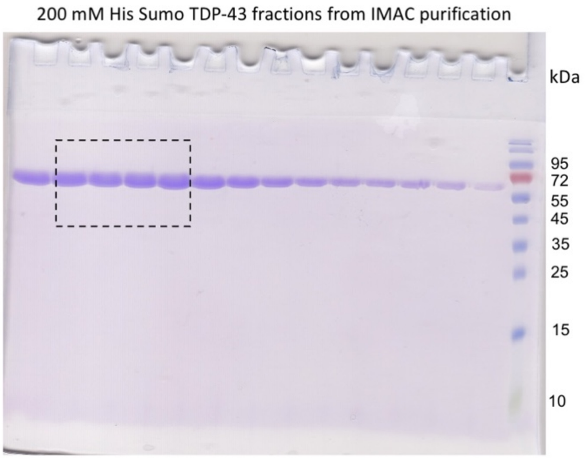


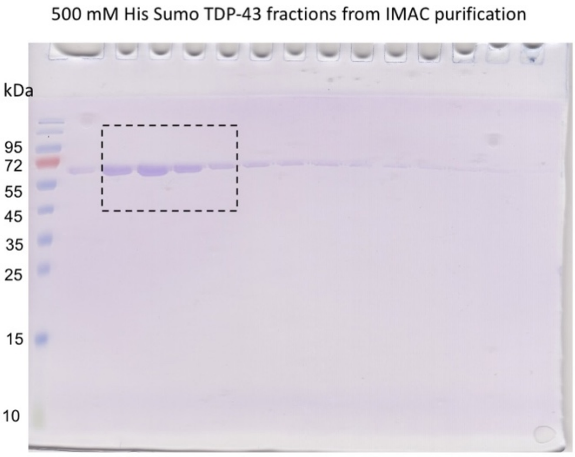

Supplementary Fig. 4B

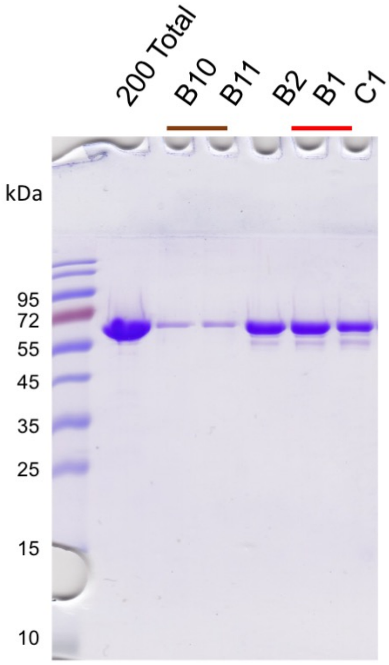


Supplementary Fig. 4D

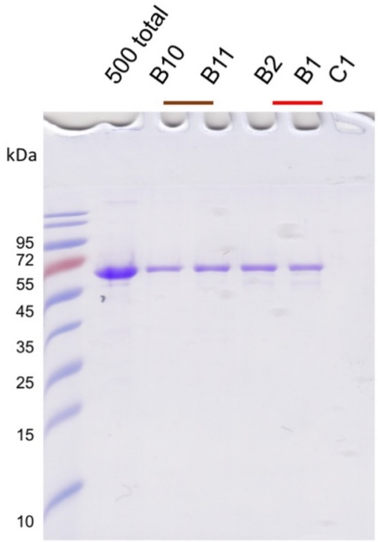

Supplement: Supplementary file 1 — Supplementary Figs. 1–11, Supplementary Tables 1–6, Supplementary references and source data of gels and immunoblots for Supplementary Figs. 1, 2 and 3 [file 41593_2023_1341_MOESM1_ESM.pdf]
