# Supplementary material for: Seeding the aggregation of TDP-43 requires post-fibrillization proteolytic cleavage
Source: Nat Neurosci. 2023 May 29;26(6):983–96. doi: 10.1038/s41593-023-01341-4 (PMC10244175; doi:10.1038/s41593-023-01341-4)
Supplement: Supplementary file 2 — Reporting Summary [file 41593_2023_1341_MOESM2_ESM.pdf]

nature portfolio

Corresponding author(s): Hilal A. Lashuel

Last updated by author(s): Apr 4, 2023

Reporting Summary

Nature Portfolio wishes to improve the reproducibility of the work that we publish. This form provides structure for consistency and transparency in reporting. For further information on Nature Portfolio policies, see our Editorial Policies and the Editorial Policy Checklist.

Statistics

For all statistical analyses, confirm that the following items are present in the figure legend, table legend, main text, or Methods section.

n/a	Confirmed	
☐	☒	The exact sample size (*n*) for each experimental group/condition, given as a discrete number and unit of measurement
☐	☒	A statement on whether measurements were taken from distinct samples or whether the same sample was measured repeatedly
☐	☒	The statistical test(s) used AND whether they are one- or two-sided<br>*Only common tests should be described solely by name; describe more complex techniques in the Methods section.*
☒	☐	A description of all covariates tested
☐	☒	A description of any assumptions or corrections, such as tests of normality and adjustment for multiple comparisons
☐	☒	A full description of the statistical parameters including central tendency (e.g. means) or other basic estimates (e.g. regression coefficient) AND variation (e.g. standard deviation) or associated estimates of uncertainty (e.g. confidence intervals)
☐	☒	For null hypothesis testing, the test statistic (e.g. *F*, *t*, *r*) with confidence intervals, effect sizes, degrees of freedom and *P* value noted<br>*Give P values as exact values whenever suitable.*
☒	☐	For Bayesian analysis, information on the choice of priors and Markov chain Monte Carlo settings
☒	☐	For hierarchical and complex designs, identification of the appropriate level for tests and full reporting of outcomes
☒	☐	Estimates of effect sizes (e.g. Cohen's *d*, Pearson's *r*), indicating how they were calculated

Our web collection on statistics for biologists contains articles on many of the points above.

Software and code

Policy information about availability of computer code

Data collection	Cryo-EM images were collected using the EPU 2.6.1 (ThermoFisher Scientific).<br>Mass spectrometry was performed using LTQ Orbitrap XL (Thermo Fisher, San Jose CA), equipped with an Accela Pump HPLC and a CTC™ThermoPAL™ autosampler (Thermo).
Data analysis	Cryo-EM images were analyzed using CTFFIND v4.1, crYOLO v1.7.5, RELION v3.1, COOT v0.9.8.6, PHENIX v1.19.2, MolRep v11.7.03, CCPEM 1.5.0. The final atomic model was validated using MolProbity.<br>Mass spectrometry raw data were converted into peak list (mzML) using ProteoWizard tools. Peptides were identified with the X! Tandem search engine as an internal tool of OpenMS v2.1 over the TOPPAS platform using TDP-43 as a sequence database.

For manuscripts utilizing custom algorithms or software that are central to the research but not yet described in published literature, software must be made available to editors and reviewers. We strongly encourage code deposition in a community repository (e.g. GitHub). See the Nature Portfolio guidelines for submitting code & software for further information.

Data

Policy information about availability of data

All manuscripts must include a data availability statement. This statement should provide the following information, where applicable:

- Accession codes, unique identifiers, or web links for publicly available datasets
- A description of any restrictions on data availability
- For clinical datasets or third party data, please ensure that the statement adheres to our policy

Raw cryo-EM images were deposited to the Electron Microscopy Public Image Archive (EMPIAR) with accession code EMPIAR-10840. Both cryo-EM maps of TDP-43 CP filaments were deposited to the Electron Microscopy Data Bank (EMDB) with accession code EMD-13795. Built atomic model was deposited to the Protein Data Bank (PDB) with accession code PDB ID 7Q3U. Most data from this study are available as Source data within this paper. All other data that are provided in the article and Supplementary information are available from the corresponding author on reasonable request.

Human research participants

Policy information about studies involving human research participants and Sex and Gender in Research.

Reporting on sex and gender	*Use the terms sex (biological attribute) and gender (shaped by social and cultural circumstances) carefully in order to avoid confusing both terms. Indicate if findings apply to only one sex or gender; describe whether sex and gender were considered in study design whether sex and/or gender was determined based on self-reporting or assigned and methods used. Provide in the source data disaggregated sex and gender data where this information has been collected, and consent has been obtained for sharing of individual-level data; provide overall numbers in this Reporting Summary. Please state if this information has not been collected. Report sex- and gender-based analyses where performed, justify reasons for lack of sex- and gender-based analysis.*
Population characteristics	A list of human brain tissues and demographic details associated with the ALS/FTLD-TDP patients and neurologically normal individuals is provided in Supplementary Table 3.
Recruitment	Samples were chosen based on the neuropathological confirmation and on age of death for both ALS/FTLD-TDP patients and neurologically normal individuals.
Ethics oversight	Human postmortem brains were obtained through the guidelines from the University of Pennsylvania, CNDR Brain Bank (doi:10.1016/j.jalz.2013.06.003). All necessary written informed consent forms were obtained from the patients or their next of kin in accordance with University of Pennsylvania Institutional Review Board guidelines and confirmed at the time of death.

Note that full information on the approval of the study protocol must also be provided in the manuscript.

Field-specific reporting

Please select the one below that is the best fit for your research. If you are not sure, read the appropriate sections before making your selection.

☒ Life sciences ☐ Behavioural & social sciences ☐ Ecological, evolutionary & environmental sciences

For a reference copy of the document with all sections, see nature.com/documents/nr-reporting-summary-flat.pdf

Life sciences study design

All studies must disclose on these points even when the disclosure is negative.

Sample size	No statistical methods were used to pre-determine sample sizes but our sample sizes are similar to those reported in previous publications (https://www.nature.com/articles/ncomms5824#Sec39; https://www.nature.com/articles/s41594-019-0248-4; https://www.science.org/doi/10.1126/sciadv.abn0044?url_ver=Z39.88-2003&rfr_id=ori:rid:crossref.org&rfr_dat=cr_pub%20%200pubmed; https://www.biorxiv.org/content/10.1101/500058v1; https://www.nature.com/articles/s41593-018-0294-y#Abs1). Minimum three repeats of independent sample preparation and analysis were used for protein purification, filament and fibrils preparation, negative-staining TEM, ThT kinetic assay, Immunogold labelling, primary cortical neuronal cultures and neuronal assays, and Eukaryotic cell line work, respectively. The number of cryo-EM micrographs collected was sufficient enough to generate high-resolution densities. Details of cryo-EM datasets are elaborated in the Methods section.
Data exclusions	No data were excluded from the analysis.
Replication	Minimum three independent experimental repeats were performed for the authenticity of the findings. All attempts at replication were successful.
Randomization	Randomization is not relevant for the biochemical, biophysical and cellular experiments that we performed in this study. The fact that we used the recombinantly prepared proteins and peptides and their assemblies, and studied about their properties using many biochemical,

biophysical and cell biology methods in vitro with independent repeats of 3 and more times, and with the calculation of statistics from the repeats where it can be derived, randomization is not relevant for this reason.

Blinding

Blinding was not relevant to our study. In general, blinding is not used when employing biochemical/biophysical experiments. It is also for the reason that the researcher who conducted the experiments also carried out the analyses of the results and interpretation. However, validation of the results were confirmed by different researchers employing different standard protocols/tools (for ex, TEM following proteolysis and cryo-EM; cell biology confirmation from two different investigators using two different cell lines/cultures).

Reporting for specific materials, systems and methods

We require information from authors about some types of materials, experimental systems and methods used in many studies. Here, indicate whether each material, system or method listed is relevant to your study. If you are not sure if a list item applies to your research, read the appropriate section before selecting a response.

Materials & experimental systems

n/a	Involved in the study	
☐	☒	Antibodies
☐	☒	Eukaryotic cell lines
☒	☐	Palaeontology and archaeology
☐	☒	Animals and other organisms
☒	☐	Clinical data
☒	☐	Dual use research of concern

Methods

n/a	Involved in the study	
☒	☐	ChIP-seq
☒	☐	Flow cytometry
☒	☐	MRI-based neuroimaging

Antibodies

Antibodies used

The primary antibodies used were
1) Rabbit polyclonal TDP43 antibody, Proteintech, 18280-1-AP
2) Rabbit polyclonal TDP-43 antibody, Proteintech, 10782-2-AP
3) Rabbit TDP-43 (C-terminal) Polyclonal antibody, Proteintech, 12892-1-AP
4) C-terminal anti-TDP-43 antibody (C2089) (1:3,000, in-house CNDR)
5) Monoclonal rat p409-410 specific TDP-43 antibody (TAR5P-1D3) 1:200, Ascenion, Munich (Germany)
6) Rabbit polyclonal antibody TMEM239, 1:2000 (a gift from MRC, Cambridge, UK)

Secondary antibodies used were
1) Goat anti-rabbit labeled with Alexa680, Invitrogen, A21109
2) Goat anti-rabbit IgG (H&L), Aurion, 810.011
3) Goat anti-rat IgG (H+L) Cross-Adsorbed Secondary Antibody, Alexa Fluor™ 647, ThermoFisher Scientific, A-21247

Validation

Commercial primary antibodies were validated by the manufacturer, see the links below for corresponding antibodies,
1) Rabbit polyclonal TDP43 antibody, Proteintech, 18280-1-AP (https://www.ptglab.com/products/TARDBP-Antibody-18280-1-AP.htm)
2) Rabbit polyclonal TDP-43 antibody, Proteintech, 10782-2-AP (https://www.ptglab.com/products/TARDBP-Antibody-10782-2-AP.htm)
3) Rabbit TDP-43 (C-terminal) Polyclonal antibody, Proteintech, 12892-1-AP (https://www.ptglab.com/products/TARDBP-Antibody-12892-1-AP.htm)
Relevant references for the validation of antibodies are given below for those obtained as gifts from other research groups
4) C-terminal anti-TDP-43 antibody (C2089) (https://www.nature.com/articles/s41467-018-06548-9)
5) Monoclonal rat p409-410 specific TDP-43 antibody (TAR5P-1D3) (https://link.springer.com/article/10.1007/s00401-008-0477-9#Sec2)
6) Rabbit polyclonal antibody TMEM239 (https://www.nature.com/articles/s41586-022-04650-z#Sec16)

Eukaryotic cell lines

Policy information about cell lines and Sex and Gender in Research

Cell line source(s)

QBI-293A cells were used to generate iGFP-NLSm cells. Source QBI-293A from Quantum (AES0506

Authentication

None of the cell lines used have been authenticated.

Mycoplasma contamination

QBI-293 cells and iGFP-NLSm derived cells were tested negative for mycoplasma contamination.

Commonly misidentified lines
(See ICLAC register)

No commonly misidentified cell lines were used in the study.

Animals and other research organisms

Policy information about studies involving animals; ARRIVE guidelines recommended for reporting animal research, and Sex and Gender in Research

Laboratory animals	P0 pups were used from the WT C57Bl/6JRj mice strain. Mice were housed on a 12-hour light-dark cycle at 22°C room temperature and 50% to 60% humidity with ad libitum access to food and water.
Wild animals	No wild animals were used in the study
Reporting on sex	Sex: mixed
Field-collected samples	No field collected samples were used in the study.
Ethics oversight	All experiments were approved by the Swiss Federal Veterinary Office (Authorization Nos. VD 3392).

Note that full information on the approval of the study protocol must also be provided in the manuscript.
