# Supplementary figures and images for: Seeding the aggregation of TDP-43 requires post-fibrillization proteolytic cleavage
Source: Nat Neurosci. 2023 May 29;26(6):983–96. doi: 10.1038/s41593-023-01341-4 (PMC10244175; doi:10.1038/s41593-023-01341-4)

### **Unmodified gels for Main Figure 1:**

Fig. 1B (Batch 2 is shown in the paper)

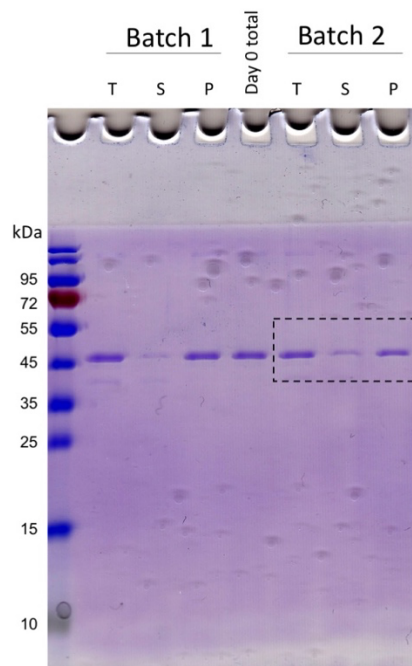

Supplement: Source Data Fig. 1 — Unprocessed gel. [file 41593_2023_1341_MOESM5_ESM.pdf]
